# The long zinc finger domain of PRDM9 forms a highly stable and long-lived complex with its DNA recognition sequence

**DOI:** 10.1007/s10577-017-9552-1

**Published:** 2017-02-02

**Authors:** Yasmin Striedner, Theresa Schwarz, Thomas Welte, Andreas Futschik, Ulrich Rant, Irene Tiemann-Boege

**Affiliations:** 1 Institute of Biophysics, Johannes Kepler University Linz, Gruberstrasse 40, 4020 Linz, Austria; 2 Dynamic Biosensors GmbH, 82152 Planegg, Germany; 3 Department of Applied Statistics, Johannes Kepler University, 4040 Linz, Austria

**Keywords:** enzyme kinetics, zinc finger, protein-DNA interaction, binding affinity, equilibrium dissociation constant, meiotic recombination, PRDM9, switchSENSE, gel mobility shift

## Abstract

PR domain containing protein 9 (PRDM9) is a meiosis-specific, multi-domain protein that regulates the location of recombination hotspots by targeting its DNA recognition sequence for double-strand breaks (DSBs). PRDM9 specifically recognizes DNA via its tandem array of zinc fingers (ZnFs), epigenetically marks the local chromatin by its histone methyltransferase activity, and is an important tether that brings the DNA into contact with the recombination initiation machinery. A strong correlation between PRDM9-ZnF variants and specific DNA motifs at recombination hotspots has been reported; however, the binding specificity and kinetics of the ZnF domain are still obscure. Using two in vitro methods, gel mobility shift assays and switchSENSE, a quantitative biophysical approach that measures binding rates in real time, we determined that the PRDM9-ZnF domain forms a highly stable and long-lived complex with its recognition sequence, with a dissociation halftime of many hours. The ZnF domain exhibits an equilibrium dissociation constant (*K*_D_) in the nanomolar (nM) range, with polymorphisms in the recognition sequence directly affecting the binding affinity. We also determined that alternative sequences (15–16 nucleotides in length) can be specifically bound by different subsets of the ZnF domain, explaining the binding plasticity of PRDM9 for different sequences. Finally, longer binding targets are preferred than predicted from the numbers of ZnFs contacting the DNA. Functionally, a long-lived complex translates into an enzymatically active PRDM9 at specific DNA-binding sites throughout meiotic prophase I that might be relevant in stabilizing the components of the recombination machinery to a specific DNA target until DSBs are initiated by Spo11.

**Electronic supplementary material:**

The online version of this article (doi:10.1007/s10577-017-9552-1) contains supplementary material, which is available to authorized users.

## Introduction

Meiotic homologous recombination mainly occurs in localized regions, known as recombination hotspots. In a large number of mammals including mice and humans, recombination hotspots have been shown to be associated with the activity of PR domain containing protein 9 (PRDM9﻿) (Berg et al., 2010, Baudat et al., 2010, Brick et al., 2012, Myers et al., 2010, Parvanov et al., 2010, Smagulova et al., 2011, Pratto et al., 2014).

PRDM9 is a meiosis-specific protein expressed in male and female germ cells entering meiotic prophase I (Hayashi and Matsui, 2006). PRDM9 is formed by the following functional domains: an N-terminal Krüppel-associated-box (KRAB) domain, a synovial sarcoma X repression domain (SSXRD) followed by a zinc knuckle, a protein arginine/Su (var)3–9 and “enhancer of zeste” (PR/SET) domain, a single separate zinc finger (ZnF), and finally a long tandem ZnF array at the C-terminal end with a variable number of ZnFs and diverse sets of DNA-contacting amino acid residues (reviewed in Baudat et al. (2013)). The current understanding of the molecular function of PRDM9 is that the tandem ZnF domain binds to specific DNA sequences (described in Baudat et al. (2010), Grey et al. (2011), and Myers et al. (2010)), the PR/SET domain mediates the tri-methylation of surrounding nucleosomes on lysine 4, as well as lysine 36 of histone 3 (Hayashi et al., 2005, Wu et al., 2013, Powers et al., 2016), and the KRAB domain recruits downstream proteins of the recombination initiation machinery (Parvanov et al., 2016).

The ZnFs are mostly of the C2H2 type (i.e., two cysteines and two histidines coordinate a single zinc atom), which have the consensus sequence F/Y_X_C_X_2–5__C_X_12__H_X_3–5__H (where X denotes any amino acid) (Wolfe et al., 2000). Based on the canonical binding model of C2H2-ZnFs, the DNA is contacted by the amino acids at positions −1, 2, 3, and 6 relative to the alpha helix (reviewed in Persikov et al., (2009) and Wolfe et al., (2000)). Interestingly, these amino acids are highly variable accounting for many PRDM9 variants that differ both in the number and identity of the ZnFs (Baudat et al., 2010, Parvanov et al., 2010, Thomas et al., 2009, Berg et al., 2010, Berg et al., 2011, Kong et al., 2010, Auton et al., 2012, Schwartz et al., 2014).

Based on these highly variable amino acids in the ZnFs, each PRDM9 variant has its own DNA recognition sequence which can be predicted by computational algorithms (Persikov et al., 2009, Persikov and Singh, 2014). In some cases, a subset of the binding sequence predicted by the Persikov algorithm is found enriched as a DNA motif in recombination hotspots identified by double-strand break (DSB) maps or historical linkage disequilibrium maps (Myers et al., 2010, Brick et al., 2012, Smagulova et al., 2011, Pratto et al., 2014). Evidence so far has shown that changes in the ZnF domain can turn on or off hotspots. In comparison, a few changes in the DNA recognition sequence modulate the intensity of the hotspot (e.g., mismatches to a consensus motif reduce the strength of the hotspot) (Smagulova et al., 2011, Pratto et al., 2014, Grey et al., 2011). This reduction in binding already by one difference in the recognition sequence strongly affects hotspot usage between two homologous sequences, resulting in an asymmetric hotspot distribution in hybrid crosses (Davies et al., 2016) and an initiation bias observed as meiotic drive in crossover products (Jeffreys and Neumann, 2002).

A recent structural analysis of a PRDM9-DNA complex revealed that certain amino acid-nucleotide contacts do not always follow the canonical binding model (Patel et al., 2016). Moreover, there is still considerable confusion about the correlation of PRDM9 recognition sequences (motifs) with the recombination activity. In humans, not all of the hotspots determined by sperm-typing contained the PRDM9-binding motif (Berg et al., 2010, Berg et al., 2011). It was also documented that polymorphisms in the binding motif explained only 44% of the variability in hotspots (Pratto et al., 2014). In addition, the human Myers motif or the murine target motifs are found more often outside than inside hotspots in the genome (Brick et al., 2012, Myers et al., 2010, Segurel et al., 2011). Finally, in vitro binding studies showed that the murine PRDM9^Cst^ bound to three different sequences (*Hlx1*, *Esrrg-1*, and *Psmb9*), which shared only a few nucleotides, with the latter two sequences binding with similar strength (Billings et al., 2013). Thus, the existing simple models of PRDM9 recognition of sequence motifs might not capture all aspects of binding site information. Moreover, it is still unclear what drives the binding specificity of the ZnF domain and what factors modulate the DNA recognition and complex formation.

For this reason, we analyzed the binding determinants and kinetics of the PRDM9-ZnF domain of the inbred mouse strain CAST/EiJ derived from the subspecies *Mus musculus castaneus* (harboring the *Prdm9* ^Cst^ allele that consists of 11 ZnF tandem repeats) with the well-characterized hotspot sequence *Hlx1* using two different in vitro approaches: electrophoretic mobility shift assays (EMSA) and switchSENSE, a technique that can accurately measure binding kinetics. We were able to gain first insights into the binding kinetics of the murine PRDM9-ZnF domain, which exhibits an equilibrium dissociation constant (*K*_D_) in the nanomolar (nM) range. Our kinetic data also show that once PRDM9 has encountered a specific DNA target, it forms a highly stable complex with dissociation halftimes between 9 and 17 h. We also tested different sequence arrangements of the hotspot DNA and determined that a minimal number of 15–16 nucleotides, contacting five consecutive ZnFs, are sufficient for a specific PRDM9-DNA interaction. Furthermore, we observed that the ZnF domain has a stronger preference for binding targets longer than the number of predicted bases contacted by all ZnFs of the array.

## Results

### PRDM9 binding kinetics show a slow dissociation of the PRDM9-DNA complex

**Fig. 1:**
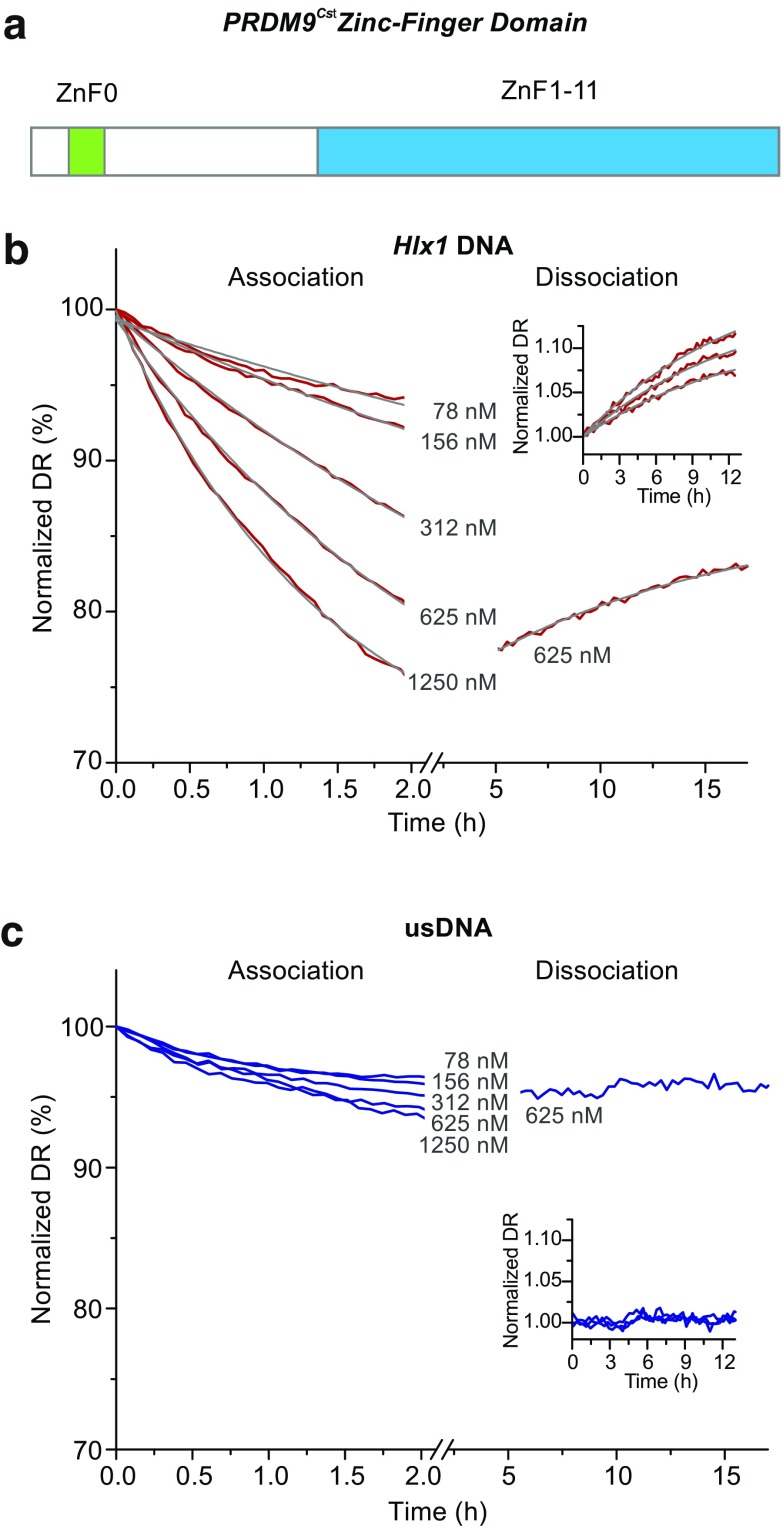
Binding kinetics of the PRDM9^Cst^-ZnF domain with two types of DNA measured with switchSENSE. **a** Schematic of the murine PRDM9^Cst^ zinc finger domain used in this work consisting of the DNA-binding C2H2-type zinc finger array (*ZnF1–11*) and an additional zinc finger (*ZnF0*) separated from the array. **b**, **c** Real-time association and dissociation of the PRDM9^Cst^-ZnF domain to a 48-bp long *Hlx1* ^B6^ and unspecific DNA (*usDNA*) sequence (panel **b** and **c**, respectively) shown as changes of the normalized dynamic response (dr; i.e., nanolever-switching speed). Different concentrations of PRDM9 (78, 156, 312, 625, and 1250 nM) were incubated for 2 h with the target DNA sequences, immobilized on a microchip. Complete sensor regeneration was performed between the different concentration steps. Poly(deoxyinosinic-deoxycytidylic) acid (*polydIdC*) (50 ng/μl) was added to each PRDM9 concentration step, as a non-specific competitor DNA. Only the PRDM9 incubation step at 625 nM was followed by a long dissociation measurement of ∼15 h, featuring intermittent measurements on three sensor spots (displayed in the *insets*). Differences in the dissociation curves of one sequence type are subject to a spot-to-spot variation, resulting in slightly different amplitudes. One of these triplicate measurements is displayed representatively in the *main panel*. The *gray lines* show the global exponential fits for the association rate constant (*k*_on_) or the dissociation rate constant (*k*_off_), respectively. Binding kinetics (*k*_on_, *k*_off_, and *K*_D_) derived from this data are shown in Table 1

Studying PRDM9 binding in vitro has been extremely difficult given the high content of sequence repeats, high cellular toxicity during bacterial protein expression, strong degradation during purification, and low solubility of the recombinant protein. We optimized the bacterial expression and lysate preparation of the PRDM9-ZnF domain (including the consecutive array of 11 ZnFs and the upstream separate single ZnF; see Fig. 1) of the strain CAST/EiJ (PRDM9^Cst^-ZnF) that rendered high enough yields of the protein for our experimental procedure (Supplementary_Fig_S1, panel A: Coomassie staining). The concentration of PRDM9^Cst^-ZnF in the lysates was determined by Capillary Western (Supplementary_Fig_S1, panel B). For each extract, the binding functionality of the PRDM9^Cst^-ZnF was first evaluated with EMSA. A positive evaluation was given when a complex formed with PRDM9^Cst^-ZnF and the *Hlx1* ^B6^ hotspot sequence (known to bind PRDM9^Cst^ (Billings et al., 2013)) observed as a shift, and no complex formed with an unspecific DNA control. In addition, we used also an in vitro expression system from a mammalian cell lysate for making different PRDM9 constructs, including the full-length PRDM9. Given that only small amounts of recombinant protein can be produced with the in vitro expression system, only a limited number of experiments were performed. However, the different in vitro extracts rendered complexes with equivalent migration distances and similar intensities in EMSA as the bacterial extracts (see Supplementary_Fig_S2). This suggests that the observed binding patterns are comparable between expression systems, and if components in the extract are important for the formation of the PRDM9-DNA complex, then they are present in all expression systems and are likely to be representative of the in vivo system.

In order to gain more insight about the PRDM9-DNA interaction, we assessed the binding kinetics of the ZnF domain of PRDM9^Cst^ in a real-time approach employing the switchSENSE technology. switchSENSE is a surface-based technology, where DNA nanolevers are immobilized on gold microelectrodes (sensor spots) on one end and carry a fluorescent label on the other. When applying alternating voltage, the DNA nanolevers switch their orientation being repelled and attracted to the gold surface. This surface acts as an acceptor for a non-radiative energy transfer causing a quenching effect on the DNA fluorophore when the DNA nanolevers are in close proximity. With this principle, the switching speed of the DNA can be monitored in real time by a time-resolved single-photon-counting module (Langer et al., 2013). Association and dissociation rates of a ligand to the DNA can be measured by comparing the change in the nanolever’s switching dynamics, given that the hydrodynamic friction increases with the complex formation.

We measured the binding kinetics with switchSENSE using different lysate concentrations (starting with the lowest concentration) for two different DNA targets: the *Hlx1* ^B6^ hotspot sequence and an unspecific DNA (usDNA) sequence, as a negative control (Fig. 1). The drop in dynamic response observed at different lysate concentrations reflected the number of complexes formed on the immobilized DNA on the sensor. This experiment was performed with complete sensor regeneration between the different concentration steps. This means that the protein and the DNA strand, which is not immobilized on the surface, were washed off completely and the single-stranded DNA nanolevers were hybridized again with new DNA. The switching speed of the association phase was recorded for several hours before the dissociation phase was measured in the buffer flow over ∼15 h. The dissociation was assessed only for one of the highest PRDM9 concentrations, since this parameter is concentration independent. However, the dissociation measurement was performed in triplicates by measuring all three sensor spots alternatingly. The dynamic response was plotted against time to determine the kinetic rate constants. The dissociation rate constant (*k*_off_) was determined by a global exponential fit for the triplicate measurements and the association rate constant (*k*_on_) by a global exponential fit over all protein concentrations (see “Experimental procedures”). The obtained data show a sequence-specific binding of the PRDM9^Cst^-ZnF to the mouse hotspot *Hlx1* ^B6^, while no significant interaction was observed with the unspecific DNA substrate (Fig. 1). The PRDM9^Cst^-ZnF-*Hlx1* ^B6^ complex is formed with an association rate constant (*k*_on_) of 135 M^−1^ s^−1^ and dissociates with a rate constant (*k*_off_) of 2.2 × 10^−5^ s^−1^ (Table 1).

**Table 1:** Kinetic data of three switchSENSE experiments

PRDM9^Cst^ZnF-Hlx1^B6^ complex	*k*_on_ [M^−1^ s^−1^]^a^	*k*_off_ [s^−1^]^b^	*t*_1/2(off)_ [h]	*K*_D_ [nM]	Comment^c^
Measurement 1 (Fig. 1b)	135 ± 5	2.2 ± 0.2 × 10^−5 d^	∼9 h	159.7 ± 1.2	Constant (high) polydIdC
Measurement 2 (Supplementary_Fig_S3 A)	725 ± 35	1.1 ± 0.3 × 10^−5^	∼17 h	15.7 ± 4.1	Titrated (low) polydIdC
Measurement 3 (Supplementary_Fig_S3 B)	1908 ± 130	1.4 ± 0.1 × 10^−5^	∼14 h	7.2 ± 0.8	Titrated (low) polydIdC
EMSA measurements (Fig. 3b)	n.a.	n.a.	n.a.	24.5 ± 2.6	Titrated (low) polydIdC

*n* *.a.* not available

^a^Association rate constants (*k*_on_) were derived by global exponential fits of the association traces of different PRDM9 concentrations

^b^Dissociation rate constants were derived by either global or individual exponential fits of the dissociation traces as indicated

^c^Different concentrations of polydIdC were used in these binding reactions with either a constant amount of 50 ng/μl polydIdC (constant, high polydIdC) or no polydIdC (titrated, low polydIdC) supplemented to the sample buffer but diluted from the protein stock with every PRDM9 concentration (highest polydIdC concentration 9.4 ng/μl for 4360 nM PRDM9). Note that the protein stock extract had a concentration of 22.84 μM PRDM9 and 47.62 ng/μl polydIdC

^d^Measured in triplicates on sensor spots 1–3 (global fit)

Based on the dissociation rate constant, the dissociation halftime of the PRDM9-DNA complex can be calculated (given by *t*_1/2(off)_ = ln2/*k*_off_) (Hulme and Trevethick, 2010), which is approximately 9 h, indicating that the PRDM9-ZnF domain forms a highly stable and long-lived complex with DNA.

We obtained kinetic data of three independent switchSENSE measurements for the PRDM9^Cst^-ZnF-*Hlx1* ^B6^ interaction using sample buffer conditions differing mainly in poly(deoxyinosinic-deoxycytidylic) acid (polydIdC) concentrations (Table 1). PolydIdC is a low-complexity DNA substrate commonly used in DNA-binding studies to saturate other DNA-binding proteins in cell lysates (Larouche et al., 1996). switchSENSE measurements without additional polydIdC in the sample buffer, but diluted from the protein stock extract with <10 ng/μl for the highest PRDM9 concentration (low polydIdC condition), rendered consistently faster association rate constants than obtained when using 50 ng/μl polydIdC (high, constant polydIdC condition) in the sample buffer (725 to 1908 vs. 135 M^−1^ s^−1^, respectively). The dissociation rate remained within the same range (1.1 × 10^−5^ to 2.2 × 10^−5^ s^−1^) regardless of the polydIdC in the reaction, yielding dissociation halftimes (*t*_1/2(off)_) between 9 and 17 h.

The affinity of an interaction (given by the equilibrium dissociation constant *K*_D_) can also be calculated from the switchSENSE kinetic data with the equation *K*_D_ = *k*_off_/*k*_on_. It ranged from ∼7 to ∼16 nM at low and ∼160 nM at high polydIdC conditions (Table 1, Supplementary_Fig_S3). The overall lower affinity of the PRDM9^Cst^-ZnF to the *Hlx1* ^B6^ DNA at high polydIdC concentrations is a direct consequence of the resulting lower association rate constant. It is possible that excess polydIdC also binds to PRDM9, lowering the effective PRDM9 concentration that interacts with the DNA nanolevers, which translates into a reduced association rate constant. The association rate constants were susceptible to the different polydIdC conditions used; however, the dissociation rates are consistently slow between measurements and variations are independent of polydIdC concentrations. This suggests that once PRDM9^Cst^ has bound to a specific DNA substrate, it shows a residence time of several hours, regardless of the initial binding conditions. It is possible that other PRDM9 variants have varying dissociation times for different sequences. Moreover, we cannot infer from our data whether changes in the affinity of an interaction (*K*_D_) is the result of differences in the *k*_on_, *k*_off_, or both. Further measurements on different PRDM9 variants and target sequences would be required to properly answer this question.

### PRDM9 forms a highly stable complex with DNA upon specific binding

**Fig. 2:**
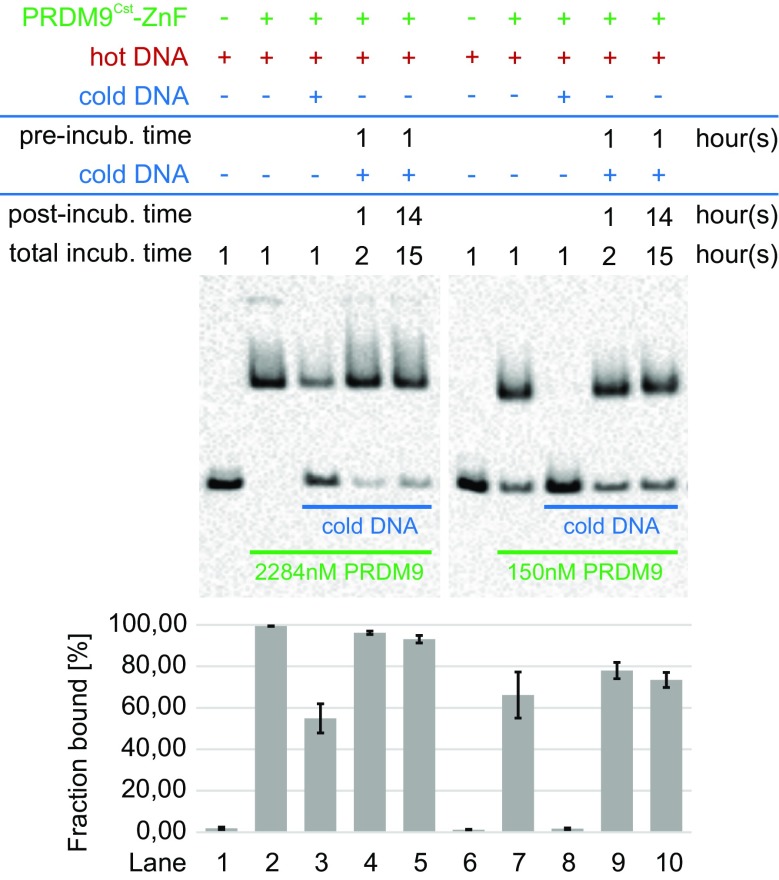
PRDM9-DNA-complex stability assessed with an EMSA competition assay. Different concentrations of the PRDM9^Cst^-ZnF domain (2284 or 150 nM) were incubated with 10 nM of a biotinylated 75 bp DNA (hot *Hlx1* ^B6^) and a 100-fold excess of 39 bp competitor DNA without biotin (cold *Hlx1* ^B6^). The competitor was added to the hot *Hlx1* ^B6^ at different time points. *Lanes 2* and *7* show the incubation of PRDM9 only with hot *Hlx1* ^B6^. In comparison, *lanes 3* and *8* represent the simultaneous incubation of PRDM9 with hot and cold *Hlx1* ^B6^ and *lanes 4 + 5* and *9 + 10* show the pre-incubation of PRDM9 with hot *Hlx1* ^B6^ for 1 h before adding the excess cold *Hlx1* ^B6^ to the reaction, which was then stopped either after 1 or ∼14 h. The average percentage of hot *Hlx1* ^B6^ in the PRDM9-DNA complex (% fraction bound) was estimated as the ratio of pixel intensities of the shifted band to the sum of free and bound hot *Hlx1* ^B6^. *Error bars* represent the standard deviation of two independent experiments. The length difference between hot and cold *Hlx1* ^B6^ was important for the proper blotting of the free hot *Hlx1* ^B6^ used to quantitate the complex (fraction bound [%])

The slow dissociation of the PRDM9^Cst^-*Hlx1* ^B6^ complex, measured with the switchSENSE technology, was corroborated in a solution binding experiment (EMSA) (Fig. 2). For this experiment, we first incubated a large excess of the PRDM9^Cst^-ZnF lysate (2284 or 150 nM, respectively) with 10 nM biotinylated *Hlx1* ^B6^ DNA (hot *Hlx1* ^B6^) for 1 h. This time frame was chosen according to a time-course EMSA that showed that 1 h is sufficient for both high and low PRDM9 concentrations to reach an equilibrium binding state with the DNA, considering that the apparent association is faster with higher PRDM9 concentrations (Supplementary_Fig_S4). We also added a 100-fold higher concentration of un-biotinylated *Hlx1* ^B6^ DNA (1 μM cold *Hlx1* ^B6^), either simultaneously or consecutively, and then incubated the reaction for 1 h or overnight. The key difference between hot and cold *Hlx1* ^B6^ is that only the hot DNA (biotinylated DNA) can be visualized in EMSA. If the hot *Hlx1* ^B6^ falls off rapidly from the complex, then over time, the excess cold *Hlx1* ^B6^ should substitute the hot *Hlx1* ^B6^ in the complex, resulting in a less visible complex (shifted band) and more visible free DNA. When incubating hot *Hlx1* ^B6^ and cold *Hlx1* ^B6^ simultaneously (lanes 3 and 8), the intensity of the visible complex was much weaker compared to the reaction with only hot *Hlx1* ^B6^ (no cold competitor; lanes 2 and 7), an expected result since stochastically, more binding sites were filled by cold *Hlx1* ^B6^ added in 100-fold excess. Almost 100% of the hot *Hlx1* ^B6^ was visible as free DNA for the experiment with the lower PRDM9 concentration (150 nM), since the excess cold *Hlx1* ^B6^ likely fully saturated the PRDM9-binding sites and formed the complex.

In contrast, the consecutive addition of hot *Hlx1* ^B6^ followed by cold *Hlx1* ^B6^ (lane 4 + 5 and 9 + 10) showed a very different pattern. When pre-incubating the hot *Hlx1* ^B6^ with the PRDM9^Cst^-ZnF lysate for 1 h before adding the excess of the cold competitor, the intensity of the complex hardly changed, even after 15 h long incubation times. These experiments reflect that there is hardly an exchange between the bound (hot *Hlx1* ^B6^) in the complex and the free DNA (cold *Hlx1* ^B6^). Note that the cold *Hlx1* ^B6^ is only 39 bp long compared to the 75 bp hot *Hlx1* ^B6^ because excess cold DNA saturates the blotting membrane reducing considerably the signal of the hot free DNA band if both are of the same length. The length difference of the DNA sequence could explain why we did not observe a larger exchange between the hot and cold DNA given a dissociation half-life of 9–17 h estimated with switchSENSE. However, both experimental systems support the observation that PRDM9 forms a very stable complex with specific DNA that is fairly long-lived and that the dissociation of the PRDM9-DNA complex is very slow.

### Binding affinity of PRDM9 in solution is consistent with switchSENSE measurements

**Fig. 3:**
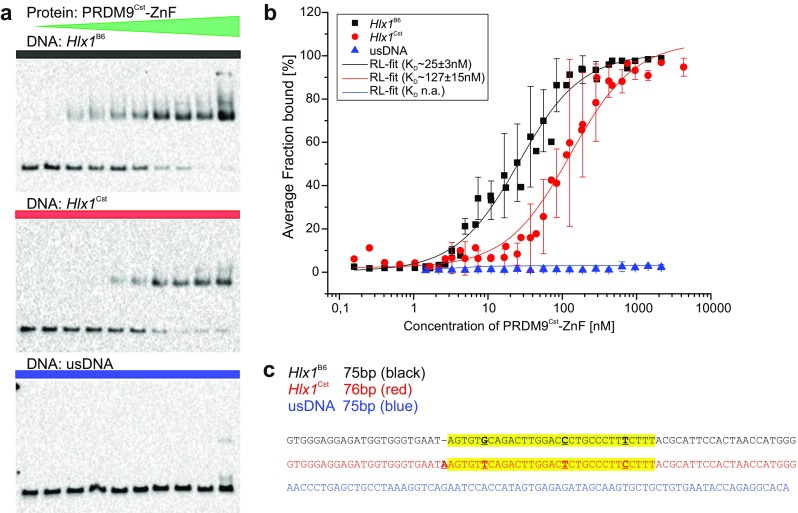
Binding affinity of PRDM9 to DNA in solution assessed with an EMSA titration assay. **a** Increasing concentrations of PRDM9^Cst^-ZnF (0.16–4300 nM) were incubated with 3 nM of different biotinylated target DNAs (*Hlx1* ^B6^_75bp shown in *black*, *Hlx1* ^Cst^_76bp in *red*, and usDNA_75bp in *blue*, respectively) for ∼90 h, containing increasing concentrations of polydIdC (0.00035–9.39 ng/μl) and 0.03% sarcosyl (*N*-lauroylsarcosine). Shown is a representative EMSA for *Hlx1* ^B6^ (*top*), *Hlx1* ^Cst^ (*middle*), and usDNA (*bottom*) of triplicate (*Hlx1*) or duplicate (usDNA) measurements. The average percent of the PRDM9-DNA complexes (% fraction bound) was calculated as the ratio of pixel intensities of the shifted band to the sum of free and bound DNA. **b** The average fraction bound of two (usDNA) or three (*Hlx1* ^B6^ and *Hlx1* ^Cst^) independent experiments was plotted against the PRDM9 concentration in a semi-logarithmic graph and the equilibrium dissociation constant (*K*_D_) was derived using a function that describes receptor-ligand binding in solution in dependence of the concentration of the labeled compound (see Supplementary_Methods). The *error bars* represent the standard deviation of two or three independent experiments. **c** Shown is one strand of the DNA sequences tested: *Hlx1* ^B6^, *Hlx1* ^Cst^, and usDNA in *black*, *red*, and *blue*, respectively. All DNA sequences are shown in 5′–3′ direction. The specific target site of the PRDM9^Cst^-ZnF array in the *Hlx1* hotspots is highlighted in *yellow*. The polymorphisms between *Hlx1* ^B6^ and *Hlx1* ^Cst^ are highlighted as *bold*, *underlined letters*

We also assessed the affinity of the PRDM9^Cst^-ZnF binding to the 75 base pairs (bp) *Hlx1* ^B6^ DNA by EMSAs. In these experiments, the affinity of a receptor-ligand interaction can be mathematically derived at the equilibrium stage, where the equilibrium dissociation constant (*K*_D_) is defined as the receptor (PRDM9) concentration at which 50% of the ligand (labeled DNA) is bound and 50% is free (visualized by EMSA). For these experiments, a decreasing PRDM9 concentration series was titrated with a constant amount of labeled *Hlx1* ^B6^ DNA (Fig. 3a, b).

In order to ensure equilibrium conditions, the incubation times of the lysate with the target DNA were chosen to be ∼10× the halftimes of the equilibrium reaction (the time at which half of the complex has formed, given by *t*_1/2_ = ln2/(*k*_on_ * [PRDM9] + *k*_off_) based on the switchSENSE kinetic information with the lowest association rate constant under comparable conditions (Table 1, measurement 2). Next, we optimized the DNA concentration (the visible substrate) to be much lower than the *K*_D_ ([DNA] < < *K*_D_) obtained from switchSENSE in order to use non-saturating conditions of the protein and avoid possible stoichiometric effects influencing the complex formation. The fraction bound was then calculated from the quantitative readout of the pixel intensities of the shifted band (complex) compared to the free DNA. The *K*_D_ was estimated by fitting the fraction bound plotted against the PRDM9^Cst^-ZnF concentration for data of three individual experiments. Note that there was a difference in the DNA lengths used in switchSENSE and EMSA (48 and 75 bp, respectively). In addition, we used the same binding buffer with no additional polydIdC (low polydIdC condition) as in switchSENSE, but the buffer in EMSA reactions were supplemented with 0.03% sarcosyl (*N*-lauroylsarcosine).

Our estimated *K*_D_ for the binding of PRDM9^Cst^-ZnF to the *Hlx1* ^B6^ DNA was 25 ± 3 nM in the EMSA experiments (Fig. 3; Table 1), which is comparable to the *K*_D_ obtained in the switchSENSE experiment under similar conditions. In addition, the binding of PRDM9^Cst^-ZnF to an usDNA sequence did not result in a specific interaction, corroborating the switchSENSE data. We also investigated whether the binding of PRDM9 to DNA is influenced by the other domains of PRDM9 in addition to the ZnFs by assessing the binding of full-length PRDM9 (PRDM9-FL) via EMSA. In a qualitative experiment, PRDM9-FL (expressed in vitro) rendered a similar binding as PRDM9^Cst^-ZnF (Supplementary_Fig_S2). This suggests that the main domain of PRDM9 interacting with the naked DNA is the ZnF domain; however, this does not preclude that the other domains of PRDM9 might influence the binding in the context of proteins present in the meiotic cell.

### Binding affinity of PRDM9 varies between DNA sequences

Using the EMSA titration assays, we also compared the affinity of the PRDM9^Cst^-ZnF to the same DNA region found in different related mice (C57BL/6J, referred to as B6 mice derived from the subspecies *Mus musculus domesticus* and mouse strain CAST/EiJ derived from the subspecies *Mus musculus castaneus*, short CST). The inbred mouse strain C57BL/6J (B6) harbors the PRDM9^B6^ variant of *M. m. domesticus*, which was separated genetically from *M. m. castaneus* (CST) 0.5 million years ago (Oliver et al., 2009). Thus, the genome sequence of a B6-mouse can be considered as naïve (virgin) to the PRDM9^Cst^ variant. *Hlx1* ^Cst^ differs from the *Hlx1* ^B6^ sequence by three SNPs and one in-del (Fig. 3c). Interestingly, the affinity of PRDM9^Cst^-ZnF to its own target sequence (*Hlx1* ^Cst^), which has been exposed to this PRDM9 allele continuously (in an evolutionary sense), is ∼5× weaker than to the virgin DNA of the B6 mice (*Hlx1* ^B6^), as shown in Fig. 3a, b (black and red curves). This is consistent with the hypothesis that DNA sequences at recombination hotspots erode (Myers et al., 2010), due to the formation of constant double-strand breaks at PRDM9 recognition sites that are subject to meiotic drive (Jeffreys and Neumann, 2002), GC-biased gene conversion (Duret and Galtier, 2009, Arbeithuber et al., 2015), and mutagenesis (Arbeithuber et al., 2015). The immediate consequence of this sequence evolution at hotspots is that the PRDM9-ZnF array has a lower binding affinity to its own eroded hotspot sequences than to naïve (virgin) sequences, as we observed for the *Hlx1* hotspot, also explaining the asymmetry of hotspot usage in hybrid crosses (Davies et al., 2016). The strong effect of polymorphisms in the binding affinity was also shown for the human ZnF array, with one polymorphism having a ∼3× to >10× change in affinity depending on the ZnF array tested (Patel et al., 2016).

### How many ZnFs of PRDM9 are necessary for a specific binding?

There are still several bizarre observations about the mode of action of PRDM9, reporting on active hotspots without the common PRDM9 sequence motif (Berg et al., 2010, Pratto et al., 2014) or PRDM9 motifs outside hotspot regions (Segurel et al., 2011, Walker et al., 2015). Also, in vitro assays have shown that the same PRDM9 allele can have similar binding strengths for very different sequences (Billings et al., 2013). This has been described as the “PRDM9 binding paradox,” where PRDM9 is specific for its recognition sequence and, at the same time, permissive for divergent sequences (Segurel et al., 2011).

In order to understand the binding plasticity of PRDM9, we designed an experiment to assess if different subsets of the ZnF array can recognize specific sequence stretches. At least 31 nucleotides are contacted by the PRDM9^Cst^-ZnF array (called here “target site”), and individual substitutions in this target site affect the binding (some more than others), as shown in the previous experiment and other works (Billings et al., 2013, Grey et al., 2011). However, it is still unclear if PRDM9 can bind different stretches of DNA or can recognize different DNA motifs given the large number of ZnFs in the domain. For this purpose, we consecutively replaced the 31 bp *Hlx1* ^B6^ target site by five-nucleotide steps with an usDNA sequence shown not to bind PRDM9 in our experiments (EMSA and switchSENSE). These chimeric DNA sequences have the same length, but the number and position of the specific nucleotides within the target sequence were varied in order to explore how many ZnFs are necessary to confer a specific binding. Using smaller steps would have been more intuitive to examine the participation of each ZnF in the sequence-specific binding (e.g., three nucleotides make base-specific contact with one ZnF), but would have made this experiment unnecessarily large and difficult to handle with no further gain in information to a previous study (Billings et al., 2013).

For these experiments, we also used non-saturating reaction conditions of the protein based on the same rationale as the EMSA binding affinity experiments. Note that at these conditions, the largest effect is at ∼30–70% of complex formation; whereas, larger changes in affinity might not be obvious at conditions at <10 or >90% of complex formation and should be interpreted with care. For this reason, we used conditions that rendered 100% complex formation for the reference sequence *Hlx* ^*B6*^ (2.5 μM PRDM9 incubated for 20 min with 15 nM of DNA; Supplementary_Fig_S4), but chimeras with lower affinity formed ideally at ∼50% of the complex.

**Fig. 4:**
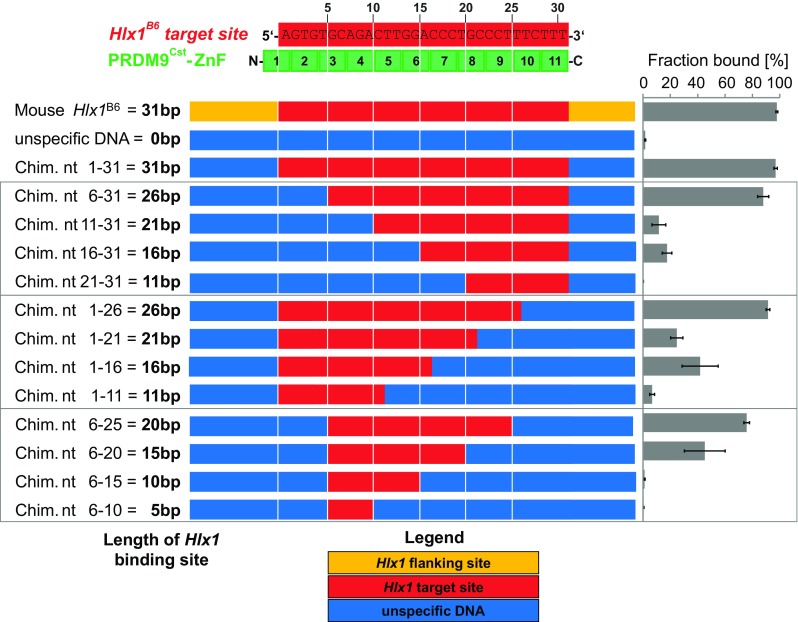
Binding specificity of the PRDM9 ZnF domain. Shown is the alignment of the *Hlx1* ^*B6*^ binding site and the PRDM9^Cst^-ZnF repeats. Each zinc finger contacts three nucleotides of the binding site. The *numbers* indicate the ZnFs’ position to the respective nucleotide sequence. The complex formation was assessed by incubating for 20 min 2.5 μM PRDM9^Cst^-ZnF (YFP tagged) with 15 nM of different arrangements of the *Hlx1* ^B6^ sequence (10 mM Tris-HCl pH 7.5, 50 mM KCl, 1 mM DTT, 50 ng/μl polydIdC, 0.05% NP-40, 50 μM ZnCl_2_) and evaluated by EMSA. The used DNA concentration was optimized to be much lower than the *K*_D_ of the substrate of the highest affinity to ensure non-saturating conditions. The flanking sites (*orange*) were replaced by an unspecific DNA sequence (*blue*) to create the chimera fragment nt 1–31, which contains the 31 bp target site (*red*) of PRDM9^Cst^. Furthermore, this target site (*red*) was replaced in five-nucleotide steps by an unspecific DNA sequence (*blue*), as shown. The fraction bound was calculated as percentage of the complex (ratio of pixel intensities of the shifted band to the sum of free and bound DNA). The *error bars* represent the standard deviation of three independent experiments

First, we only replaced the flanking regions of the specific target site with the usDNA, referred to as *Chimera nt* 1*–*31 *bp* (contacting ZnFs2–11 and partially ZnF1), which did not show a reduced binding to PRDM9^Cst^-ZnF, as compared to the 75 bp mouse *Hlx1* ^B6^ DNA (Fig. 4). According to the ZnF-DNA alignment of a previous publication (Billings et al., 2013), the upstream flanking region could be contacted by ZnF1 in the PRDM9^Cst^ tandem array (which is a degenerate “CH2” rather than a C2H2-type ZnF, missing a zinc coordinating cysteine residue). Given that we see no difference in the binding when replacing the flanking region with an unspecific sequence, ZnF1 either does not bind to DNA or the binding is not sequence specific. Next, we further replaced five nucleotides from either the 5′ or the 3′ end of the target site (*Chim. nt* 6–31 or *Chim. nt* 1–26 contacting ZnFs3/4–11 and ZnFs2–9, respectively), which had only a slight effect on the PRDM9 binding, suggesting that eight or nine ZnFs are still sufficient to confer binding specificity. A further substitution of 10–15 bp at the 5′ end of the target site resulted in a significantly reduced binding (*Chim. nt* 11–31 and *Chim. nt* 16–31 contacting ZnFs5–11 and ZnFs7–11). This effect was also observed for 3′-end substitutions (*Chim. nt* 1–21 and *Chim. nt* 1–16 contacting ZnFs2–7 and ZnFs2–6), albeit with a less pronounced effect. Finally, chimeras with replacements of 20–25 bp of the target site (*Chim. nt* 21–31, *Chim. nt* 1–11, *Chim. nt* 6–15, and *Chim. nt.* 6–10 contacting ZnFs8/9–11, ZnFs2–4, ZnFs3/4–5/6, and ZnFs3/4, respectively) show a strongly reduced binding to PRDM9^Cst^, indicating that two to four ZnFs of PRDM9^Cst^ are not sufficient to confer binding specificity.

The 15 to 16 bp different specific sequences at either end of the target site (i.e., *Chim. nt 16–31* and *Chim. nt 1–16* contacting ZnFs7–11 and ZnFs2–6, respectively) constitute about half of the target site (overlapping by only one base), yet both DNA fragments still display a binding to PRDM9^Cst^, albeit with a lower affinity than the reference chimera (*Chim. nt* 1–31). Also, *Chim. nt* 6–20 (contacting ZnFs3/4–7) displays a similarly reduced binding behavior. Taken together, these data suggest that a minimal number of 15–16 nucleotides contacted by five fingers are still sufficient to confer binding specificity (some ZnF combinations more than the others). Furthermore, these results reflect the binding plasticity of the PRDM9-ZnF array, where binding specificity of similar strength can be conferred by multiple subsets of the PRDM9-ZnF domain.

Recently, it was also reported that a shortened version of the ZnF domain of the human PRDM9^A^ variant (ZnFs8–12) already binds specifically to the degenerate Myers motif (“CCnCCnTnnCCnC”) within the THE1B retrotransposon (Patel et al., 2016). Furthermore, the crystal structure of this ZnF-DNA complex revealed that, in addition to the specific nucleotide-amino acid contacts with the consensus bases, the bases at the variable (“*n*”) positions engage in hydrogen bonds with amino acids in the ZnFs, which appear to be optional contacts that are beneficial, but not essential for complex formation (Patel et al., 2016). Also, interactions between amino acids and the DNA-phosphate backbone are relevant in the complex formation or stability (Patel et al., 2016, Billings et al., 2013).

### The binding of PRDM9 increases with the length of the DNA

**Fig. 5:**
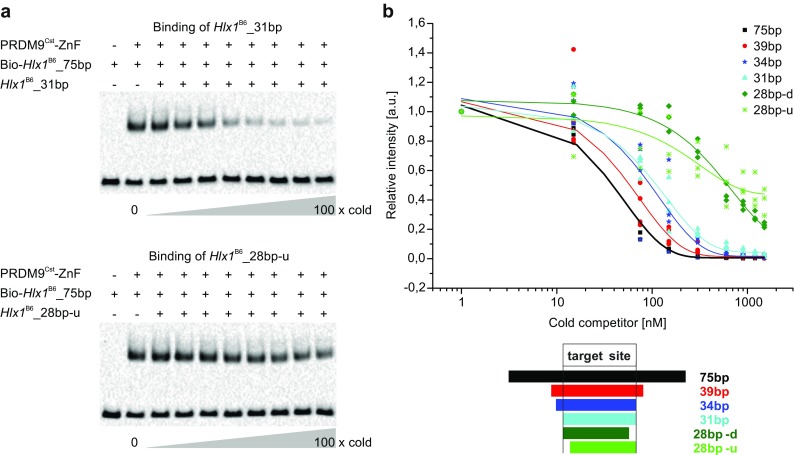
PRDM9 binding to different lengths of DNA. **a** In this competition EMSA, 250 nM PRDM9^Cst^-ZnF (YFP tagged) was incubated simultaneously with hot (biotinylated) reference DNA (75 bp *Hlx1* ^B6^) and different ratios of excess cold *Hlx1* ^B6^ for 1 h (a time frame that showed unchanged complex amounts; Supplementary_Fig_S4). We used for each experiment different lengths of cold DNA *(75 bp*, *39 bp*, *34 bp*, *31 bp*, *28 bp-d*, and *28 bp-u* sequences are shown in Supplementary_Table_S1, section C*)* representing truncations of the 75 bp *Hlx1* ^B6^ sequence. Shown are representative EMSAs for the cold competitors with 31 and 28 bp. The measurements were performed in triplicates for each cold competitor. **b** Intensities of the shifted bands are calculated relative to the reference PRDM9^Cst^-*Hlx1* ^B6^ complex without the addition of cold DNA (*lane 2*) and are plotted as relative intensities against the concentration of the cold competitor in a semi-logarithmic graph. *28 bp-d* and *28 bp-u* indicate 28 base-pair long fragments with truncations of three bases from the target binding site at either the downstream (3′ end) or the upstream (5′ end) end, respectively

The next question we investigated is if all of the ZnFs in the array are involved in the binding of the DNA (specifically or unspecifically). Previous work reported that at least 31 nucleotides are necessary for the complex formation with the PRDM9^Cst^-ZnF array suggesting that 10 out of the 11 zinc fingers of the PRDM9^Cst^ array are required (Billings et al., 2013). We tested the binding of the PRDM9^Cst^-ZnF to *Hlx1* ^B6^ DNA of different lengths with an EMSA competition experiment (Fig. 5). In this experiment, the complex formation was monitored with the same DNA (75 bp hot *Hlx1* ^B6^) incubated simultaneously with different concentrations of cold *Hlx1* ^B6^ of varying lengths (28 to 75 bp). We then used the competition efficiency across several ratios of hot/cold DNA as a parameter to evaluate differences in binding affinity. With this setup, we could compare the complex formation across a large dynamic range of competitor, which allowed a more quantitative evaluation of binding differences with DNA length. We monitored the complex formation by comparing the shifted band (pixel intensity) of the reaction with competitor to the reaction without the competitor.

Our data show that sequences of 28 base pairs (lacking 3 bases at the 5′ end or 3′ end of the target site; referred to as 28 bp-u and 28 bp-d, respectively) showed a significantly reduced binding to PRDM9^Cst^ (*p* value <0.0001; generalized least squares model considering non-homogeneous variances and auto-correlation) compared to DNA fragments 31 bp or longer. The missing nucleotides were predicted to contact ZnF2 and ZnF11, which do not confer a specific binding according to our chimera data; thus, these two ZnFs are involved in non-specific contacts or engage in interactions with DNA-phosphate groups. In comparison, the 31 bp and the 34 bp long sequences (contacting ZnFs2–11 and ZnFs1–11, respectively) show both very similar binding behaviors, suggesting that the first degenerate ZnF of the PRDM9^Cst^ array (CH2 type) is not necessary for the PRDM9-DNA interaction and likely does not bind DNA. Interestingly, the 39 bp fragment showed a stronger binding to PRDM9^Cst^ than both the 31 and 34 bp fragment. Binding increased even further for the 75 bp fragment, which showed a significant difference compared to the 34 or the 31 bp fragment (*p* value <0.0001; generalized least squares model considering non-homogeneous variances and auto-correlation).

This suggests that DNA targets that contain flanking regions (i.e., nucleotides that are not predicted to be involved in direct contacts with the ZnFs) in some way enhance the PRDM9 binding. Possibly, these flanking DNA sequences facilitate target recognition (e.g., in a model where PRDM9 slides along the DNA backbone until the encounter of a specific sequence, or these additional nucleotides are targets for interactions with the single ZnF of yet unknown function, which is not part of PRDM9’s tandem array, but instead is spaced by ∼100 amino acids and differs in length and composition of the alpha helix and beta sheets from the remaining ZnFs in the array.

## Discussion

Using the switchSENSE technology and EMSAs, we gained first insights into the kinetics between the ZnF domain of PRDM9^Cst^ and the DNA sequence found in the mouse recombination hotspot *Hlx1* ^B6^. Despite the very different binding conditions, the estimated equilibrium dissociation constants were comparable between the two used methods suggesting that both methods reflect the in vitro binding behavior of the PRDM9^Cst^-ZnF to DNA. Similar binding affinities within the nanomolar range (*K*_D_ ∼ 30 nM) were also reported in a recent in vitro study of a subset of the human PRDM9-ZnF array (Patel et al., 2016). However, it was also shown that reaction conditions (e.g., salt concentration (Patel et al., 2016)) have a strong effect on the *K*_D,_ as we also observed with the addition of polydIdC; thus, in vitro *K*_D_ values might be different to the affinity in the cellular environment.

Nevertheless, our experiments also show that regardless of the reaction conditions used, PRDM9 forms a highly stable, long-lived complex with specific DNA targets within a hotspot. This is the first report on dissociation kinetics of the PRDM9 ZnF with dissociation halftimes of ∼9–17 h. The formation of a highly stable and long-lived complex was observed in all switchSENSE measurements and was corroborated qualitatively with an independent EMSA experiment.

An important structural element found in most C2H2-type ZnFs domains connecting two neighboring ZnFs is an amino acid sequence known as the threonine, glycine, glutamate, lysine, and proline (TGEKP) linker, located between the last histidine of one ZnF and the first conserved aromatic amino acid of the next (Laity et al., 2001, Wolfe et al., 2000). It acts as a spacer between the ZnF along the DNA (Wolfe et al., 2000) and is highly conserved in PRDM9 across divergent species, such as mice and humans. These TGEKP linkers play an important role in the DNA-protein complex stability of ZnF proteins. Based on NMR chemical shift data, Laity et al. showed that the ZnF-protein TFIIIA undergoes a conformational change in the TGEKP linkers during the transition of a non-specific binding mode to a sequence-specific interaction (Laity et al., 2000). During non-specific binding, the TGEKP linkers are rather flexible, enabling the search for its target sequence motifs. Once sequence-specific interactions have occurred, the ZnF repeats are brought into their correct relative orientations and the TGEKP linkers undergo a conformational change, which can be thought of as a “snap lock” that clicks the ZnFs into place in the major groove of the DNA, thereby stabilizing the ZnF-DNA complex mediated by hydrogen bonds and van der Waals forces (Laity et al., 2000). A similar process might be occurring also in PRDM9, explaining the formation of a highly stable complex between sequence-specific target DNA and the ZnF domain of PRDM9.

Whether all ZnFs in the PRDM9 domain undergo this “snap-lock” conformational change is unknown. Usually, multi-ZnF proteins contact DNA in units of two to three successive ZnFs (Iuchi, 2005). However, our chimera experiments, in which, the replacement of the DNA target site with unspecific sequences either from the 5′ or the 3′ end resulted in reduced binding of PRDM9, suggest that the stability of the PRDM9-DNA complex decreases with the number of ZnFs involved in the specific binding (Fig. 4). But we also showed that a subset of the ZnFs (minimum five ZnFs) already confers binding specificity, albeit with reduced affinity compared to the entire ZnF array. Thus, it also might be possible that a conformational change for all ZnFs in the array is not necessary.

The recognition and specific binding of PRDM9^Cst^ to different sequence subsets might be a unique feature of ZnF domains with many ZnFs, like the one found in PRDM9 with 10 or more ZnFs. Interestingly, DNA motifs enriched at recombination hotspots observed in available high-resolution maps also represent only a subset of the sequence recognized by the complete PRDM9-ZnF array. The order and identity of the ZnF determine which motif is enriched. Motifs specific for the murine PRDM9^9R^ (B10.S-H2t4/(9R)/J strain) or PRDM9^Dom2^ (C57BL/6J strain) (Brick et al., 2012, Walker et al., 2015) show a preference for the N-terminal region, while motifs for the murine PRDM9^13R^ (B10.F-H2pb1/(13R) J strain) (Brick et al., 2012) and human PRDM9^A^ (Myers et al., 2010) variants have a preference for the C-terminal region of the ZnF array. Thus, it is possible that only the motif recognized by the slightly more specific ZnF subset is enriched at recombination hotspots.

Not all PRDM9-ZnFs confer binding specificity, but also, interactions between amino acids and the DNA-phosphate backbone are relevant in the complex formation or stability (Patel et al., 2016, Billings et al., 2013). Our data suggest that different sequences contacting ZnF1, ZnF2, ZnF10, and ZnF11 of PRDM9^Cst^ do not change the binding affinity. This was also observed for PRDM9^Cst^-ZnFs10 and ZnF11 with single nucleotide substitutions in the target site (Billings et al., 2013) and for ZnFs1 and ZnF12 in the analysis of sequence motifs preferentially bound by murine PRDM9^B6^ (=PRDM9^Dom2^) (Walker et al., 2015). This is also congruent with the comparison of different PRDM9 variants in several primate species, which showed that ZnFs located at the amino- or carboxy-terminal ends are more conserved (Schwartz et al. 2014), suggesting that these fingers might have a more universal role and contribute little to the binding of specific sequences.

In our EMSA data, the PRDM9-ZnF domain showed a gradually increased binding for longer DNA sequences (e.g., 75 vs. 39 and 34 bp, respectively), an unexpected result given that the 11 fingers of the PRDM9^Cst^-ZnF bind 34 bp. Thus, our data suggest that more nucleotides are used for the interaction than required by the number of ZnFs. There is evidence from cell-line and immunoprecipitation experiments that PRDM9 forms a multimer (Baker et al., 2015). Whether the multimerization also occurs only with the ZnF domain is not known, but if this is the case, a ZnF domain formed by several units might also explain our observations that the interaction with longer DNA sequences is important for the binding stability of the complex. As for the functional importance of this phenomenon, it has been proposed for other DNA-binding proteins that the accelerated target localization happens via a one-dimensional (1D) search mode during which the protein slides along the DNA (reviewed in von Hippel and Berg (1989)). This 1D diffusion, performed as a (1) sliding and (2) intersegmental transfer, can be viewed as a random walk while the protein is in the non-specifically bound state (Berg et al. (1981) and von Hippel and Berg (1989) and also reviewed in Halford and Marko (2004), Mirny et al. (2009), and Zandarashvili et al. (2012)). This type of interaction has also been described for the transcription factor Egr-1, which uses only two of its three ZnF domains during the rapid 1D-search mode and then undergoes a conformational transition in the recognition mode, where all three ZnF domains confer DNA binding (Zandarashvili et al., 2012). Based on our data, it is conceivable that the ZnF domain of PRDM9 also initially scans the DNA by sliding along the non-specifically bound DNA coupled with intersegmental transfer between nucleosomes. A specific target encounter matching the ZnF domain could then induce a conformational change of the ZnF domain via a snap-lock action of the TGEKP linkers, which leads to the formation of a highly stable PRDM9-DNA complex with a half-life of many hours.

Whether the highly stable, long-lived complex has a biological relevance is not known. However, the slow dissociation of PRDM9 from DNA would allow the PRDM9-DNA interaction to persist all the way from the first target recognition until the encounter and activation of the recombination initiation machinery that introduces DSBs. In mice spermatocytes, PRDM9 is expressed from pre-leptotene to mid-zygotene, a period of roughly 48 h (Sun et al., 2015). During these stages of meiosis, the chromatin undergoes substantial changes, starting from a rather diffuse interphase conformation, followed by gradual condensation of chromosomes during the leptotene and zygotene stages, during which also the lateral and axial elements of the synaptonemal complex form, until full synapsis of the chromosomes is reached in the pachytene stage. Given that PRDM9 is already expressed before the start of prophase I (pre-leptotene), it is conceivable that it binds when the genomic DNA is in a fairly open stage during pre-leptotene and stays bound during the entire process of leptonemal loop-axis formation.

One constraint of our kinetic data is that we assessed the binding of PRDM9 to naked DNA (not nucleosomal DNA). Hence, the association and dissociation rates under physiological conditions could differ from our in vitro results. It is not known, whether PRDM9 can bind nucleosomal DNA or whether it requires the help of chromatin remodeling factors that remove the nucleosomes to expose a strand of naked DNA to PRDM9. Studies with engineered transcription factors indicate that the accessibility of the DNA is impaired by the packaging of DNA within nucleosomes (Collingwood et al., 1999). Furthermore, it has been shown recently that the tightness of nucleosomal packaging (i.e., whether the chromatin comprises an open or closed conformation) affects hotspot activation by PRDM9 in mice (Walker et al., 2015). A comparison of the hotspot usage with the chromatin state at the hotspot in B6 mice, determined by DMC1-ChIP-Seq data (Brick et al., 2012) and H3K4me3-ChIP-Seq (Baker et al., 2014), respectively, showed that hotspot usage is increased in actively transcribed genes and decreased in closed chromatin (H3K9me2/me3 or constant lamina associated domains—cLADs) (Walker et al., 2015). However, high affinity targets of PRDM9 (determined by in vitro Affinity-Seq) correlated highly with hotspot usage regardless of the initial chromatin state (Walker et al., 2015). It is yet unclear if and how PRDM9 gets access to the closed chromatic regions. One possibility is that it accesses only open chromatin to begin with. Alternatively, it could be acting in concert with chromatin remodeling factors that displace nucleosomes in an ATP-dependent manner. Another possibility is that it gets access to these regions by spontaneous exposure of nucleosomal DNA by the partial unwrapping of DNA and then remains bound at loci with high affinity sequences, thereby allowing passive access to otherwise hidden target sites (Li et al., 2005, Li and Widom, 2004). Furthermore, it has been suggested that site-specific DNA-binding proteins may recruit chromatin remodeling complexes, once they gained access to a previously buried DNA sequence, which subsequently can move or disassemble that nucleosome, thereby allowing a tighter interaction with the site-specific binding protein (Li and Widom, 2004). These models go in line with recent reports of mammalian recombination hotspots, which exhibit nucleosomal depleted regions (NDR) around predicted PRDM9 binding motifs at the hotspot center (Baker et al., 2014, Lange et al., 2016).

So far, the molecular mechanism of how PRDM9 specifies hotspots is not fully understood. H3K4me3 is necessary for the formation of DSBs during meiosis (Acquaviva et al., 2012, Sommermeyer et al., 2013) and it has been demonstrated that PRDM9 predominantly marks H3K4me3 by its PR/SET domain next to its binding site targeted for DSB (Baker et al., 2014, Brick et al., 2012, Grey et al., 2011). However, an H3K4me3 mark is not sufficient to initiate recombination, and it has been demonstrated that PRDM9 directs away DSBs from H3K4me3 promoter regions (Brick et al., 2012). The recruitment of the recombination machinery to specific DNA regions is quite complex and also depends on larger structural chromosomal components. During the chromatin compaction occurring during prophase I, only a sequence located in the loop during the loop-axis formation in leptotene becomes a DSB target. A key aspect of DSB formation is that the NDR in the axis gets tethered by components placed on the axis (Blat et al., 2002, Ito et al., 2014, Acquaviva et al., 2012, Sommermeyer et al., 2013). The role that PRDM9 plays in this process has not been completely elucidated, but recent evidence has shown that PRDM9 is necessary to tether the DNA in the loop to the axis via helper proteins bound to the KRAB domain (Parvanov et al., 2016). How a long-lived PRDM9-DNA complex plays a role in the initiation of recombination is open for debate, but it could be possible that the constant activity of the PR/SET domain or other epigenetic modifiers of a long-lived complex prevents a target to be packed and hidden in the axial structure. Alternatively, PRDM9 could actively drive the placement of NDR/H3K4me3 chromatin regions in a loop during the highly dynamic chromatin condensation processes occurring in prophase I. Finally, in light of the recent evidence about the role of PRDM9 in recruiting the recombination initiation machinery (Parvanov et al., 2016), a long-lived PRDM9-DNA complex might be important to stabilize the recombination initiation machinery to specific DNA targets such that the PRDM9-DNA complex can be moved from the loop to the axis.

There are still many open questions about the mode of action of PRDM9, but a simple recognition of DNA motifs by PRDM9 does not explain hotspot usage. First, the specific recognition by PRDM9 can be conferred by different subsets of the ZnF domain explaining the plasticity of this protein for a variety of different targets; although, a certain motif is enriched at hotspots by the binding of a predominant ZnF subset. Second, hotspot usage is evidently linked to factors creating NDR regions and, more importantly, the placement of these regions in the loop versus axis during the compaction of the chromatin in meiotic prophase I. The slow dissociation rate of the PRDM9 complex from highly specific sequences might play a role during this process. How PRDM9 specifies hotspots will be better understood with further binding studies investigating chromatin accessibility and PRDM9 recruitment and the role of the other PRDM9 domains and their kinetics.

### Experimental procedures

#### DNA sources

DNA fragments were either produced by PCR using biotinylated or unmodified primers or purchased as synthetic fragments with the necessary modifications. Details are shown in Supplementary_Methods.

#### Cloning and expression of PRDM9^Cst^-ZnF

The coding sequence of PRDM9^Cst^ in form of a pBAD expression construct (kindly provided by the Pektov Lab, Center for Genome Dynamics, the Jackson Laboratory, Bar Harbor, ME 04609, USA) was used to clone the *Prdm9* ^*Cst*^ gene into several different expression systems (pT7-IRES-MycN vector for cell-free in vitro expression, pFB12 vector for insect cell expression, pEYFP-C1 for mammalian cell expression, and pGEX-6P2 vector as an alternative vector system for bacterial expression with a GST tag for enhanced solubility, and finally, the pOPIN-M vector system, which can be used for bacterial, mammalian, and insect cell expression and contains the MBP for enhanced solubility). We tested the most suitable system to express PRDM9^Cst^ (data not shown) and finally chose the pOPIN vector system in combination with a specific *Escherichia* *coli* expression strain (see Supplementary_Methods “Recombinant expression of PRDM9^Cst^ in bacterial cells and lysate preparation”) that gave the best yields of soluble recombinant PRDM9. A detailed description of the cloning processes is described in the Supplementary_Methods, with details on (a) the cloning of PRDM9^Cst^-ZnF (encoded by the exon10) in the pOPIN-M vector using the Gibson Assembly™ cloning kit (NEB), (b) the excision of YFP from the pOPIN-M construct, (c) the cloning of PRDM9^Cst^ (full-length) and PRDM9^Cst^-ZnF construct in the in vitro expression vector pT7-IRES-MycN, and (d) the introduction of a His-YFP-tag into the pT7-IRES-MycN constructs. Also, a detailed description on protein lysate preparation can be found in the Supplementary_Methods. In summary, we used the following constructs of *Prdm9* ^Cst^Exon10 (ZnF domain) or *Prdm9* ^Cst^ full length including several tags, such as a His-tag, the maltose binding protein (MBP), or eYFP (for further details see Supplementary_Methods):

1. His-MBP-eYFP-PRDM9^Cst^Exon10 in pOPIN-M vector (bacterial expression)
2. His-MBP- PRDM9^Cst^Exon10 in pOPIN-M vector (bacterial expression)
3. His-eYFP-PRDM9^Cst^ (full-length) in pT7-IRES-MycN vector (in vitro expression system)
4. His-eYFP-PRDM9^Cst^Exon10 in pT7-IRES-MycN vector (in vitro expression system)
5. PRDM9^Cst^Exon10 in pT7-IRES-MycN vector (in vitro expression system)

#### Electrophoretic mobility shift assays

The EMSA reactions and incubation times varied depending on the experiment but followed the general protocol outlined below. Details of each EMSA reaction setup are described in the Supplementary_Methods: (a) EMSA protein titrations, (b) EMSA competition assay, (c) EMSA experiments with chimera fragments, (d) EMSA simultaneous hot and cold DNA competition assay, and (e) EMSA time course.

##### General EMSA protocol

Electrophoresis of 5% polyacrylamide gels was run in 0.5× TBE buffer (44.5 mM Tris base, 44.5 mM boric acid, 1 mM EDTA, pH 8.0) at 100 V for 30 min before loading the EMSA reaction. The EMSA reactions were supplemented with 4 μl of 6× EMSA loading dye (15% glycerol, 0.03% bromophenol blue, 0.03% xylene cyanol FF, 44.5 mM Tris base, 44.5 mM boric acid, 1 mM EDTA, pH 8.0) and loaded onto the polyacrylamide gels. The gels were run 45 min at 100 V followed by the electrophoretic transfer to a Zeta-Probe nylon membrane (Bio-Rad) at 100 V (constant voltage) for 80 min. Then, the DNA was crosslinked to the nylon membrane using an UV-crosslinker (CX-2000, UVP) at 600 mJ/cm^2^. Afterwards, unspecific binding sites on the membrane were blocked using 1% *w*/*v* (weight per volume) casein (Hammarsten grade, AppliChem) in 1× TBS buffer (25 mM Tris base, 137 mM NaCl, 2.7 mM KCl, pH 7.4) by a 15 min incubation at ∼22 °C, shaking. For the detection of biotinylated DNA, the membrane was incubated for 15 min, shaking at ∼22 °C, in Pierce Streptavidin-Horseradish Peroxidase Conjugate (Thermo Scientific) diluted in blocking buffer (1% *w*/*v* casein (Hammarsten grade, AppliChem) in 1× TBS buffer) to a concentration of 33.35 μg/ml. Next, the membrane was washed 4× for 5 min at 22 °C in a shaker with a wash buffer (300 mM Tris base, 200 mM NaCl, 0.5% SDS, pH 8) and equilibrated for chemiluminescent detection in 300 mM Tris, pH 8 for another 5 min at 22 °C, shaking. Finally, the membrane was carefully transferred to a paper towel, using forceps, removing residual liquid from the membrane edges before addition of Super Signal West Femto Maximum Sensitivity Substrate (Thermo Fisher) mixed in a 1:1 ratio. The chemiluminescent reaction was allowed to take place for 5 min, then the results were obtained by using the ChemiDoc™ MP imager (Bio-Rad) with the blot settings “Chemi Hi Sensitivity.”

##### Image analysis

Image analysis was performed using the Image Lab software (Bio-Rad). The lanes and bands were defined manually then the pixel intensities and values for fraction bound (%) were quantified and analyzed further using OriginPro8.5 (Origin Lab).

##### Statistical analysis

We tested differences in binding trends (Fig. 5) with a generalized least square model using a likelihood ratio test that takes non-homogeneous variances and auto-correlation into account and adjusted with a Bonferroni correction. A detailed description of the analysis and results can be found in the Supplementary_Statisitcal_Analysis.

#### switchSENSE measurements

##### Protein lysate, DNA, and buffers

For the switchSENSE measurements, recombinant PRDM9^Cst^-ZnF was produced using the His-MBP-PRDM9^Cst^ pOPIN-M construct-without YFP (see cloning procedure in Supplementary_Methods) expressed in *E. coli* Rosetta™2(DE3) pLacI. The PRDM9 concentration in the crude lysate (see Lysate preparation for His-MBP-PRDM9^Cst^-ZnF-without YFP in Supplementary_Methods and Supplementary_Fig_S1, panel A, lane 2) was estimated by Capillary Western (Supplementary_Fig_S1, panel B). For the target DNA sequences, we used 48 bp double-stranded synthetic fragments (*Hlx1* ^B6^ and usDNA; Supplementary_Table_S1, panel C, switchSENSE) that carry a thiol modification on the 5′ end and a fluorescent dye on the 3′ end of the forward strand and no modification on the reverse strand. Running and sample buffer were in experiments 2 and 3 (low polydIdC), 10 mM Tris (pH 7.5), 50 mM KCl, 0.05% NP40, and 50 μM ZnCl_2_ and supplemented with 50 ng/μl polydIdC in experiment 1 (high polydIdC).

##### Instrument, chip and DNA layer preparation, regeneration process, and flow rates

All switchSENSE measurements were performed on a DRX2400 instrument using custom made sensor chips (both Dynamic Biosensors GmbH; Planegg, Germany). On the respective sensor chip, different sensor spots in one flow channel were either functionalized with single-stranded *Hlx1* ^B6^ or usDNA by a sulfur-gold bond at the 5′ end of the DNA. For the detection of the switching motion, the DNA molecules were modified with a fluorescent dye at the 3′ end. The single-stranded DNA probes were hybridized to the respective complementary sequence on chip at 45 °C to resolve potential secondary structures. The hybridizations of usDNA and *Hlx1* ^B6^ were carried out separately by sequential incubation with 200 nM usDNA reverse followed by 200 nM *Hlx1* ^B6^ reverse. In each case, successful hybridization was monitored by real-time observation of the switching amplitude on either an usDNA or *Hlx1* ^B6^ modified electrode. After functionalization with the respective DNA, one sensor spot contains about one million DNA molecules. For complete chip regeneration, the electrodes were treated with regeneration solution (Dynamic Biosensors GmbH; Planegg, Germany) to remove the complementary DNA strands and potentially bound proteins. The remaining single-stranded DNA was freshly hybridized as described above. Intermittent kinetic measurements on two differently functionalized sensor spots allowed the parallel determination of the binding kinetics of PRDM9 to both DNA sequences. All association and dissociation experiments were performed at a pump rate of 5 μl/min.

##### Data normalization and analysis

The kinetic data (dynamic response upwards 0–4 μs, which corresponds to the nanolever’s switching speed during the first 4 μs of the upward motion; see more details in (Langer et al., 2013)) were grouped and exported from the switchANALYSIS software (Dynamic Biosensors GmbH) and the association start values of the different concentrations were normalized to 100%. The respective dissociation data were normalized accordingly. The normalized dynamic response was plotted against time using the OriginPro8.5 software (Origin Lab), and binding kinetics were analyzed by single or global exponential fits using the following equations for association and dissociation, respectively. Fit equation for association rate constant (*k*_on_), *y* = *y*0 + *A* * exp (−(*x* − *x*0)*(c*k_on_ + *k*_off_)) and dissociation rate constant (*k*_off_), *y* = *y*0 + A*exp (−(*x* − *x*0)/*t*) with the dependency that *k*_off_ = 1/*t*. Here, *x*0 and *y*0 are the offsets from the respective axis, *A* is the fit amplitude, and *c* is the PRDM9 concentration. The equilibrium dissociation constant (*K*_D_) was then derived by *K*_D_ = *k*_off_/*k*_on_. The error of the equilibrium dissociation constant (Δ*K*_D_) was calculated based on the Gaussian error propagation Δ*K*_D_ = ((1/*k*_on_ * Δ*k*_off_)^2 + (*k*_off_/(*k*_on_^2) * Δ*k*_on_)^2)^(0.5).

## Electronic supplementary material

ESM 1 (PDF 714 kb)

## Abbreviations

PRDM9: PR domain containing protein 9
ZnF: Zinc finger
DSB: Double-strand break
*K*_D_: Equilibrium dissociation constant
nM: Nanomolar
DNA: Deoxyribonucleic acid
CAST: Inbred mouse strain CAST/EiJ
*Hlx1*^B6^: Hotspot DNA sequence
EMSA: Electrophoretic mobility shift assays
usDNA: Unspecific DNA
*k*_off_: Dissociation rate constant
*k*_on_: Association rate constant
polydIdC: Poly(deoxyinosinic-deoxycytidylic) acid
