# Supplementary material for: The long zinc finger domain of PRDM9 forms a highly stable and long-lived complex with its DNA recognition sequence
Source: Chromosome Res. 2017 Feb 2;25(2):155–72. doi: 10.1007/s10577-017-9552-1 (PMC5440498; doi:10.1007/s10577-017-9552-1)
Supplement: Supplementary file 1 — (PDF 714 kb) [file 10577_2017_9552_MOESM1_ESM.pdf]

SUPPLEMENTARY FILES

PR domain containing protein 9 (PRDM9) forms a highly stable and long-lived complex with its DNA recognition sequence

Yasmin Striedner[1]*, Theresa Schwarz[1]*, Thomas Welte[2], Andreas Futschik[3], Ulrich Rant[2], and Irene Tiemann-Boege[1]

Author affiliations:

[1]Institute of Biophysics, Johannes Kepler University, 4020, Linz, Austria.

[2]Dynamic Biosensors GmbH, 82152 Planegg, Germany.

[3]Department of Applied Statistics, Johannes Kepler University, 4040, Linz, Austria.

*These authors contributed equally to this work

Table of contents

Supplementary_Figures 3

Supplementary_Fig_S1 3

Supplementary_Fig_S2 4

Supplementary_Fig_S3 6

Supplementary_Fig_S4 7

Supplementary_Tables.. 8

Supplementary_Table_S1.. 8

Supplementary_Table_S2..11

Supplementary_Methods..13

Cloning 13

a.) Generation of a His-MBP-YFP-PRDM9^{Cst}-ZnF pOPIN construct 13

b.) Generation of a His-MBP-PRDM9^{Cst} pOPIN-M construct-without YFP 14

c.) Generation of a PRDM9^{Cst} (full-length) and PRDM9^{Cst}-ZnF construct in the expression vector pT7-IRES-MycN..15

d.) Introduction of His-YFP in the vector construct..16

pOPIN-,pGEX-, and pT7-IRES-MycN- expression constructs 17

Recombinant expression of PRDM9^{Cst} in bacterial cells and lysate preparation 26

Cell-free *in vitro* expression of His-YFP-PRDM9^{Cst} and (His-YFP)-PRDM9^{Cst}-ZnF..27

PRDM9 quantification (Capillary Western) and verification of protein integrity (SDS-PAGE) 27

EMSA 28
a.) EMSA – protein titrations (K_D determination) 28
b.) EMSA – competition assay) 29
c.) EMSA – Experiments with Chimera fragments (Figure 4) 31
d.) EMSA – Simultaneous hot and cold DNA competition assays (Figure 5) 31
e.) EMSA – Time course (Supplementary_Fig_S4) 32
Supplementary_Statistical_Analysis 34
Statistical Modeling of Binding Footprint Data 34
References 36

Supplementary_Figures

Supplementary_Fig_S1

SDS-PAGE & Capillary Western Data

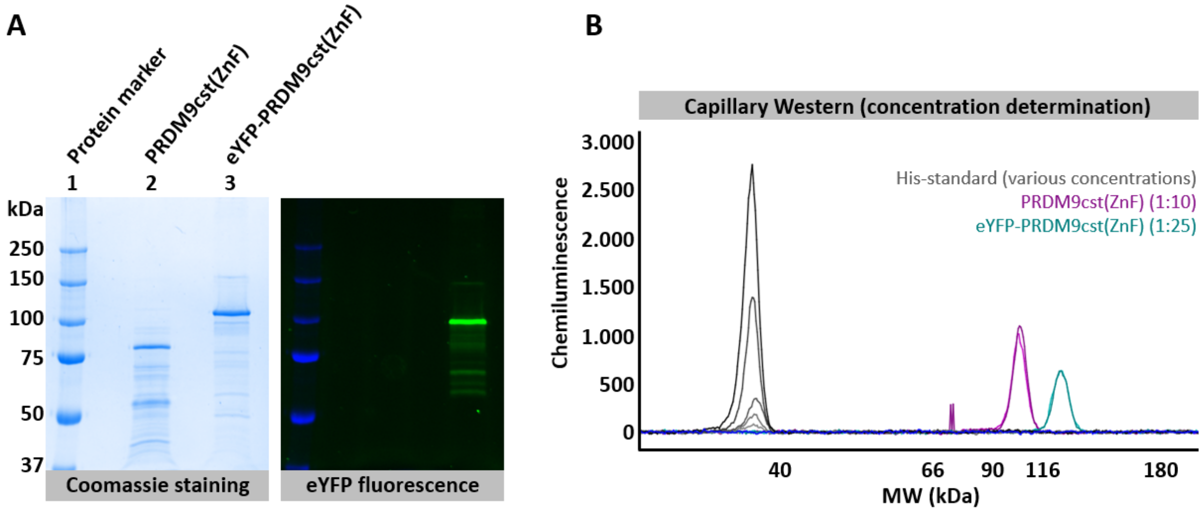

Supplementary_Fig_S1: PRDM9 protein lysates and Capillary Western. **(A)** The purity of the protein lysates, and the molecular weight of PRDM9 were assessed via SDS-gel electrophoresis. 2µl of each lysate were mixed with Laemmli-buffer and loaded on an 8% gel. We first imaged the gel at 510nm to monitor the fluorescence of the His-MBP-eYFP-PRDM9^{Cst}-ZnF fusion protein, followed by a Coomassie staining of the gel. The pixel intensities of the bands in the Coomassie-stained gel were measured with the Image Lab software (Bio-Rad). His-MBP-PRDM9^{Cst}-ZnF (lane 2) has an expected molecular weight of 96kDa and a purity of 27%. His-MBP-eYFP-PRDM9^{Cst}-ZnF (lane 3) has an expected molecular weight of 123kDa and a purity of 55%. **(B)** The PRDM9 concentration in the crude lysates was estimated by Capillary Western based on the binding of an anti-His antibody to His-tagged PRDM9 in comparison to a His-tagged standard protein of known concentration quantified by a chemiluminescence reaction. Appropriate dilutions of the samples were prepared for both the His-standard (125µg/ml – 25µg/ml) and the PRDM9 samples (1:5-1:25, depending on the sample). The data were analyzed using the Compass software from Protein Simple. His-MBP-PRDM9^{Cst}-ZnF (lane 2) showed a concentration of 22.84µM and His-MBP-eYFP-PRDM9^{Cst}-ZnF (lane 3) had a concentration of 49.31µM.

Supplementary_Fig_S2

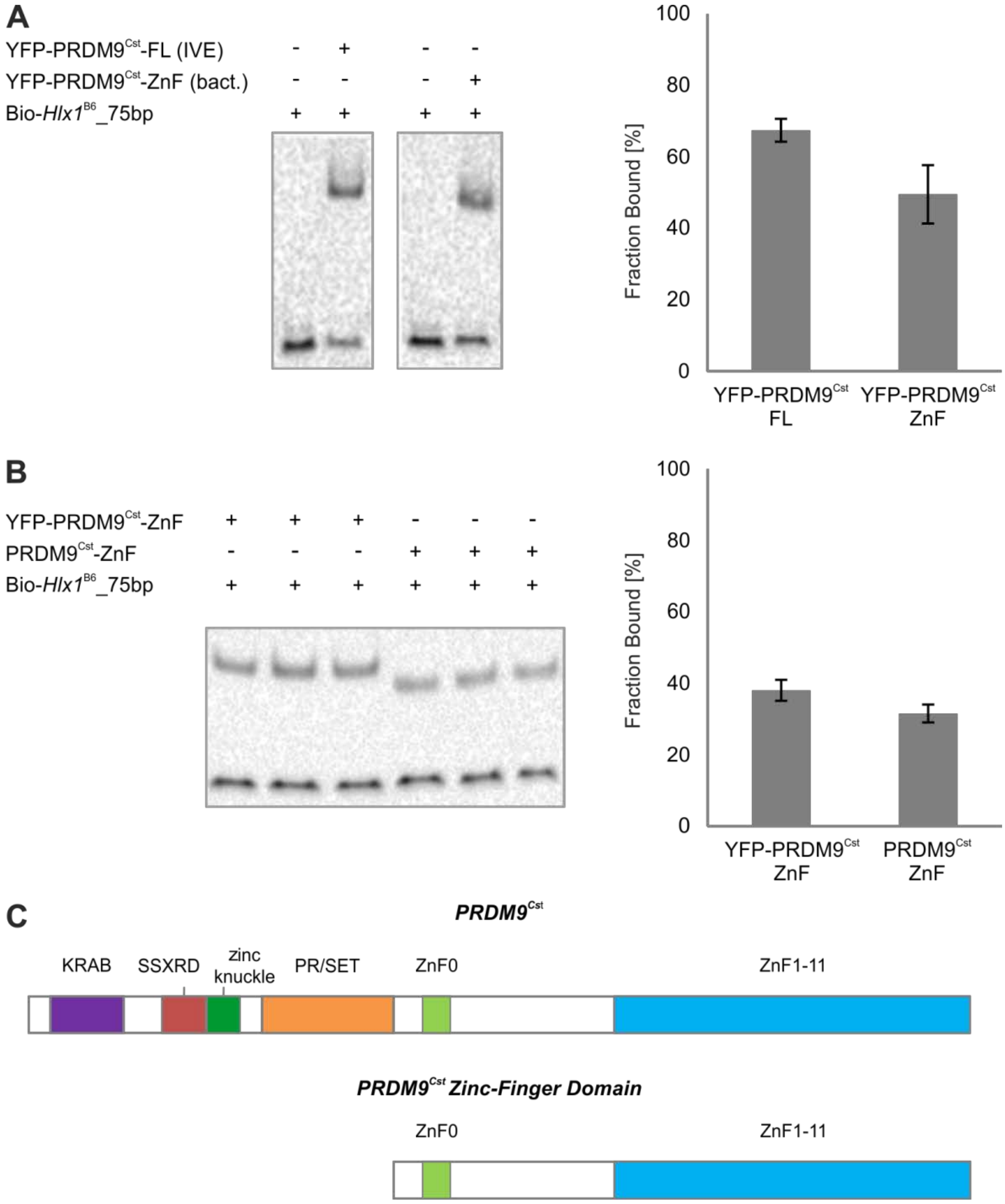

Supplementary_Fig_S2: PRDM9-DNA binding with EMSA using different lysates and domains. **(A)** Electrophoretic Mobility Shift Assay (EMSA) experiments of the full-length PRDM9 containing a YFP-tag (YFP-PRDM9^{Cst}-FL, 127kDa) obtained by *in vitro* expression (IVE) compared with a ZnF-domain (eYFP-PRDM9^{Cst}-ZnF, 123kDa; containing an additional MBP-tag), obtained from a bacterial whole-cell lysate (bact.) incubated with a 75bp DNA fragment of the murine *Hlx1*^{B6} hotspot. Approximately 364nM PRDM9^{Cst}-FL and 247nM PRDM9^{Cst} -ZnF were incubated with 15nM biotin-labeled DNA for 1hr at room temperature or overnight at 4°C. Binding reactions were separated on a native polyacrylamide gel for 55 minutes at 100V. Experiments were performed in triplicates and the average fractions of DNA bound were calculated and plotted in a histogram that shows a similar binding between the full-length and ZnF-domain PRDM9^{Cst}. **(B)** Assessment of the complex of an *in vitro* expressed YFP-labeled and unlabeled murine PRDM9^{Cst}-ZnF domain (YFP-PRDM9^{Cst}-ZnF,

83kDa; PRDM9^{Cst}-ZnF, 55kDa) to the 75bp *Hlx1^{B6}* hotspot DNA using EMSA. Approximately, 0.5µM of *in vitro* expressed crude protein lysates were incubated with 5nM biotin-labeled DNA for 20 minutes at room temperature. The migration distance of the complex (shifted band) can be explained by the differently-sized fusion-proteins. The average fraction bound of the triplicate experiments was calculated and plotted in a histogram showing that additional domains (e.g. YFP) do not influence the binding. The binding patterns observed for the *in vitro* expression system (YFP-PRDM9^{Cst}-ZnF and PRDM9^{Cst}-ZnF) are comparable to the results for YFP-PRDM9^{Cst}-ZnF obtained from bacterial extracts (see panel A; and Figure_S4). **(C)** Schematics of the different PRDM9 constructs used in this study showing the domains of the full-length murine and ZnF domain of PRDM9^{Cst}, respectively (modified from (Baudat et al., 2013)). Note that the PRDM9 constructs contained several different tags (such as the His-tag, MBP, eYFP, cMyc-tag, or Avi-tag) at the N-terminal end (see also Supplementary Methods).

Supplementary_Fig_S3

Kinetic data of the PRDM9^{Cst}-ZnF binding to the Hlx1^{B6} DNA

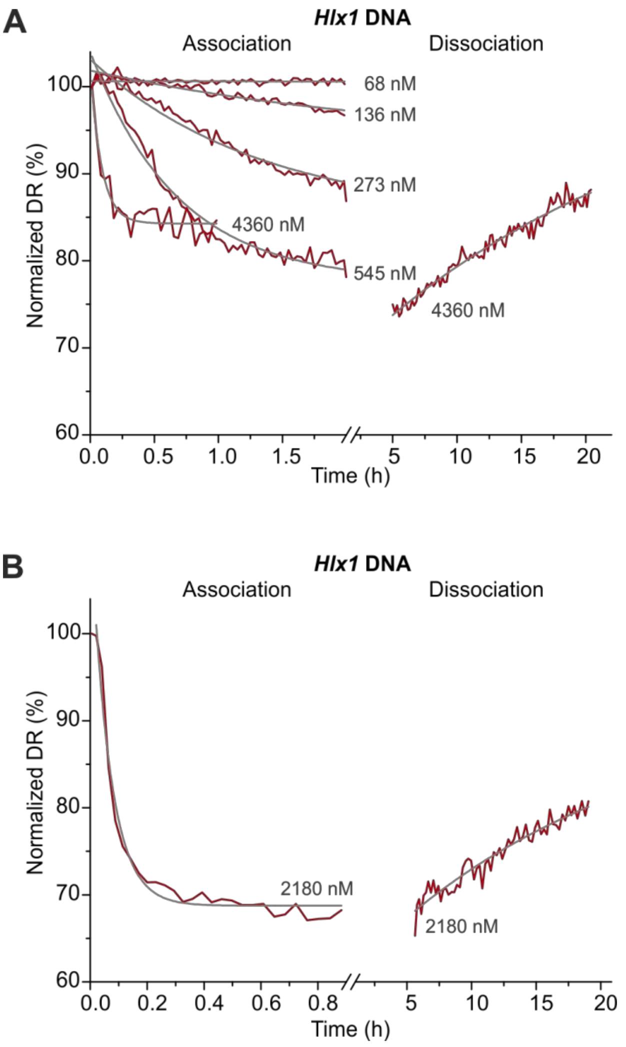

Supplementary_Fig_S3:
Binding kinetics of the PRDM9^{Cst}-ZnF domain to Hlx1^{B6} with titrated (low) polydIdC conditions performed in two different experiments.
Real-time association of the PRDM9^{Cst}-ZnF domain to a 48bp long*Hlx1*^{B6} sequence, shown as changes of the normalized Dynamic Response (i.e. nanolever switching speed). Different (68nM - 4360nM) or just one concentration (2180nM) of PRDM9 was incubated for 1-2 hours with the target DNA sequence *Hlx* immobilized on a microchip. PolydIdC was diluted accordingly, starting at ~9.4ng/µl for the 4360nM concentration step. The last incubation step (with the highest PRDM9 concentration) was followed by a long dissociation measurement of ~ 15 hours. The grey lines show the global or individual exponential fits for the association rate constant (k_{on}) or the dissociation rate constant (k_{off}), respectively. Binding kinetics (k_{on}, k_{off}, and K_D) derived from this data are shown in Table 1. The jump observed in the panel A for the highest PRDM9 concentration potentiallycould be due to liquid handling and air bubble separation.

Supplementary_Fig_S4

EMSA – time course

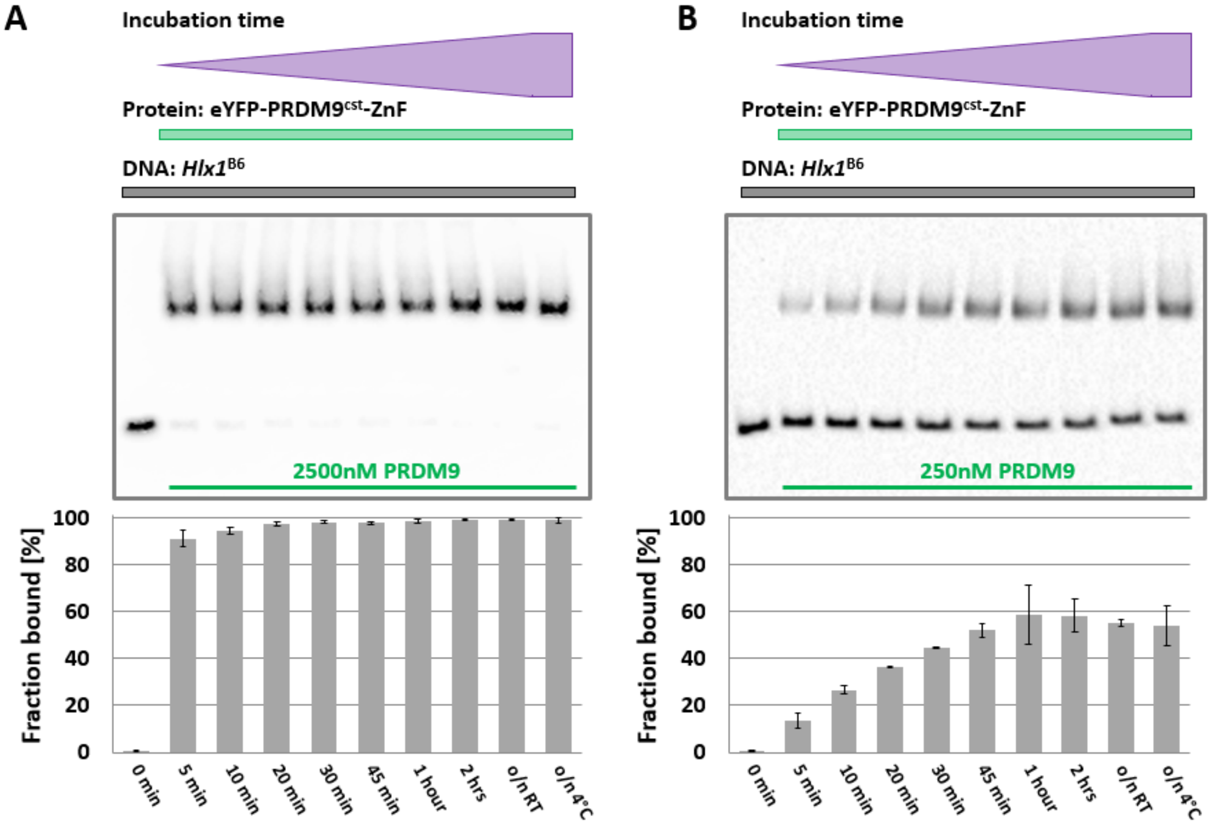

Supplementary_Fig_S4: Assessment of PRDM9-DNA complex formation with EMSA (time course: variation of incubation time). The time to reach an equilibrium state for the PRDM9-DNA binding varies in dependence of the protein and ligand concentration. We used EMSA to test when an equilibrium of the PRDM9-DNA binding was reached for the following conditions: **(A)** 5nM DNA + 2.5µM PRDM9 and **(B)** 15nM DNA and 250nM PRDM9. The error bars represent the standard deviation of two independent experiments. These results were used as the basis for the experimental setup of the EMSAs shown in Figures 2, 4, and5. Note that the equilibrium of complex formation lies at >90% for the high PRDM9 concentration and is reached already after 20min, while for the low PRMD9 concentration the equilibrium of complex formation lies at <60% and is reached after ~45min.

Supplementary_Tables

Supplementary_Table_S1

Sequences of primers and synthetic DNA fragments

A

Primer Name	Primer Sequence 5' to 3' direction	amplified fragment	Usage
Bio-Hlx1-75bp_F	**Bio**-GTGGGAGGAGATGGTGGGTG	Bio-Hlx1 75bp	EMSA: Chimera experiments
Bio-Hlx1-75bp_R	**Bio**-CCCATGGTTAGTGGAATGCGTAAAG		
Bio-negctrl_F	**Bio**-AACCCTGAGCTGCCTAAAGGTC	neg. control DNA	
Bio-negctrl_R	**Bio**-TGTGCCTCTGGTATTCACAGCAG		
Hlx1 31bp_F	AGTGTGCAGACTTGGACCC	Hlx1 31bp	
Hlx1 31bp_R	AAAGAAAGGGCAGGGTCCA		
Chimnt 1-31_F	CTGCCTAAAGGTCAAGTGTGCAGACTT	inside Chimnt 1-31	
Chimnt 1-31_R	GGTATTCACAGCAGAAAGAAAGGGCAG		
Chimnt 6-31_F	TGCCTAAAGGTCAGAATCGCAGACTTGGACCCT	inside Chimnt 6-31	
Chimnt 6-31_R	TATTCACAGCAGAAAGAAAGGGCAGGGTCCAAGTCTG		
Chimnt 11-31_F	TGCCTAAAGGTCAGAATCCACCACTTGGACCCT	inside Chimnt 11-31	
Chimnt 11-31_R	TATTCACAGCAGAAAGAAAGGGCAGGGTCCAAGTGGT		
Chimnt 16-31_F	TGCCTAAAGGTCAGAATCCACCATAGTGACCCT	inside Chimnt 16-31	
Chimnt 16-31_R	TATTCACAGCAGAAAGAAAGGGCAGGGTCACTATGGTG		
Chimnt 21-31_F	TGCCTAAAGGTCAGAATCCACCATAGTGAGAGA	inside Chimnt 21-31	
Chimnt 21-31_R	TATTCACAGCAGAAAGAAAGGGCTCTCTCACTATGGTG		
Chimnt 1-26_F	TGCCTAAAGGTCAAGTGTGCAGACTTGGACCCTG	inside Chimnt 1-26	EMSA: Chimera experiments
Chimnt 1-26_R	TATTCACAGCAGCAGCAAAGGGCAGGGTCCAAGTCT		
Chimnt 1-26_F	TGCCTAAAGGTCAAGTGTGCAGACTTGGACCCTG	inside Chimnt 1-21	

Chimnt 1-21_R	TATTCACAGCAGCAGCACTTGCCAGGGTCCAAGTCT		
Chimnt 1-16_F	TGCCTAAAGGTCAAGTGTGCAGACTTGGAAGATA	inside Chimnt 1-16	
Chimnt 1-16_R	TATTCACAGCAGCAGCACTTGCTATCTTCCAAGTCT		
Chimnt 1-11_F	TGCCTAAAGGTCAAGTGTGCAGACGTGAGAGATA	inside Chimnt 1-11	
Chimnt 1-11_R	TATTCACAGCAGCAGCACTTGCTATCTCTCACGTCT		
Chimnt 6-10_F	TGCCTAAAGGTCAGAATCGCAGAAGTGAGAGATA	inside Chimnt 6-10	
Chimnt 6-10_R	TATTCACAGCAGCAGCACTTGCTATCTCTCACTTCT		
Chimnt 6-15_F	TGCCTAAAGGTCAGAATCGCAGACTTGGGAGATA	inside Chimnt 6-15	
Chimnt 6-15_R	TATTCACAGCAGCAGCACTTGCTATCTCCCAAGTCT		
Chimnt 6-20_F	TGCCTAAAGGTCAGAATCGCAGACTTGGACCCTA	inside Chimnt 6-20	
Chimnt 6-20_R	TATTCACAGCAGCAGCACTTGCTAGGGTCCAAGTCT		
Chimnt 6-25_F	TGCCTAAAGGTCAGAATCGCAGACTTGGACCCTG	inside Chimnt 6-25	
Chimnt 6-25_R	TATTCACAGCAGCAGCACAGGGCAGGGTCCAAGTCT		
predicted_F	TGGTGGGTGAATCCTCATCTACTGCATCAGC	inside Chim predicted	
predicted_R	GGAATGCGTAAAGTAGAAGATGCAGATGCTGA		
chimnegctrl_F	TGGTGGGTGAATGAATCCACCATAGT	inside Chimnegcntrl	
chimnegctrl_R	TGGAATGCGTAAAGAGCACTTGCTATC		

B

Primer Name	Primer Sequence 5' to 3' direction	Usage	Comment
mP9_fwd_XhoIAvi	tatctaCTCGAG**atgtccggcctgaacgacatcttcgaggctcagaaaatcgaatggcacgaa**ATGAACACCAACAAGCTGGAA	RE-based cloning	Avi-tag shown as bold letters
mP9_rvs_BamHI	cgtcgtGGATCC**TTA**CTTCTCTCTTGTATGTGGCCTCTG	RE-based cloning	STOP codon shown as bold letters
mP9Ex10_F_XhoAv	tatctaCTCGAGatgtccggcctgaacgacatcttcgaggctcagaaaatcgaatggcacgaaCTAAGGACAGAAATTCATCCT	RE-based cloning	
eGFP_fwd_XhoI	tatctaCTCGAGcatcatcatcatcatcatATGGTGAGCAAGGGCGAG	RE-based cloning	
eGFP_rvs_XhoI	tgtcgtCTCGAGCTTGTACAGCTCGTCCATGCC	RE-based cloning	
IF_opin-GFP_2_F	ggaagttctgtttcagGGTACC**CTGGAAGTTCTGTTCCAGGGGCCC**CTCGAGatggtgagcaagggcgag	In-Fusion cloning	PreScissionTM cleavage site as bold letters
IF_GFP-mprd9_2_R	aatacaagttttctGCGGCCGCCTCGAGcttgtacagctcgtccatgcc	In-Fusion cloning	

IF_gfp-mPRD9_2_F	GGCCGCa**gaaaacttgtatttccagggcGAG**	In-Fusion cloning	Bases aligning to the pGEX vector template are highlighted as bold letters
IF_mPR9-opin_2_R	ggtgatgtttaaactggtctagaAAGCT**TGCGGCCGCTCATTACTTCTC**	In-Fusion cloning	Bases aligning to the pGEX vector template are highlighted as bold letters

C

synthetic DNA	DNA Sequence 5' to 3' direction	Usage
*Hlx1*_75bp_F	GTGGGAGGAGATGGTGGGTGAATAGTGTGCAGACTTGGACCCTGCCCTTTCTTTACGCATTCCACTAACCATGGG	EMSA: Simultaneous hot and cold DNA competition assays, these fragments were ordered at Eurofins; Germany
*Hlx1*_75bp_R	CCCATGGTTAGTGGAATGCGTAAAGAAAGGGCAGGGTCCAAGTCTGCACACTATTCACCCACCATCTCCTCCCAC	
*Hlx1*_39bp_F	TGAATAGTGTGCAGACTTGGACCCTGCCCTTTCTTTACG	
*Hlx1*_39bp_R	CGTAAAGAAAGGGCAGGGTCCAAGTCTGCACACTATTCA	
*Hlx1*_34bp_F	AATAGTGTGCAGACTTGGACCCTGCCCTTTCTTT	
*Hlx1*_34bp_R	AAAGAAAGGGCAGGGTCCAAGTCTGCACACTATT	
*Hlx1*_31bp_F	AGTGTGCAGACTTGGACCCTGCCCTTTCTTT	
*Hlx1*_31bp_R	AAAGAAAGGGCAGGGTCCAAGTCTGCACACT	
*Hlx1*_28bp-d_F	AGTGTGCAGACTTGGACCCTGCCCTTTC	
*Hlx1*_28bp-d_R	GAAAGGGCAGGGTCCAAGTCTGCACACT	
*Hlx1*_28bp-u_F	GTGCAGACTTGGACCCTGCCCTTTCTTT	
*Hlx1*_28bp-u_R	AAAGAAAGGGCAGGGTCCAAGTCTGCAC	
Hlx1^{B6}_F	**HSC6**-GATGGTGGGTGAATAGTGTGCAGACTTGGACCCTGCCCTTTCTTTACG-**Dye**	SwitchSENSE measurements, these fragments were ordered at biomers.net GmbH; Germany
Hlx1^{B6}_R	CGTAAAGAAAGGGCAGGGTCCAAGTCTGCACACTATTCACCCACCATC	
usDNA_F	**HSC6**-CTGCCTAAAGGTCAGAATCCACCATAGTGAGAGATAGCAAGTGCTGCT-**Dye**	
usDNA_R	AGCAGCACTTGCTATCTCTCACTATGGTGGATTCTGACCTTTAGGCAG	

Supplementary_Table_S2

PCR cycling conditions

Fragment	PCR round	Primers	Primer conc.	Template	Denaturing temperature and time	Annealing temperature and time	Extension time and cycle number	Product size
Mouse Hlx1(B6)	1	Bio-Hlx1 75bp_F<br>Bio-Hlx1 75bp_R	1µM<br>1µM	1ng/µl B6 gDNA	94°C, 15s	60°C, 15s	10s, 30x	75bp
	2	Bio-Hlx1 75bp_F<br>Bio-Hlx1 75bp_R	50nM<br>50nM	10^{10} copies of 1st PCR product	94°C, 15s	60°C, 15s	10s, 25x	
unspecific DNA	1	Bio-negctrl_F<br>Bio-negctrl_R	1µM<br>1µM	1ng/µl blood 1042 gDNA	94°C, 15s	60°C, 15s	10s, 30x	75bp
	2	Bio-negctrl_F<br>Bio-negctrl_R	50nM<br>50nM	10^{10} copies of 1st PCR product	94°C, 15s	60°C, 15s	10s, 25x	
Chim. nt 1-31	1	Hlx1 31bp_F<br>Hlx1 31bp_R	1µM<br>1µM	-	94°C, 15s	54°C, 15s	5s, 35x	31bp
	2	Chimnt 1-31_F<br>Chimnt 1-31_R	1µM<br>1µM	10^{10} copies of 1st PCR product	94°C, 15s	50°C, 15s	10s, 30x	59bp
	3	Bio-negctrl_F<br>Bio-negctrl_R	200nM<br>200nM	10^{10} copies of 2nd PCR product	94°C, 15s	50°C, 15s	10s, 35x	77bp
	4	Bio-negctrl_F<br>Bio-negctrl_R	50nM<br>50nM	10^{10} copies of 3rd PCR product	94°C, 15s	60°C, 15s	10s, 25x	77bp
Chim. nt 6-31	1	Chimnt 6-31_f<br>Chimnt 6-31_R	1µM<br>1µM	-	94°C, 15s	50°C, 15s	10s, 30x	56bp
	2	Bio-negctrl_F<br>Bio-negctrl_R	1µM<br>1µM	10^{10} copies of 1st PCR product	94°C, 15s	50°C, 15s	10s, 30x	77bp
	3	Bio-negctrl_F<br>Bio-negctrl_R	200nM<br>200nM	10^{10} copies of 2nd PCR product	94°C, 15s	60°C, 15s	10s, 25x	77bp
Chim. nt 11-31	1	Chimnt 11-31_F<br>Chimnt 11-31_R	1µM<br>1µM	-	94°C, 15s	50°C, 15s	10s, 30x	56bp
	2	Bio-negctrl_F<br>Bio-negctrl_R	1µM<br>1µM	10^{10} copies of 1st PCR product	94°C, 15s	50°C, 15s	10s, 30x	77bp
	3	Bio-negctrl_F<br>Bio-negctrl_R	200nM<br>200nM	10^{10} copies of 2nd PCR product	94°C, 15s	60°C, 15s	10s, 25x	77bp
Chim. nt 16-31	1	Chimnt 16-31_F<br>Chimnt 16-31_R	1µM<br>1µM	-	94°C, 15s	50°C, 15s	10s, 30x	56bp
	2	Bio-negctrl_F<br>Bio-negctrl_R	1µM<br>1µM	10^{10} copies of 1st PCR product	94°C, 15s	50°C, 15s	10s, 30x	77bp
	3	Bio-negctrl_F<br>Bio-negctrl_R	200nM<br>200nM	10^{10} copies of 2nd PCR product	94°C, 15s	60°C, 15s	10s, 25x	77bp
Chim. nt 21-31	1	Chimnt 21-31_F<br>Chimnt 21-31_R	1µM<br>1µM	-	94°C, 15s	50°C, 15s	10s, 30x	56bp
	2	Bio-negctrl_F	1µM	10^{10} copies of 1st PCR product	94°C, 15s	50°C, 15s	10s, 30x	77bp

		Bio-negctrl_R	1µM					
	3	Bio-negctrl_F<br>Bio-negctrl_R	200nM<br>200nM	10^{10} copies of 2nd PCR product	94°C, 15s	60°C, 15s	10s, 25x	77bp
Chim. nt 1-26	1	Chimnt 1-26_F<br>Chimnt 1-26_R	200nM<br>200nM	-	94°C, 15s	50°C, 15s	10s, 30x	56bp
	2	Bio-negctrl_F<br>Bio-negctrl_R	1µM<br>1µM	10^{10} copies of 1st PCR product	94°C, 15s	50°C, 15s	10s, 30x	77bp
	3	Bio-negctrl_F<br>Bio-negctrl_R	50nM<br>50nM	10^{10} copies of 2nd PCR product	94°C, 15s	60°C, 15s	10s, 25x	77bp
Chim. nt 1-21	1	Chimnt 1-26_F<br>Chimnt 1-21	200nM<br>200nM	-	94°C, 15s	50°C, 15s	10s, 30x	56bp
	2	Bio-negctrl_F<br>Bio-negctrl_R	50nM<br>50nM	10^{8} copies of 1st PCR product	94°C, 15s	50°C, 15s	10s, 35x	77bp
Chim. nt 1-16	1	Chimnt 1-16_F<br>Chimnt 1-16_R	200nM<br>200nM	-	94°C, 15s	50°C, 15s	10s, 30x	56bp
	2	Bio-negctrl_F<br>Bio-negctrl_R	50nM<br>50nM	10^{8} copies of 1st PCR product	94°C, 15s	50°C, 15s	10s, 35x	77bp
Chim. nt 1-11	1	Chimnt 1-11_F<br>Chimnt 1-11_R	200nM<br>200nM	-	94°C, 15s	50°C, 15s	10s, 30x	56bp
	2	Bio-negctrl_F<br>Bio-negctrl_R	1µM<br>1µM	10^{10} copies of 1st PCR product	94°C, 15s	50°C, 15s	10s, 30x	77bp
	3	Bio-negctrl_F<br>Bio-negctrl_R	50nM<br>50nM	10^{10} copies of 2nd PCR product	94°C, 15s	60°C, 15s	10s, 25x	77bp
Chimnt 6-25	1	Chimnt 6-25_F<br>Chimnt 6-25_R	200nM<br>200nM	-	94°C, 15s	50°C, 15s	10s, 30x	56bp
	2	Bio-negctrl_F<br>Bio-negctrl_R	50nM<br>50nM	10^{8} copies of 1st PCR product	94°C, 15s	50°C, 15s	10s, 35x	77bp
	3	Bio-negctrl_F<br>Bio-negctrl_R	50nM<br>50nM	10^{10} copies of 2nd PCR product	94°C, 15s	60°C, 15s	10s, 25x	77bp
Chimnt 6-20	1	Chimnt 6-20_F<br>Chimnt 6-20_R	200nM<br>200nM	-	94°C, 15s	50°C, 15s	10s, 30x	56bp
	2	Bio-negctrl_F<br>Bio-negctrl_R	50nM<br>50nM	10^{8} copies of 1st PCR product	94°C, 15s	50°C, 15s	10s, 35x	77bp
Chim. nt 6-15	1	Chimnt 6-15_F<br>Chimnt 6-15_R	200nM<br>200nM	-	94°C, 15s	50°C, 15s	10s, 30x	56bp
	2	Bio-negctrl_F<br>Bio-negctrl_R	50nM<br>50nM	10^{10} copies of 1st PCR product	94°C, 15s	50°C, 15s	10s, 25x	77bp
Chimnt 6-10	1	Chimnt 6-10_F<br>Chimnt 6-10_R	200nM<br>200nM	-	94°C, 15s	50°C, 15s	10s, 30x	56bp
	2	Bio-negctrl_F<br>Bio-negctrl_R	50nM<br>50nM	10^{8} copies of 1st PCR product	94°C, 15s	50°C, 15s	10s, 35x	77bp

Supplementary_Methods

Cloning

a.) Generation of a His-MBP-YFP-PRDM9^{Cst}-ZnF pOPIN construct

PCR amplification of the *Prdm9*^{Cst} gene and preparation of the insert for ligation.As first step, the *Prdm9*^{Cst} insert was amplified out of a previously generated *Prdm9*^{Cst}_pGEX construct (for exact sequence see page 14-16) using the primer setIF_gfp-mPRD9_2_F and IF_mPR9-opin_2_R (primer sequences are shown in Supplementary_Table_S1). As polymerase 0.02 U/µl Phusion Hot Start II polymerase (Thermo Scientific) were used in a 50µl reaction in HF-buffer supplemented with 0.2mM dNTPs (Biozym) and 1%DMSO (Thermo Scientific). 10^{10} molecules vector DNA were used as template. The PCR cycle started with an initial heating step of 98°C for 30 seconds (sec), followed by 15 cycles at 98°C for 10sec, 60°C for 15sec, and 72°C for 80sec, followed by 10 cycles at 98°C for 10sec, and 72°C for 95sec, concluded by a final elongation step of 7 minutes (min) at 72°C. The correct length of the amplicon was assessed via gel electrophoresis and the band was excised from a 1%agarose gel and purified using the Wizard SV Gel and PCR Clean-Up System (Promega) according to manufacturer's instructions. The amplicon was eluted in 30µl DNase/RNase-free, double destilled water (ddH_2O) and the DNA concentration was determined using a Nanodrop 2000 instrument (Thermo Scientific).

PCR amplification of the *YFP* gene. The *YFP* gene was amplified using 10^{10} molecules of the pEYFP-N1 vector (Clontech) and the primer setIF_opin-GFP_2_F and IF_GFP-mprd9_2_R (primer sequences are shown in Supplementary_Table_S1). As polymerase 0.02 U/µl Phusion Hot Start II polymerase (Thermo Scientific) were used in a 50µl reaction in HF-buffer supplemented with 0.2mM dNTPs (Biozym). The PCR cycle started with an initial heating step of 98°C for 30sec, followed by 30 cycles at 98°C for 10sec, 62.6°C for 15sec, and 72°C for 30sec, and a final elongation step of 7min at 72°C. The correct length of the amplicon was assessed via gel electrophoresis and the band was excised from a 1%agarose gel and purified using the Wizard SV Gel and PCR Clean-Up System (Promega) according to manufacturer's instructions. The amplicon was eluted in 30µl ddH_2O and the DNA concentration was determined using a Nanodrop 2000 instrument (Thermo Scientific).

Preparation of the target vector for ligation. The pOPIN-M vector was obtained from Addgene (based on an MTA with the Chancellor, Masters and Scholars of the University of Oxford, Wellington Square, Oxford, OX1 2JD, UK, Ray Owens Lab). To prepare the vector for cloning, 1µg vector DNA was digested with 2U/µl of the restriction enzymes *KpnI*-HF (NEB) and *HindIII*-HF (NEB) according to manufacturer's instructions using an incubation time of 90min at 37°C. The desired band was excised from a 1% agarose gel and the linearized vector DNA was purified using the Wizard SV Gel and PCR Clean-Up System (Promega) according to manufacturer's instructions. The amplicon was eluted in 30µl ddH_2O and the DNA concentration was determined using a Nanodrop 2000 instrument (Thermo Scientific).

Ligation with the Gibson Assembly™ cloning kit (NEB). The reaction mix was prepared according to manufacturer's instructions. We used 50ng vector DNA and added the inserts at a 3x higher molarity in a final reaction volume of 20µl. The reaction was incubated for 1hr at

50°C, then 10µl of the cloning reaction were transformed into chemically competent *E.coli* XL1 Blue (Agilent) according to manufacturer's instructions and plated on LB-agar containing 100µg/mL ampicillin and 1mg/ml X-Gal (5-Brom-4-chlor-3-indoxyl-β-D-galactopyranosid). Screening for positive clones was performed by colony PCR and control restriction digests. The integrity of the final pOPIN-M construct containing the *YFP* gene, as well as the *Prdm9*^{Cst} gene downstream of the *MBP* gene was verified by sequencing. A single colony of the positive clone was inoculated in 3mL LB medium containing 100µg/mL ampicillin and an overnight culture was grown, shaking at 37°C. 2mL of the culture was harvested by centrifugation and a plasmid Miniprep was performed using the PureYield Plasmid Miniprep System (Promega) according to manufacturer's instructions.

Structure of the final construct. The primers used for amplification of *YFP* and *Prdm9*^{Cst} were designed such that several additional features were introduced to the final expression construct in the pOPIN-M vector, such as an N-terminal PreScission™ site that could be used to cleave off the His-MBP tag, as well as a TEV cleavage site that allows cleavage of the His-MBP-YFP tag, if necessary. Upstream of the *Prdm9*^{Cst}*Exon10*, a c-Myc-tag was introduced that allows for antibody-detection of the protein in a Western Blot. Furthermore, the *YFP* gene was flanked by two *XhoI* restriction sites, allowing fast and easy excision.

Below is a schematic representation of the modified pOPIN vector construct including restriction sites (*KpnI*, *XhoI*, *NotI*, *HindIII*), proteolytic cleavage sites (PreScission site, TEV site), and tags (the His-tag and MBP-tag are contained in the vector backbone, the eYFP-gene and the cMyc-tag were added in the cloning process of the *Prdm9*^{Cst}Exon10 coding sequence), TAA and TGA are stop-codons that were added after the open reading frame of the PRDM9 fusion protein.

pOPIN(His-MBP)vector backbone-*KpnI*-PreScission-*XhoI*-eYFP-*XhoI*-*NotI*-TEV-cMyc-*Prdm9*^{Cst}*Exon10*-TAA-TGA-*NotI*-*HindIII*-pOPINvector backbone

b.) Generation of a His-MBP-PRDM9^{Cst} *pOPIN-M construct-without YFP*

Excision of the *YFP* gene from the *YFP-Prdm9*^{Cst}_pOPIN-M construct. In order to produce a PRDM9 protein without the YFP-tag, we removed the *YFP* gene from the *YFP-Prdm9*^{Cst}_pOPIN-M construct by restriction-enzyme based cloning. 500ng vector DNA were subjected to a restriction enzyme digest using 1U/µl *XhoI* (NEB) in CutSmart buffer for 2hrs at 37°C, followed by a heat inactivation for 20min at 80°C. The digest was then separated on a 1% agarose gel and the desired band was excised. Then, the linearized vector DNA was purified using the Wizard SV Gel and PCR Clean-Up System (Promega) according to manufacturer's instructions using 30µl ddH_2O for the final elution and the DNA concentration was determined using a Nanodrop 2000 instrument (Thermo Scientific). 50ng of pure, linear vector DNA were re-ligated using 100U/µl T4 DNA ligase (NEB) in the supplied buffer by an overnight incubation at 16°C. Then, 5µl of the cloning reaction were transformed into chemically competent *E.coli* XL1 Blue (Agilent) according to manufacturer's instructions and plated on LB-agar containing 100µg/mL ampicillin. Screening for positive clones was performed by control restriction digests. The integrity of the final pOPIN-M construct containing the *Prdm9*^{Cst} gene downstream of the *MBP* gene was verified by sequencing. A single colony of the positive clone was inoculated in 3-mL LB medium containing 100µg/mL ampicillin and an overnight culture was grown, shaking at 37°C. 2mL of culture was

harvested by centrifugation and a plasmid Miniprep was performed using the PureYield Plasmid Miniprep System (Promega) according to manufacturer's instructions.

Below is a schematic representation of the modified pOPIN vector construct including restriction sites (*KpnI*, *XhoI*, *NotI*, *HindIII*), proteolytic cleavage sites (PreScission site, TEV site), and tags (the His-tag and MBP-tag are contained in the vector backbone, the cMyc-tag was added in the cloning process of the *Prdm9*^{Cst}Exon10 coding sequence), TAA and TGA are stop-codons that were added after the open reading frame of the PRDM9 fusion protein.

pOPIN(His-MBP)vector backbone-*KpnI*-PreScission-*XhoI*-*NotI*-TEV-cMyc-*Prdm9*^{Cst}*Exon10*-TAA-TGA-*NotI*-*HindIII*- pOPINvector backbone

c.) Generation of a PRDM9^{Cst} (full-length) and PRDM9^{Cst}-ZnF construct in the expression vector pT7-IRES-MycN

PCR amplification of the *Prdm9*^{Cst}insert and preparation of the insert for ligation. As first step, the *Prdm9*^{Cst} insert was amplified out of the pBAD construct (kindly provided by the Pektov Lab, Center for Genome Dynamics, The Jackson Laboratory, Bar Harbor, ME 04609, USA, see (Billings et al., 2013)) using the primer set mP9_fwd_XhoIAvi and mP9_rvs-BamHI for cloning the full-length *Prdm9* gene and mP9Ex10_F_XhoAv and mP9_rvs-BamHI for cloning only exon10 of *Prdm9* encoding the ZnF domains (primer sequences are shown in Supplementary_Table_S1). As polymerase 0.02 U/µl Phusion Hot Start II polymerase (Thermo Scientific) were used in a 100µl reaction in HF buffer supplemented with 0.2mM dNTPs (Biozym). 10^8 molecules vector DNA were used as template. The PCR cycle started with an initial heating step of 98°C for 30sec, followed by 5 cycles at 98°C for 10sec, 55°C for 15sec, and 72°C for 60sec, followed by 15 cycles at 98°C for 10sec, and 72°C for 75sec, concluded by a final elongation step of 7min at 72°C. The correct length of the amplicon was assessed via gel electrophoresis. The PCR reaction was purified using the Wizard SV Gel and PCR Clean-Up System (Promega) according to manufacturer's instructions. The amplicon was eluted in 30µl DNase/RNase-free, double destilled water (ddH_2O) and the DNA concentration was determined using a Nanodrop 2000 instrument (ThermoScientific). The pure PCR was then digested with 1.5U/µl of the restriction enzymes *XhoI* (NEB) and *BamHI*-HF (NEB) according to manufacturer's instructions using an incubation time of 90min at 37°C, followed by heat inactivation for 20min at 80°C.

Preparation of the target vector for ligation. The pT7-IRES-MycN vector (Takara) was prepared for ligation by performing a restriction enzyme digest. 1µg vector DNA was digested with 1U/µl of the restriction enzymes *XhoI* (NEB) and *BamHI*-HF (NEB) according to manufacturer's instructions using an incubation time of 90min at 37°C. Then, the vector was dephosphorylated using 0.2U/µl of Antarctic Phosphatase (NEB) according to manufacturer's instructions using an incubation time of 15min at 37°C, followed by a heat inactivation for 5min at 65°C. The desired band was excised from a 1% agarose gel using a clean scalpel and the linearized vector DNA was purified using the Wizard SV Gel and PCR Clean-Up System (Promega) according to manufacturer's instructions. The amplicon was eluted in 30µl ddH_2O and the DNA concentration was determined using a Nanodrop 2000 instrument (ThermoScientific).

Ligation of vector and insert. The ligation reaction was performed using the T4 ligase (NEB) in a 20µl reaction using 50ng vector DNA and a 3x higher molarity of the insert, incubating at 16°C overnight. Then 3µl of the ligation reaction were transformed into

chemically competent *E.coli* XL1 Blue (Agilent) according to manufacturer's instructions and plated on LB-agar containing 100µg/mL ampicillin. Screening for positive clones was performed by colony PCR and control restriction digests. The integrity of the final pT7-IRES-MycN construct, containing the full-length *Prdm9*^{Cst} gene or the ZnF-coding region, respectively, was verified by sequencing. A single colony of the positive clone was inoculated in 3mL LB medium containing 100µg/mL ampicillin and an overnight culture was grown, shaking at 37°C. 2mL of culture was harvested by centrifugation and a plasmid Miniprep was performed using the PureYield Plasmid Miniprep System (Promega) according to manufacturer's instructions.

Structure of the final constructs. The primers used for amplification of *Prdm9*^{Cst} were designed such that an additional Avi-tag was introduced in the construct, which was meant for downstream purification of the protein.

Full-length PRDM9^{Cst} (detailed sequence shown below)

pT7(IRES)cMycN vector backbone-cMyc-FactorXa-XhoI-AviTag-Prdm9^{Cst}**FL**-TAA-BamHI-pT7(IRES)cMycN vector backbone

ZnF domains of PRDM9^{Cst} (detailed sequence shown below)

pT7(IRES)cMycN vector backbone-cMyc-FactorXa-XhoI-AviTag-Prdm9^{Cst}**ZnF**-TAA-BamHI-pT7(IRES)cMycN vector backbone

d.) Introduction of His-YFP in the vector construct

PCR amplification of the *YFP* gene and preparation of the insert for ligation. The *YFP* insert was amplified using 10^8 molecules of the pEYFP-N1 vector (Clontech) and the primer set eGFP_fwd_XhoI and eGFP_rvs_XhoI (primer sequences are shown in Supplementary_Table_S1). As polymerase 0.02 U/µl Phusion Hot Start II polymerase (Thermo Scientific) were used in a 50µl reaction in HF buffer supplemented with 0.2mM dNTPs (Biozym). The PCR cycle started with an initial heating step of 98°C for 30sec, followed by 30 cycles at 98°C for 15sec, 60°C for 15sec, and 72°C for 15sec, and a final elongation step of 5min at 72°C. The correct length of the amplicon was assessed via gel electrophoresis and the band was excised from the agarose gel using a clean scalpel and purified using the Wizard SV Gel and PCR Clean-Up System (Promega) according to manufacturer's instructions. The amplicon was eluted in 30µl ddH_2O and the DNA concentration was determined using a Nanodrop 2000 instrument (ThermoScientific).

Preparation of the target vector for ligation. The pT7-IRES-MycN vector (containing the full-length *Prdm9*^{Cst} gene, or the ZnF-coding region, respectively) was prepared for ligation by performing a restriction enzyme digest. 1µg vector DNA was digested with 1U/µl of the restriction enzyme *XhoI* (NEB) according to manufacturer's instructions using an incubation time of 90min at 37°C. Then, the vector was dephosphorylated using 0.2U/µl of Antarctic Phosphatase (NEB) according to manufacturer's instructions using an incubation time of 15min at 37°C, followed by a heat inactivation for 20min at 65°C. The desired band was excised from a 1% agarose gel using a clean scalpel and the linearized vector DNA was

purified using the Wizard SV Gel and PCR Clean-Up System (Promega) according to manufacturer's instructions. The amplicon was eluted in 30µl ddH_2O and the DNA concentration was determined using a Nanodrop 2000 instrument (ThermoScientific).

Ligation of vector and insert. The ligation reaction was performed using the T4 ligase (NEB) in a 20µl reaction using 50ng vector DNA and a 3x higher molarity of the insert, incubating at 16°C overnight. Then 3µl of the ligation reaction were transformed into chemically competent *E.coli* XL1 Blue (Agilent) according to manufacturer's instructions and plated on LB-agar containing 100µg/mL ampicillin. Screening for positive clones (with the correct orientation of the *YFP* gene) was performed by colony PCR and control restriction digests. The integrity of the final pT7-IRES-MycN construct, containing the *YFP* gene as well as the *Prdm9*^{Cst} gene, was verified by Sanger sequencing (LGC genomics). A single colony of the positive clone was inoculated in 3mL LB medium containing 100µg/mL ampicillin and an overnight culture was grown, shaking at 37°C. 2mL of culture was harvested by centrifugation and a plasmid Miniprep was performed using the PureYield Plasmid Miniprep System (Promega) according to manufacturer's instructions.

Full-length PRDM9^{Cst}with N-terminal YFP (detailed sequence shown below)

pT7(IRES)cMycN vector backbone-cMyc-FactorXa-XhoI-His-YFP-XhoI-AviTag-Prdm9^{Cst}**FL**-TAA-BamHI-pT7(IRES)cMycN vector backbone

ZnF domains of PRDM9^{Cst}with N-terminal YFP (detailed sequence shown below)

pT7(IRES)cMycN vector backbone-cMyc-FactorXa-XhoI-His-YFP-XhoI-AviTag-Prdm9^{Cst}**ZnF**-TAA-BamHI-pT7(IRES)cMycN vector backbone

pOPIN-,pGEX-, and pT7-IRES-MycN- expression constructs

Sequence of pOPIN-M vector containing the coding region of murine *Prdm9*^{Cst}-Exon10, which encodes the single ZnF, as well as the ZnF domain at the C-terminal end of the protein.Capital letters indicate the open reading frame of the His-MBP-eYFP-PRDM9^{Cst}-ZnF-fusion construct.

```
ggcctctaaacgggtcttgaggggttttttgctgaaagcatgcggaggaaattctccttgaagt
ttccctggtgttcaaagtaaaggagtttgcaccagacgcacctctgttcactggtccggcgtat
taaaacacgatacattgttattagtacatttattaagcgctagattctgtgcgttgttgattta
cagacaattgttgtacgtattttaataattcattaaatttataatctttagggtggtatgttag
agcgaaaatcaaatgattttcagcgtctttatatctgaatttaaatattaaatcctcaatagat
ttgtaaaataggtttcgattagtttcaaacaagggttgtttttccgaaccgatggctggactat
ctaatggattttcgctcaacgccacaaaacttgccaaatcttgtagcagcaatctagctttgtc
gatattcgtttgtgttttgttttgtaataaaggttcgacgtcgttcaaaatattatgcgctttt
gtatttctttcatcactgtcgttagtgtacaattgactcgacgtaaacacgttaaatagagctt
ggacatatttaacatcgggcgtgttagctttattaggccgattatcgtcgtcgtcccaaccctc
gtcgttagaagttgcttccgaagacgattttgccatagccacacgacgcctattaattgtgtcg
gctaacacgtccgcgatcaaatttgtagttgagctttttggaattgcgatcgcataacttcgta
```

tagcatacattatacgaagttataagctcggaacgctgcgctcggtcgttcggctgcggcgagc
ggtatcagctcactcaaaggcggtaatacggttatccacagaatcaggggataacgcaggaaag
aacatgtgagcaaaaggccagcaaaaggccaggaaccgtaaaaaggccgcgttgctggcgtttt
tccataggctccgcccccctgacgagcatcacaaaaatcgacgctcaagtcagaggtggcgaaac
ccgacaggactataaagataccaggcgtttccccctggaagctccctcgtgcgctctcctgttc
cgaccctgccgcttaccggatacctgtccgcctttctcccttcgggaagcgtggcgctttctca
atgctcacgctgtaggtatctcagttcggtgtaggtcgttcgctccaagctgggctgtgtgcac
gaaccccccgttcagcccgaccgctgcgccttatccggtaactatcgtcttgagtccaacccgg
taagacacgacttatcgccactggcagcagccactggtaacaggattagcagagcgaggtatgt
aggcggtgctacagagttcttgaagtggtggcctaactacggctacactagaagaacagtattt
ggtatctgcgctctgctgaagccagttaccttcggaaaaagagttggtagctcttgatccggca
aacaaaccaccgctggtagcggtggtttttttgtttgcaagcagcagattacgcgcagaaaaaa
aggatctcaagaagatcctttgttaccaatgcttaatcagtgaggcacctatctcagcgatctg
tctatttcgttcatccatagttgcctgactccccgtcgtgtagataactacgatacgggagggc
ttaccatctggccccagtgctgcaatgataccgcgagacccacgctcaccggctccagatttat
cagcaataaaccagccagccggaagggccgagcgcagaagtggtcctgcaactttatccgcctc
catccagtctattaattgttgccgggaagctagagtaagtagttcgccagttaatagtttgcgc
aacgttgttgccattgctacaggcatcgtggtgtcacgctcgtcgtttggtatggcttcattca
gctccggttcccaacgatcaaggcgagttacatgatcccccatgttgtgcaaaaaagcggttag
ctccttcggtcctccgatcgttgtcagaagtaagttggccgcagtgttatcactcatggttatg
gcagcactgcataattctcttactgtcatgccatccgtaagatgcttttctgtgactggtgagt
actcaaccaagtcattctgagaatagtgtatgcggcgaccgagttgctcttgcccggcgtcaat
acgggataataccgcgccacatagcagaactttaaaagtgctcatcattggaaaacgttcttcg
gggcgaaaactctcaaggatcttaccgctgttgagatccagttcgatgtaacccactcgtgcac
ccaactgatcttcagcatcttttactttcaccagcgtttctgggtgagcaaaaacaggaaggca
aaatgccgcaaaaaagggaataagggcgacacggaaatgttgaatactcatactcttcctttt
caatattattgaagcatttatcagggttattgtctcatgtccgcgcgtttcctgcatcttttaa
tcaaatcccaagatgtgtataaacgcgccggtatgtacaggaagaggtttatactaaactgtta
cattgcaaacgtggtttcgtgtgccaagtgtgaaaaccgatgtttaatcaaggctctgacgcat
ttctacaaccacgactccaagtgtgtgggtgaagtcatgcatcttttaatcaaatcccaagatg
tgtataaaccaccaaactgccaaaaaatgaaaactgtcgacaagctctgtccgtttgctggcaa
ctgcaagggtctcaatcctatttgtaattattgaataataaaacaattataaatgtcaaatttg
tttttattaacgatacaaaccaaacgcaacaagaacatttgtagtattatctataattgaaaa
cgcgtagttataatcgctgaggtaatatttaaaatcattttcaaatgattcacagttaatttgc
gacaatataatttattttcacataaactagacgccttgtcgtcttcttcttcgtattccttct
ctttttcatttttctcttcataaaaattaacatagttattatcgtatccatatatgtatctatc
gtatagagtaaattttttgttgtcataaatatatatgtctttttttaatggggtgtatagtaccg
ctgcgcatagtttttctgtaatttacaacagtgctattttctggtagttcttcggagtgtgttg
ctttaattattaaatttatataatcaatgaatttgggatcgtcggttttgtacaatatgttgcc
ggcatagtacgcagcttcttctagttcaattacaccatttttttagcagcaccggattaacataa
ctttccaaaatgttgtacgaaccgttaaacaaaaacagttcacctccctttttctatactattgt
ctgcgagcagttgtttgttgttaaaaataacagccattgtaatgagacgcacaaactaatatca
caaactggaaatgtctatcaatatatagttgctgatggccggccgtaatgagacgcacaaacta
atatcacaaactggaaatgtctatcaatatatagttgctctagttattaatagtaatcaattac
ggggtcattagttcatagcccatatatggagttccgcgttacataacttacggtaaatggcccg
cctggctgaccgcccaacgacccccgcccattgacgtcaataatgacgtatgttcccatagtaa
cgccaatagggactttccattgacgtcaatgggtggagtatttacggtaaactgcccacttggc
agtacatcaagtgtatcatatgccaagtacgccccctattgacgtcaatgacggtaaatggccc
gcctggcattatgcccagtacatgaccttatgggactttcctacttggcagtacatctacgtat
tagtcatcgctattaccatgcatggtcgaggtgagccccacgttctgcttcactctccccatct
cccccccctccccacccccaattttgtatttatttattttttaattattttgtgcagcgatggg
ggcgggggggggggggggcgcgcgccaggcggggcggggcggggcgaggggcggggcggggcg
aggcggagaggtgcggcggcagccaatcagagcggcgcgctccgaaagtttccttttatggcga
ggcggcggcggcggcggccctataaaaagcgaagcgcgcggcgggcgggagtcgctgcgcgctg
ccttcgccccgtgccccgctccgccgccgcctcgcgccgcccgccccggctctgactgaccgcg
ttactcccacaggtgagcgggcgggacggcccttctccttcgggctgtaattagcgcttggttt
aatgacggcttgtttcttttctgtggctgcgtgaaagccttgaggggctccgggagggcccttt

gtgcgggggggagcggctcggggctgtccgcgggggggacggctgccttcgggggggacggggcag
ggcggggttcggcttctggcgtgtgaccggcggctctagagcctctgctaaccatgttcatgcc
ttcttctttttcctacagctcctgggcaacgtgctggttattgtgctgtctcatcattttggca
aagaattggatcggaccgaaattaatacgactcactataggggaattgtgagcggataacaatt
ccccggagttaatccgggacctttaattcaacccaacacaatatattatagttaaataagaatt
attatcaaatcatttgtatattaattaaaatactatactgtaaattacattttatttacaatca
aaggagatataccATGGCACACCATCACCACCATCACAGCAGCGGCATGAAAATCGAAGAAGGT
AAACTGGTAATCTGGATTAACGGCGATAAAGGCTATAACGGTCTCGCTGAAGTCGGTAAGAAAT
TCGAGAAAGATACCGGAATTAAAGTCACCGTTGAGCATCCGGATAAACTGGAAGAGAAATTCCC
ACAGGTTGCGGCAACTGGCGATGGCCCTGACATTATCTTCTGGGCACACGACCGCTTTGGTGGC
TACGCTCAATCTGGCCTGTTGGCTGAAATCACCCCGGACAAAGCGTTCCAGGACAAGCTGTATC
CGTTTACCTGGGATGCCGTACGTTACAACGGCAAGCTGATTGCTTACCCGATCGCTGTTGAAGC
GTTATCGCTGATTTATAACAAAGATCTGCTGCCGAACCCGCCAAAAACCTGGGAAGAGATCCCG
GCGCTGGATAAAGAACTGAAAGCGAAAGGTAAGAGCGCGCTGATGTTCAACCTGCAAGAACCGT
ACTTCACCTGGCCGCTGATTGCTGCTGACGGGGGTTATGCGTTCAAGTATGAAAACGGCAAGTA
CGACATTAAAGACGTGGGCGTGGATAACGCTGGCGCGAAAGCGGGTCTGACCTTCCTGGTTGAC
CTGATTAAAAACAAACACATGAATGCAGACACCGATTACTCCATCGCAGAAGCTGCCTTTAATA
AAGGCGAAACAGCGATGACCATCAACGGCCCGTGGGCATGGTCCAACATCGACACCAGCAAAGT
GAATTATGGTGTAACGGTACTGCCGACCTTCAAGGGTCAACCATCCAAACCGTTCGTTGGCGTG
CTGAGCGCAGGTATTAACGCCGCCAGTCCGAACAAAGAGCTGGCAAAAGAGTTCCTCGAAAACT
ATCTGCTGACTGATGAAGGTCTGGAAGCGGTTAATAAAGACAAACCGCTGGGTGCCGTAGCGCT
GAAGTCTTACGAGGAAGAGTTGGCGAAAGATCCACGTATTGCCGCCACTATGGAAAACGCCCAG
AAAGGTGAAATCATGCCGAACATCCCGCAGATGTCCGCTTTCTGGTATGCCGTGCGTACTGCGG
TGATCAACGCCGCCAGCGGTCGTCAGACTGTCGATGAAGCCCTGAAAGACGCGCAGACTAGCAG
CGGTCTGGAAGTTCTGTTTCAGGGTACCCTGGAAGTTCTGTTCCAGGGGCCCCTCGAGATGGTG
AGCAAGGGCGAGGAGCTGTTCACCGGGGTGGTGCCCATCCTGGTCGAGCTGGACGGCGACGTAA
ACGGCCACAAGTTCAGCGTGTCCGGCGAGGGCGAGGGCGATGCCACCTACGGCAAGCTGACCCT
GAAGTTCATCTGCACCACCGGCAAGCTGCCCGTGCCCTGGCCCACCCTCGTGACCACCTTCGGC
TACGGCCTGCAGTGCTTCGCCCGCTACCCCGACCACATGAAGCAGCACGACTTCTTCAAGTCCG
CCATGCCCGAAGGCTACGTCCAGGAGCGCACCATCTTCTTCAAGGACGACGGCAACTACAAGAC
CCGCGCCGAGGTGAAGTTCGAGGGCGACACCCTGGTGAACCGCATCGAGCTGAAGGGCATCGAC
TTCAAGGAGGACGGCAACATCCTGGGGCACAAGCTGGAGTACAACTACAACAGCCACAACGTCT
ATATCATGGCCGACAAGCAGAAGAACGGCATCAAGGTGAACTTCAAGATCCGCCACAACATCGA
GGACGGCAGCGTGCAGCTCGCCGACCACTACCAGCAGAACACCCCCATCGGCGACGGCCCCGTG
CTGCTGCCCGACAACCACTACCTGAGCTACCAGTCCGCCCTGAGCAAAGACCCCAACGAGAAGC
GCGATCACATGGTCCTGCTGGAGTTCGTGACCGCCGCCGGGATCACTCTCGGCATGGACGAGCT
GTACAAGCTCGAGGCGGCCGCAGAAAACTTGTATTTCCAGGGCGAGCAGAAGCTGATCTCAGAG
GAAGACCTGCTAAGGACAGAAATTCATCCTTGTCTTTTGTGCTCTTTGGCCTTCTCAAGTCAGA
AATTCCTCACTCAACATATGGAATGGAATCATCGCACTGAAATCTTCCCAGGAACATCTGCAAG
AATAAATCCTAAACCAGGAGATCCCTGTTCAGATCAGCTTCAGGAACAACATGTTGATTCACAG
AACAAAAATGACAAGGCCAGCAATGAAGTAAAAAGAAAATCCAAACCCAGGCAGAGGATTTCAA
CAACCTTTCCCAGCACACTCAAAGAACAAATGAGATCTGAGGAAAGTAAGAGAACTGTGGAAGA
GCTCAGAACAGGCCAGACAACAAATACAGAGGACACAGTCAAATCATTTATTGCATCAGAAATC
TCAAGTATTGAAAGACAATGTGGGCAATATTTCAGTGATAAGTCAAATGTCAATGAGCACCAGA
AGACACACACAGGGGAGAAGCCCTATGTTTGCAGGGAGTGTGGGCGGGGCTTTACAGCGAAGTC
AAACCTCATCCAGCACCAGAGGACACACACAGGGGAGAAGCCCTATGTTTGCAGGGAGTGTGGG
CGGGGCTTTACACAGAAGTCAGTCCTCATCCAGCACCAGAGGACACACACAGGGGAGAAGCCCT
ATGTTTGCAGGGAGTGTGGGCGGGGCTTTACACAGAAGTCAGACCTCATCAAGCACCAGAGGAC
ACACACAGGGGAGAAGCCCTATGTTTGCAGGGAGTGTGGGCGGGGCTTTACAGCGAAGTCAAAC
CTCATCCAGCACCAGAGGACACACACAGGGGAGAAGCCCTATGTTTGCAGGGAGTGTGGGCGGG
GCTTTACAGAGAAGTCAAGCCTCATCAAGCACCAGAGGACACACACAGGGGAGAAGCCCTATGT
TTGCAGGGAGTGTGGGTGGGGCTTTACAGCGAAGTCAAACCTCATCCAGCACCAGAGGACACAC
ACAGGGGAGAAGCCCTATGTTTGCAGGGAGTGTGGGCGGGGCTTTACACAGAAGTCAAGCCTCA
TCAAGCACCAGAGGACACACACAGGGGAGAAGCCCTATGTTTGCAGGGAGTGTGGGCGGGGCTT
TACAGCGAAGTCAAACCTCATCCAGCACCAGAGGACACACACAGGGGAGAAGCCCTATGTTTGC
AGGGAGTGTGGGTGGGGCTTTACACAGAAGTCAAACCTCATCAAGCACCAGAGGACACACACGG
GGGAGAAGCCCTATGTTTGCAGGGAGTGTGGGTGGGGCTTTACACAGAAGTCAGACCTCATCCA

```
GCACCAGAGGCCACATACAAGAGAGAAGTAAtgagcggccgcaagctttctagaccagtttaaa
catcaccatcaccatcactaagtgattaacctcaggtgcaggctgcctatcagaaggtggtggc
tggtgtggccaatgccctggctcacaaataccactgagatcgatctttttccctctgccaaaaa
ttatggggacatcatgaagccccttgagcatctgacttctggctaataaaggaaatttattttc
attgcaatagtgtgttggaattttttgtgtctctcactcggaaggacatatgggagggcaaatc
atttaaaacatcagaatgagtatttggtttagagtttggcaacatatgcccatatgtaactagc
ataaccccttgg
```

Sequence of pGEX-6P2 vector containing the coding region of murine *Prdm9*^{Cst}-Exon10, which encodes the single ZnF, as well as the ZnF domain at the C-terminal end of the protein.Capital letters indicate the open reading frame of the GST-eGFP-PRDM9^{Cst}-ZnF-fusion construct.Letters in bold denote the annealing sites of the IF_gfp-mPRD9_2_F primer *and of the IF_mPR9-opin_2_R primer*

acgttatcgactgcacggtgcaccaatgcttctggcgtcaggcagccatcggaagctgtggtat
ggctgtgcaggtcgtaaatcactgcataattcgtgtcgctcaaggcgcactcccgttctggata
atgttttttgcgccgacatcataacggttctggcaaatattctgaaatgagctgttgacaatta
atcatcggctcgtataatgtgtggaattgtgagcggataacaatttcacacaggaaacagtatt
cATGTCCCCTATACTAGGTTATTGGAAAATTAAGGGCCTTGTGCAACCCACTCGACTTCTTTTG
GAATATCTTGAAGAAAAATATGAAGAGCATTTGTATGAGCGCGATGAAGGTGATAAATGGCGAA
ACAAAAAGTTTGAATTGGGTTTGGAGTTTCCCAATCTTCCTTATTATATTGATGGTGATGTTAA
ATTAACACAGTCTATGGCCATCATACGTTATATAGCTGACAAGCACAACATGTTGGGTGGTTGT
CCAAAAGAGCGTGCAGAGATTTCAATGCTTGAAGGAGCGGTTTTGGATATTAGATACGGTGTTT
CGAGAATTGCATATAGTAAAGACTTTGAAACTCTCAAAGTTGATTTTCTTAGCAAGCTACCTGA
AATGCTGAAAATGTTCGAAGATCGTTTATGTCATAAAACATATTTAAATGGTGATCATGTAACC
CATCCTGACTTCATGTTGTATGACGCTCTTGATGTTGTTTTATACATGGACCCAATGTGCCTGG
ATGCGTTCCCAAAATTAGTTTGTTTTAAAAAACGTATTGAAGCTATCCCACAAATTGATAAGTA
CTTGAAATCCAGCAAGTATATAGCATGGCCTTTGCAGGGCTGGCAAGCCACGTTTGGTGGTGGC
GACCATCCTCCAAAATCGGATCTGGAAGTTCTGTTCCAGGGGCCCCTGGGATCCCCAGGAATTC
CCGGGTCGACTCGAGCG**GAAAACTTGTATTTCCAGGGCGAG**CAGAAGCTGATCTCAGAGGAAGA
CCTGCTAAGGACAGAAATTCATCCTTGTCTTTTGTGCTCTTTGGCCTTCTCAAGTCAGAAATTC
CTCACTCAACATATGGAATGGAATCATCGCACTGAAATCTTCCCAGGAACATCTGCAAGAATAA
ATCCTAAACCAGGAGATCCCTGTTCAGATCAGCTTCAGGAACAACATGTTGATTCACAGAACAA
AAATGACAAGGCCAGCAATGAAGTAAAAAGAAAATCCAAACCCAGGCAGAGGATTTCAACAACC
TTTCCCAGCACACTCAAAGAACAAATGAGATCTGAGGAAAGTAAGAGAACTGTGGAAGAGCTCA
GAACAGGCCAGACAACAAATACAGAGGACACAGTCAAATCATTTATTGCATCAGAAATCTCAAG
TATTGAAAGACAATGTGGGCAATATTTCAGTGATAAGTCAAATGTCAATGAGCACCAGAAGACA
CACACAGGGGAGAAGCCCTATGTTTGCAGGGAGTGTGGGCGGGGCTTTACAGCGAAGTCAAACC
TCATCCAGCACCAGAGGACACACACAGGGGAGAAGCCCTATGTTTGCAGGGAGTGTGGGCGGGG
CTTTACACAGAAGTCAGTCCTCATCCAGCACCAGAGGACACACACAGGGGAGAAGCCCTATGTT
TGCAGGGAGTGTGGGCGGGGCTTTACACAGAAGTCAGACCTCATCAAGCACCAGAGGACACACA
CAGGGGAGAAGCCCTATGTTTGCAGGGAGTGTGGGCGGGGCTTTACAGCGAAGTCAAACCTCAT
CCAGCACCAGAGGACACACACAGGGGAGAAGCCCTATGTTTGCAGGGAGTGTGGGCGGGGCTTT
ACAGAGAAGTCAAGCCTCATCAAGCACCAGAGGACACACACAGGGGAGAAGCCCTATGTTTGCA
GGGAGTGTGGGTGGGGCTTTACAGCGAAGTCAAACCTCATCCAGCACCAGAGGACACACACAGG
GGAGAAGCCCTATGTTTGCAGGGAGTGTGGGCGGGGCTTTACACAGAAGTCAAGCCTCATCAAG
CACCAGAGGACACACACAGGGGAGAAGCCCTATGTTTGCAGGGAGTGTGGGCGGGGCTTTACAG
CGAAGTCAAACCTCATCCAGCACCAGAGGACACACACAGGGGAGAAGCCCTATGTTTGCAGGGA
GTGTGGGTGGGGCTTTACACAGAAGTCAAACCTCATCAAGCACCAGAGGACACACACGGGGGAG
AAGCCCTATGTTTGCAGGGAGTGTGGGTGGGGCTTTACACAGAAGTCAGACCTCATCCAGCACC
AGAGGCCACATACAAGA***GAGAAGTAAtgagcggccgca***tcgtgactgactgacgatctgcctcg
cgcgtttcggtgatgacggtgaaaacctctgacacatgcagctcccggagacggtcacagcttg
tctgtaagcggatgccgggagcagacaagcccgtcagggcgcgtcagcgggtgttggcgggtgt
cggggcgcagccatgacccagtcacgtagcgatagcggagtgtataattcttgaagacgaaagg
gcctcgtgatacgcctatttttataggttaatgtcatgataataatggtttcttagacgtcagg

tggcacttttcggggaaatgtgcgcggaacccctatttgtttatttttctaaatacattcaaat
atgtatccgctcatgagacaataaccctgataaatgcttcaataatattgaaaaaggaagagta
tgagtattcaacatttccgtgtcgcccttattcccttttttgcggcattttgccttcctgtttt
tgctcacccagaaacgctggtgaaagtaaaagatgctgaagatcagttgggtgcacgagtgggt
tacatcgaactggatctcaacagcggtaagatccttgagagttttcgccccgaagaacgttttc
caatgatgagcacttttaaagttctgctatgtggcgcggtattatcccgtgttgacgccgggca
agagcaactcggtcgccgcatacactattctcagaatgacttggttgagtactcaccagtcaca
gaaaagcatcttacggatggcatgacagtaagagaattatgcagtgctgccataaccatgagtg
ataacactgcggccaacttacttctgacaacgatcggaggaccgaaggagctaaccgcttttt
gcacaacatgggggatcatgtaactcgccttgatcgttgggaaccggagctgaatgaagccata
ccaaacgacgagcgtgacaccacgatgcctgcagcaatggcaacaacgttgcgcaaactattaa
ctggcgaactacttactctagcttcccggcaacaattaatagactggatggaggcggataaagt
tgcaggaccacttctgcgctcggcccttccggctggctggtttattgctgataaatctggagcc
ggtgagcgtgggtctcgcggtatcattgcagcactggggccagatggtaagccctcccgtatcg
tagttatctacacgacggggagtcaggcaactatggatgaacgaaatagacagatcgctgagat
aggtgcctcactgattaagcattggtaactgtcagaccaagtttactcatatatactttagatt
gatttaaaacttcatttttaatttaaaaggatctaggtgaagatcctttttgataatctcatga
ccaaaatcccttaacgtgagttttcgttccactgagcgtcagaccccgtagaaaagatcaaagg
atcttcttgagatcctttttttctgcgcgtaatctgctgcttgcaaacaaaaaaaccaccgcta
ccagcggtggtttgtttgccggatcaagagctaccaactctttttccgaaggtaactggcttca
gcagagcgcagataccaaatactgtccttctagtgtagccgtagttaggccaccacttcaagaa
ctctgtagcaccgcctacatacctcgctctgctaatcctgttaccagtggctgctgccagtggc
gataagtcgtgtcttaccgggttggactcaagacgatagttaccggataaggcgcagcggtcgg
gctgaacggggggttcgtgcacacagcccagcttggagcgaacgacctacaccgaactgagata
cctacagcgtgagctatgagaaagcgccacgcttcccgaagggagaaaggcggacaggtatccg
gtaagcggcagggtcggaacaggagagcgcacgagggagcttccagggggaaacgcctggtatc
tttatagtcctgtcgggtttcgccacctctgacttgagcgtcgatttttgtgatgctcgtcagg
ggggcggagcctatggaaaaacgccagcaacgcggcctttttacggttcctggccttttgctgg
ccttttgctcacatgttctttcctgcgttatcccctgattctgtggataaccgtattaccgcct
ttgagtgagctgataccgctcgccgcagccgaacgaccgagcgcagcgagtcagtgagcgagga
agcggaagagcgcctgatgcggtattttctccttacgcatctgtgcggtatttcacaccgcata
aattccgacaccatcgaatggtgcaaaacctttcgcggtatggcatgatagcgcccggaagaga
gtcaattcagggtggtgaatgtgaaaccagtaacgttatacgatgtcgcagagtatgccggtgt
ctcttatcagaccgtttcccgcgtggtgaaccaggccagccacgtttctgcgaaaacgcgggaa
aaagtggaagcggcgatggcggagctgaattacattcccaaccgcgtggcacaacaactggcgg
gcaaacagtcgttgctgattggcgttgccacctccagtctggccctgcacgcgccgtcgcaaat
tgtcgcggcgattaaatctcgcgccgatcaactgggtgccagcgtggtggtgtcgatggtagaa
cgaagcggcgtcgaagcctgtaaagcggcggtgcacaatcttctcgcgcaacgcgtcagtgggc
tgatcattaactatccgctggatgaccaggatgccattgctgtggaagctgcctgcactaatgt
tccggcgttatttcttgatgtctctgaccagacacccatcaacagtattattttctcccatgaa
gacggtacgcgactgggcgtggagcatctggtcgcattgggtcaccagcaaatcgcgctgttag
cgggcccattaagttctgtctcggcgcgtctgcgtctggctggctggcataaatatctcactcg
caatcaaattcagccgatagcggaacgggaaggcgactggagtgccatgtccggttttcaacaa
accatgcaaatgctgaatgagggcatcgttcccactgcgatgctggttgccaacgatcagatgg
cgctgggcgcaatgcgcgccattaccgagtccgggctgcgcgttggtgcggatatctcggtagt
gggatacgacgataccgaagacagctcatgttatatcccgccgtcaaccaccatcaaacaggat
tttcgcctgctggggcaaaccagcgtggaccgcttgctgcaactctctcagggccaggcggtga
agggcaatcagctgttgcccgtctcactggtgaaaagaaaaaccaccctggcgcccaatacgca
aaccgcctctccccgcgcgttggccgattcattaatgcagctggcacgacaggtttcccgactg
gaaagcgggcagtgagcgcaacgcaattaatgtgagttagctcactcattaggcaccccaggct
ttacactttatgcttccggctcgtatgttgtgtggaattgtgagcggataacaatttcacacag
gaaacagctatgaccatgattacggattcactggccgtcgttttacaacgtcgtgactgggaaa
accctggcgttacccaacttaatcgccttgcagcacatccccctttcgccagctggcgtaatag
cgaagaggcccgcaccgatcgcccttcccaacagttgcgcagcctgaatggcgaatggcgcttt
gcctggtttccggcaccagaagcggtgccggaaagctggctggagtgcgatcttcctgaggccg
atactgtcgtcgtcccctcaaactggcagatgcacggttacgatgcgcccatctacaccaacgt
aacctatcccattacggtcaatccgccgtttgttcccacggagaatccgacgggttgttactcg

ctcacatttaatgttgatgaaagctggctacaggaaggccagacgcgaattatttttgatggcg
ttggaatt

Sequence of pT7-IRES-MycN vector containing the coding region of YFP and the murine *Prdm9*^Cst^ (full-length). Capital letters indicate the open reading frame of the Myc-Avi-His-YFP-PRDM9^Cst^-fusion construct.Letters in italicdenote the YFP coding region.

gacgaaagggcctcgtgatacgcctatttttataggttaatgtcatgataataatggtttctta
gacgtcaggtggcacttttcggggaaatgtgcgcggaacccctatttgtttatttttctaaata
cattcaaatatgtatccgctcatgagacaataaccctgataaatgcttcaataatattgaaaaa
ggaagagtatgagtattcaacatttccgtgtcgcccttattcccttttttgcggcattttgcct
tcctgtttttgctcacccagaaacgctggtgaaagtaaaagatgctgaagatcagttgggtgca
cgagtgggttacatcgaactggatctcaacagcggtaagatccttgagagttttcgccccgaag
aacgttttccaatgatgagcacttttaaagttctgctatgtggcgcggtattatcccgtattga
cgccgggcaagagcaactcggtcgccgcatacactattctcagaatgacttggttgagtactca
ccagtcacagaaaagcatcttacggatggcatgacagtaagagaattatgcagtgctgccataa
ccatgagtgataacactgcggccaacttacttctgacaacgatcggaggaccgaaggagctaac
cgcttttttgcacaacatgggggatcatgtaactcgccttgatcgttgggaaccggagctgaat
gaagccataccaaacgacgagcgtgacaccacgatgcctgtagcaatggcaacaacgttgcgca
aactattaactggcgaactacttactctagcttcccggcaacaattaatagactggatggaggc
ggataaagttgcaggaccacttctgcgctcggcccttccggctggctggtttattgctgataaa
tctggagccggtgagcgtgggtctcgcggtatcattgcagcactggggccagatggtaagccct
cccgtatcgtagttatctacacgacggggagtcaggcaactatggatgaacgaaatagacagat
cgctgagataggtgcctcactgattaagcattggtaactgtcagaccaagtttactcatatata
ctttagattgatttaaaacttcatttttaatttaaaaggatctaggtgaagatcctttttgata
atctcatgaccaaaatcccttaacgtgagttttcgttccactgagcgtcagaccccgtagaaaa
gatcaaaggatcttcttgagatcctttttttctgcgcgtaatctgctgcttgcaaacaaaaaaa
ccaccgctaccagcggtggtttgtttgccggatcaagagctaccaactctttttccgaaggtaa
ctggcttcagcagagcgcagataccaaatactgttcttctagtgtagccgtagttaggccacca
cttcaagaactctgtagcaccgcctacatacctcgctctgctaatcctgttaccagtggctgct
gccagtggcgataagtcgtgtcttaccgggttggactcaagacgatagttaccggataaggcgc
agcggtcgggctgaacggggggttcgtgcacacagcccagcttggagcgaacgacctacaccga
actgagatacctacagcgtgagctatgagaaagcgccacgcttcccgaagggagaaaggcggac
aggtatccggtaagcggcagggtcggaacaggagagcgcacgagggagcttccagggggaaacg
cctggtatctttatagtcctgtcgggtttcgccacctctgacttgagcgtcgatttttgtgatg
ctcgtcaggggggcggagcctatggaaaaacgccagcaacgcggcctttttacggttcctggcc
ttttgctggccttttgctcacatgttctttcctgcgttatcccctgattctgtggataaccgta
ttaccgcctttgagtgagctgataccgctcgccgcagccgaacgaccgagcgcagcgagtcagt
gagcgaggaagcggaagagcgcccaatacgcaaaccgcctctccccgcgcgttggccgattcat
taatgcagctggcacgacaggtttcccgactggaaagcgggcagtgagcgcaacgcaattaatg
tgagttagctcactcattaggcaccccaggctttacactttatgcttccggctcgtatgttgtg
tggaattgtgagcggataacaatttcacacaggaaacagctatgaccatgattacgccaagctt
taatacgactcactataggcaaccatgataacgttactggccgaagccgcttggaataaggccg
gtgtgcgtttgtctatatgttattttccaccatattgccgtcttttggcaatgtgagggcccgg
aaacctggccctgtcttcttgacgagcattcctaggggtctttcccctctcgccaaaggaatgc
aaggtctgttgaatgtcgtgaaggaagcagttcctctggaagcttcttgaagacaaacaacgtc
tgtagcgaccctttgcaggcagcggaaccccccacctggcgacaggtgcctctgcggccaaaag
ccacgtgtataagatacacctgcaaaggcggcacaaccccagtgccacgttgtgagttggatag
ttgtggaaagagtcaaatggctctcctcaagcgtattcaacaaggggctgaaggatgcccagaa
ggtaccccattgtatgggatctgatctggggcctcggtgcacatgctttacatgtgtttagtcg
aggttaaaaaacgtctaggccccccgaaccacggggacgtggttttcctttgaaaaacacgatg
ataatatggccacaaccATGGCTAGCGAGCAGAAGCTGATCTCAGAGGAGGACCTGATCGAAGG
GCGCCATATGGAGCTCCTCGAG*CATCATCATCATCATCATATGGTGAGCAAGGGCGAGGAGCTG*
TTCACCGGGGTGGTGCCCATCCTGGTCGAGCTGGACGGCGACGTAAACGGCCACAAGTTCAGCG
TGTCCGGCGAGGGCGAGGGCGATGCCACCTACGGCAAGCTGACCCTGAAGTTCATCTGCACCAC

CGGCAAGCTGCCCGTGCCCTGGCCCACCCTCGTGACCACCTTCGGCTACGGCCTGCAGTGCTTC
GCCCGCTACCCCGACCACATGAAGCAGCACGACTTCTTCAAGTCCGCCATGCCCGAAGGCTACG
TCCAGGAGCGCACCATCTTCTTCAAGGACGACGGCAACTACAAGACCCGCGCCGAGGTGAAGTT
CGAGGGCGACACCCTGGTGAACCGCATCGAGCTGAAGGGCATCGACTTCAAGGAGGACGGCAAC
ATCCTGGGGCACAAGCTGGAGTACAACTACAACAGCCACAACGTCTATATCATGGCCGACAAGC
AGAAGAACGGCATCAAGGTGAACTTCAAGATCCGCCACAACATCGAGGACGGCAGCGTGCAGCT
CGCCGACCACTACCAGCAGAACACCCCCATCGGCGACGGCCCCGTGCTGCTGCCCGACAACCAC
TACCTGAGCTACCAGTCCGCCCTGAGCAAAGACCCCAACGAGAAGCGCGATCACATGGTCCTGC
*TGGAGTTCGTGACCGCCGCCGGGATCACTCTCGGCATGGACGAGCTGTACAAG*CTCGAGATGTC
CGGCCTGAACGACATCTTCGAGGCTCAGAAAATCGAATGGCACGAAATGAACACCAACAAGCTG
GAAGAAAATAGTCCTGAAGAAGATACAGGGAAATTCGAGTGGAAACCCAAGGTCAAAGATGAAT
TCAAAGACATTTCCATATACTTCTCCAAAGAAGAATGGGCAGAGATGGGAGAGTGGGAGAAAAT
TCGCTATAGAAATGTGAAAAGGAACTATAAAATGCTGATTTCAATAGGTCTCAGAGCCCCTAGA
CCAGCTTTCATGTGTTACCAAAGGCAAGCAATGAAACCCCAAATAAATGACAGTGAGGATTCTG
ATGAAGAGTGGACACCTAAGCAACAAGTCAGTCCTCCTTGGGTGCCTTTCCGGGTGAAGCACAG
TAAGCAGCAGAAGGAATCATCTAGAATGCCATTTAGTGGTGAATCTAATGTGAAGGAAGGGTCT
GGAATAGAAAATTTGCTGAATACAAGTGGCTCAGAACACGTCCAGAAACCAGTGTCCTCCCTTG
AAGAAGGAAATACCTCTGGACAGCACTCTGGGAAAAAACTGAAACTTAGGAAAAAGAACGTTGA
AGTGAAAATGTACAGGCTGCGAGAGAGAAAGGGCCTTGCCTATAAGGAGGTCAGCGAGCCTCAG
GATGATGACTATCTCTATTGTGAGAAGTGCCAGAATTTCTTCATCGATAGTTGTCCCAACCATG
GGCCTCCTTTATTTGTAAAAGACAGTATGGTGGACAGGGGGCATCCCAACCACTCAGTCCTCAG
TCTGCCCCCTGGGCTAAGAATTAGTCCATCGGGCATCCCTGAAGCTGGACTTGGAGTATGGAAT
GAAGCATCTGATCTACCAGTCGGTCTGCACTTTGGCCCCTATGAGGGTCAGATCACAGAGGATG
AAGAGGCAGCCAACAGTGGTTACTCCTGGCTGATTACCAAGGGAAGAAACTGCTATGAGTATGT
GGATGGACAGGACGAGTCCCAAGCCAACTGGATGAGGTATGTGAACTGTGCCCGGGATGATGAA
GAGCAGAACCTGGTAGCCTTTCAATATCACAGGAAAATCTTCTATCGAACCTGCCGGGTTATCA
GACCAGGTTGTGAGCTTCTGGTCTGGTATGGGGATGAGTACGGCCAGGAACTGGGCATTAAGTG
GGGAAGCAAGATGAAGAAAGGATTCACAGCAGGAAGAGAACTAAGGACAGAAATTCATCCTTGT
CTTTTGTGCTCTTTGGCCTTCTCAAGTCAGAAATTCCTCACTCAACATATGGAATGGAATCATC
GCACTGAAATCTTCCCAGGAACATCTGCAAGAATAAATCCTAAACCAGGAGATCCCTGTTCAGA
TCAGCTTCAGGAACAACATGTTGATTCACAGAACAAAAATGACAAGGCCAGCAATGAAGTAAAA
AGAAAATCCAAACCCAGGCAGAGGATTTCAACAACCTTTCCCAGCACACTCAAAGAACAAATGA
GATCTGAGGAAAGTAAGAGAACTGTGGAAGAGCTCAGAACAGGCCAGACAACAAATACAGAGGA
CACAGTCAAATCATTTATTGCATCAGAAATCTCAAGTATTGAAAGACAATGTGGGCAATATTTC
AGTGATAAGTCAAATGTCAATGAGCACCAGAAGACACACACAGGGGAGAAGCCCTATGTTTGCA
GGGAGTGTGGGCGGGGCTTTACAGCGAAGTCAAACCTCATCCAGCACCAGAGGACACACACAGG
GGAGAAGCCCTATGTTTGCAGGGAGTGTGGGCGGGGCTTTACACAGAAGTCAGTCCTCATCCAG
CACCAGAGGACACACACAGGGGAGAAGCCCTATGTTTGCAGGGAGTGTGGGCGGGGCTTTACAC
AGAAGTCAGACCTCATCAAGCACCAGAGGACACACACAGGGGAGAAGCCCTATGTTTGCAGGGA
GTGTGGGCGGGGCTTTACAGCGAAGTCAAACCTCATCCAGCACCAGAGGACACACACAGGGGAG
AAGCCCTATGTTTGCAGGGAGTGTGGGCGGGGCTTTACAGAGAAGTCAAGCCTCATCAAGCACC
AGAGGACACACACAGGGGAGAAGCCCTATGTTTGCAGGGAGTGTGGGTGGGGCTTTACAGCGAA
GTCAAACCTCATCCAGCACCAGAGGACACACACAGGGGAGAAGCCCTATGTTTGCAGGGAGTGT
GGGCGGGGCTTTACACAGAAGTCAAGCCTCATCAAGCACCAGAGGACACACACAGGGGAGAAGC
CCTATGTTTGCAGGGAGTGTGGGCGGGGCTTTACAGCGAAGTCAAACCTCATCCAGCACCAGAG
GACACACACAGGGGAGAAGCCCTATGTTTGCAGGGAGTGTGGGTGGGGCTTTACACAGAAGTCA
AACCTCATCAAGCACCAGAGGACACACACGGGGGAGAAGCCCTATGTTTGCAGGGAGTGTGGGT
GGGGCTTTACACAGAAGTCAGACCTCATCCAGCACCAGAGGCCACATACAAGAGAGAAGTAAgg
atccgaattcactagtgtcgacctgcagtctagataggtaatctctgaaaaaaaaaaaaaaaaa
aaaaaaaaaaaaaaactagcataaccccttggggcctctaaacgggtcttgaggggttttttgga
tcatccgcggccgctaattcactggccgtcgttttacaacgtcgtgactgggaaaaccctggcg
ttacccaacttaatcgccttgcagcacatccccctttcgccagctggcgtaatagcgaagaggc
ccgcaccgatcgcccttcccaacagttgcgcagcctgaatggcgaatggcgcctgatgcggtat
tttctccttacgcatctgtgcggtatttcacaccggtgcactctcagtacaatctgctctgatg
ccgcatagttaagccagccccgacacccgccaacacccgctgacgcgccctgacgggcttgtct
gctcccggcatccgcttacagacaagctgtgaccgtctccgggagctgcatgtgtcagaggttt
tcaccgtcatcaccgaaacgcgcga

Sequence of pT7-IRES-MycN vector containing the coding region of YFP and the murine *Prdm9*^{Cst}-ZnF. Capital letters indicate the open reading frame of the Myc-Avi-His-YFP-PRDM9^{Cst}-ZnF-fusion construct.Letters in italicdenote the YFP coding region.

gacgaaagggcctcgtgatacgcctatttttataggttaatgtcatgataataatggtttctta
gacgtcaggtggcacttttcggggaaatgtgcgcggaacccctatttgtttatttttctaaata
cattcaaatatgtatccgctcatgagacaataaccctgataaatgcttcaataatattgaaaaa
ggaagagtatgagtattcaacatttccgtgtcgcccttattcccttttttgcggcattttgcct
tcctgtttttgctcacccagaaacgctggtgaaagtaaaagatgctgaagatcagttgggtgca
cgagtgggttacatcgaactggatctcaacagcggtaagatccttgagagttttcgccccgaag
aacgttttccaatgatgagcacttttaaagttctgctatgtggcgcggtattatcccgtattga
cgccgggcaagagcaactcggtcgccgcatacactattctcagaatgacttggttgagtactca
ccagtcacagaaaagcatcttacggatggcatgacagtaagagaattatgcagtgctgccataa
ccatgagtgataacactgcggccaacttacttctgacaacgatcggaggaccgaaggagctaac
cgcttttttgcacaacatgggggatcatgtaactcgccttgatcgttgggaaccggagctgaat
gaagccataccaaacgacgagcgtgacaccacgatgcctgtagcaatggcaacaacgttgcgca
aactattaactggcgaactacttactctagcttcccggcaacaattaatagactggatggaggc
ggataaagttgcaggaccacttctgcgctcggcccttccggctggctggtttattgctgataaa
tctggagccggtgagcgtgggtctcgcggtatcattgcagcactggggccagatggtaagccct
cccgtatcgtagttatctacacgacggggagtcaggcaactatggatgaacgaaatagacagat
cgctgagataggtgcctcactgattaagcattggtaactgtcagaccaagtttactcatatata
ctttagattgatttaaaacttcatttttaatttaaaaggatctaggtgaagatcctttttgata
atctcatgaccaaaatcccttaacgtgagttttcgttccactgagcgtcagaccccgtagaaaa
gatcaaaggatcttcttgagatcctttttttctgcgcgtaatctgctgcttgcaaacaaaaaaa
ccaccgctaccagcggtggtttgtttgccggatcaagagctaccaactctttttccgaaggtaa
ctggcttcagcagagcgcagataccaaatactgttcttctagtgtagccgtagttaggccacca
cttcaagaactctgtagcaccgcctacatacctcgctctgctaatcctgttaccagtggctgct
gccagtggcgataagtcgtgtcttaccgggttggactcaagacgatagttaccggataaggcgc
agcggtcgggctgaacggggggttcgtgcacacagcccagcttggagcgaacgacctacaccga
actgagatacctacagcgtgagctatgagaaagcgccacgcttcccgaagggagaaaggcggac
aggtatccggtaagcggcagggtcggaacaggagagcgcacgagggagcttccagggggaaacg
cctggtatctttatagtcctgtcgggtttcgccacctctgacttgagcgtcgatttttgtgatg
ctcgtcaggggggcggagcctatggaaaaacgccagcaacgcggcctttttacggttcctggcc
ttttgctggccttttgctcacatgttctttcctgcgttatcccctgattctgtggataaccgta
ttaccgcctttgagtgagctgataccgctcgccgcagccgaacgaccgagcgcagcgagtcagt
gagcgaggaagcggaagagcgcccaatacgcaaaccgcctctccccgcgcgttggccgattcat
taatgcagctggcacgacaggtttcccgactggaaagcgggcagtgagcgcaacgcaattaatg
tgagttagctcactcattaggcaccccaggctttacactttatgcttccggctcgtatgttgtg
tggaattgtgagcggataacaatttcacacaggaaacagctatgaccatgattacgccaagctt
taatacgactcactataggcaaccatgataacgttactggccgaagccgcttggaataaggccg
gtgtgcgtttgtctatatgttattttccaccatattgccgtcttttggcaatgtgagggcccgg
aaacctggccctgtcttcttgacgagcattcctaggggtctttcccctctcgccaaaggaatgc
aaggtctgttgaatgtcgtgaaggaagcagttcctctggaagcttcttgaagacaaacaacgtc
tgtagcgaccctttgcaggcagcggaaccccccacctggcgacaggtgcctctgcggccaaaag
ccacgtgtataagatacacctgcaaaggcggcacaaccccagtgccacgttgtgagttggatag
ttgtggaaagagtcaaatggctctcctcaagcgtattcaacaaggggctgaaggatgcccagaa
ggtaccccattgtatgggatctgatctggggcctcggtgcacatgctttacatgtgtttagtcg
aggttaaaaaacgtctaggccccccgaaccacggggacgtggttttcctttgaaaaacacgatg
ataatATGGCCACAACCATGGCTAGCGAGCAGAAGCTGATCTCAGAGGAGGACCTGATCGAAGG
GCGCCATATGGAGCTCCTCGAG*CATCATCATCATCATCATATGGTGAGCAAGGGCGAGGAGCTG*
TTCACCGGGGTGGTGCCCATCCTGGTCGAGCTGGACGGCGACGTAAACGGCCACAAGTTCAGCG
TGTCCGGCGAGGGCGAGGGCGATGCCACCTACGGCAAGCTGACCCTGAAGTTCATCTGCACCAC
CGGCAAGCTGCCCGTGCCCTGGCCCACCCTCGTGACCACCTTCGGCTACGGCCTGCAGTGCTTC
GCCCGCTACCCCGACCACATGAAGCAGCACGACTTCTTCAAGTCCGCCATGCCCGAAGGCTACG
TCCAGGAGCGCACCATCTTCTTCAAGGACGACGGCAACTACAAGACCCGCGCCGAGGTGAAGTT
CGAGGGCGACACCCTGGTGAACCGCATCGAGCTGAAGGGCATCGACTTCAAGGAGGACGGCAAC

ATCCTGGGGCACAAGCTGGAGTACAACTACAACAGCCACAACGTCTATATCATGGCCGACAAGC
AGAAGAACGGCATCAAGGTGAACTTCAAGATCCGCCACAACATCGAGGACGGCAGCGTGCAGCT
CGCCGACCACTACCAGCAGAACACCCCCATCGGCGACGGCCCCGTGCTGCTGCCCGACAACCAC
TACCTGAGCTACCAGTCCGCCCTGAGCAAAGACCCCAACGAGAAGCGCGATCACATGGTCCTGC
*TGGAGTTCGTGACCGCCGCCGGGATCACTCTCGGCATGGACGAGCTGTACAAG*CTCGAGATGTC
CGGCCTGAACGACATCTTCGAGGCTCAGAAAATCGAATGGCACGAACTAAGGACAGAAATTCAT
CCTTGTCTTTTGTGCTCTTTGGCCTTCTCAAGTCAGAAATTCCTCACTCAACATATGGAATGGA
ATCATCGCACTGAAATCTTCCCAGGAACATCTGCAAGAATAAATCCTAAACCAGGAGATCCCTG
TTCAGATCAGCTTCAGGAACAACATGTTGATTCACAGAACAAAAATGACAAGGCCAGCAATGAA
GTAAAAAGAAAATCCAAACCCAGGCAGAGGATTTCAACAACCTTTCCCAGCACACTCAAAGAAC
AAATGAGATCTGAGGAAAGTAAGAGAACTGTGGAAGAGCTCAGAACAGGCCAGACAACAAATAC
AGAGGACACAGTCAAATCATTTATTGCATCAGAAATCTCAAGTATTGAAAGACAATGTGGGCAA
TATTTCAGTGATAAGTCAAATGTCAATGAGCACCAGAAGACACACACAGGGGAGAAGCCCTATG
TTTGCAGGGAGTGTGGGCGGGGCTTTACAGCGAAGTCAAACCTCATCCAGCACCAGAGGACACA
CACAGGGGAGAAGCCCTATGTTTGCAGGGAGTGTGGGCGGGGCTTTACACAGAAGTCAGTCCTC
ATCCAGCACCAGAGGACACACACAGGGGAGAAGCCCTATGTTTGCAGGGAGTGTGGGCGGGGCT
TTACACAGAAGTCAGACCTCATCAAGCACCAGAGGACACACACAGGGGAGAAGCCCTATGTTTG
CAGGGAGTGTGGGCGGGGCTTTACAGCGAAGTCAAACCTCATCCAGCACCAGAGGACACACACA
GGGGAGAAGCCCTATGTTTGCAGGGAGTGTGGGCGGGGCTTTACAGAGAAGTCAAGCCTCATCA
AGCACCAGAGGACACACACAGGGGAGAAGCCCTATGTTTGCAGGGAGTGTGGGTGGGGCTTTAC
AGCGAAGTCAAACCTCATCCAGCACCAGAGGACACACACAGGGGAGAAGCCCTATGTTTGCAGG
GAGTGTGGGCGGGGCTTTACACAGAAGTCAAGCCTCATCAAGCACCAGAGGACACACACAGGGG
AGAAGCCCTATGTTTGCAGGGAGTGTGGGCGGGGCTTTACAGCGAAGTCAAACCTCATCCAGCA
CCAGAGGACACACACAGGGGAGAAGCCCTATGTTTGCAGGGAGTGTGGGTGGGGCTTTACACAG
AAGTCAAACCTCATCAAGCACCAGAGGACACACACGGGGGAGAAGCCCTATGTTTGCAGGGAGT
GTGGGTGGGGCTTTACACAGAAGTCAGACCTCATCCAGCACCAGAGGCCACATACAAGAGAGAA
GTAAggatccgaattcactagtgtcgacctgcagtctagataggtaatctctgaaaaaaaaaaa
aaaaaaaaaaaaaaaaaaactagcataaccccttggggcctctaaacgggtcttgaggggttt
tttggatcatccgcggccgctaattcactggccgtcgttttacaacgtcgtgactgggaaaacc
ctggcgttacccaacttaatcgccttgcagcacatccccctttcgccagctggcgtaatagcga
agaggcccgcaccgatcgcccttcccaacagttgcgcagcctgaatggcgaatggcgcctgatg
cggtattttctccttacgcatctgtgcggtatttcacaccggtgcactctcagtacaatctgct
ctgatgccgcatagttaagccagccccgacacccgccaacacccgctgacgcgccctgacgggc
ttgtctgctcccggcatccgcttacagacaagctgtgaccgtctccgggagctgcatgtgtcag
aggttttcaccgtcatcaccgaaacgcgcga

Recombinant expression of PRDM9^{Cst} in bacterial cells and lysate preparation

In order to find the most suitable expression system to produce a functional PRDM9 protein, we screened several different systems (such as cell-free *in vitro* expression, bacterial expression, mammalian expression, as well as insect cell expression). Finally, we decided on recombinant bacterial expression, which was the best system in terms of protein yield, as well as cost efficiency. We used the pOPIN-M vector containing *Prdm9*^{Cst} gene (both with and without the YFP-tag) in combination with the *E.coli* strain Rosetta^{TM}2(DE3)pLacI (Novagen, Merck). This vector is especially suited for the expression of difficult genes that have a toxic effect during bacterial growth. The cells contain an additional plasmid pLacI that encodes the lac-repressor that specifically binds to the lac-operator to suppress transcription. In combination with the pOPIN-M vector, which contains the lac-operator next to the T7-promoter (that drives the bacterial expression of our PRDM9 insert), the lac-repressor reduces basal expression of PRDM9, thereby enhancing and facilitating bacterial growth.

The *YFP-Prdm9*^{Cst}_pOPIN-M and the *Prdm9*^{Cst}_pOPIN-M vector constructs were each transformed into chemically competent *E.coli* Rosetta^{TM}2(DE3)pLacI (Novagen, Merck) according to manufacturer's instructions and plated on LB-agar containing 100µg/mL ampicillin and 34µg/mL chloramphenicol in order to select for both plasmids (pOPIN and pLacI). A single colony was then inoculated in 500ml LB (Lysogeny broth after Lennox: 5g/L yeast extract, 5g/L NaCl, 10g/L tryptone) containing 100µg/mL ampicillin and 34µg/mL chloramphenicol overnight, shaking with 165rpm at 37°C. On the following day, the growth curve was monitored until OD600 = 1. This usually took up to 20hrs (despite the lac-repressor the cells grew very slowly). Then, the cells were harvested by centrifugation at 5000rpm for 5min and a medium change was performed. The growth (selection) medium was decanted and replaced by fresh LB-medium equilibrated to room temperature (RT) and supplemented with 1mM IPTG (Isopropyl-beta-D-thiogalactopyran) and 50µM $ZnCl_2$ and protein expression was performed for 7hrs at RT, shaking at 165rpm. Then, the cells were harvested by centrifugation at 5000rpm for 10min at 4°C and the pellets were frozen at -80°C at least overnight in order to have one freeze-thaw cycle to enhance cell lysis.

Lysate preparation for His-MBP-YFP-PRDM9^{Cst}-ZnF. The pellets were thawed on ice and the wet weight was determined. 10ml wash buffer (1xTBS: 25mM Tris base, 137mM NaCl, 2.7mM KCl, pH 7.4) were used to resuspend 0.5g pellet. Since PRDM9 turned out to be completely insoluble after bacterial expression, this step was used to wash out all soluble proteins. Then, the cellular components were harvested again by centrifugation at 5000rpm for 10min at 4°C. The supernatant was analyzed by SDS-PAGE and contained only little amounts of PRDM9. The pellet was resuspended in 10ml 1xTBS+0.3%Sarcosyl(N-Lauroylsarcosine) supplemented with 1x Protease inhibitor cocktail (Promega) and the soluble fraction, containing PRDM9, (also referred to as SN* for supernatant) was obtained by centrifugation at 5000rpm for 10min at 4°C. Then, the pellet was resuspended once more in 10ml 1xTBS+0.3%Sarcosyl resulting in the whole-cell lysate (also referred to as WC*). The protein lysates were aliquoted and stored at -80°C. The proper molecular weight of the protein was assessed by SDS-PAGE and Capillary Western and the approximate PRDM9 concentration was determined by Capillary Western using a His-tagged standard protein for calibration (see below and Supplementary_Fig_S1).Note: Several attempts to purify PRDM9^{Cst} via affinity purification failed or resulted in loss of protein function. Therefore we designed the experiments such that they are compatible with working in cell lysates.

Lysate preparation for His-MBP-PRDM9^{Cst}-ZnF-without YFP. The pellets were thawed on ice and the wet weight was determined. 10ml wash buffer (1xTKZN buffer: 10mM Tris, 50mM KCl, 50µM $ZnCl_2$, 0.05% NP40, pH 7.5) were used to resuspend 0.5g pellet. Since PRDM9 turned out to be completely insoluble after bacterial expression, this step was used to wash out all soluble proteins. Then, the cellular components were harvested again by centrifugation at 5000rpm for 10min at 4°C. The supernatant was analyzed by SDS-PAGE and contained only little amounts of PRDM9. The pellet was resuspended in 750µl 1xTKZN+0.3%Sarcosyl and the soluble fraction, containing PRDM9, (also referred to as SN* for supernatant) was obtained by centrifugation at 5000rpm for 10min at 4°C. This procedure was repeated to obtain multiple supernatant fractions (SN*1-3) that contain high amounts of soluble PRDM9^{Cst}-ZnF. Then, the pellet was resuspended once more in 10ml 1xTKZN+0.3%Sarcosyl resulting in the whole-cell lysate (also referred to as WC*). The protein lysates were aliquoted and stored at -80°C. The proper molecular weight of the protein was assessed by SDS-PAGE and Capillary Western and the approximate PRDM9 concentration was determined by Capillary Western using a His-tagged standard protein for calibration (see below and Supplementary_Fig_S1).

Cell-free *in vitro* expression of His-YFP-PRDM9^{Cst} and (His-YFP)-PRDM9^{Cst}-ZnF

The Human Cell-Free Protein Expression System (Takara) was used in order to express the complete (full-length) PRDM9 or only the ZnF domain (His-YFP-PRDM9^{Cst} and His-YFP-PRDM9^{Cst}-ZnF, respectively). For this purpose, 300ng plasmid DNA of the respective pT7-IRES-MycN construct was used and the protein was expressed following the manufacturer's instructions. In short, 9µl Cell Lysate, 6µl Mixture-1 and 1µl Mixture-2 were mixed and incubated for 10 minutes at room temperature. Then, 2µl Mixture-3, 300ng DNA, 1µl T7 RNA Polymerase and 100ng $ZnCl_2$ were added. The reaction was incubated at 32°C for 6hrs. PRDM9 concentration was determined by Capillary Western or conventional Western Blot. The concentration of His-YFP-PRDM9^{Cst} was estimated to be 7.28µM, the concentration of His-YFP-PRDM9^{Cst}-ZnF was estimated to be 29.2µM, and the concentration of PRDM9^{Cst}-ZnF was estimated to be 36.6µM.

PRDM9 quantification (Capillary Western) and verification of protein integrity (SDS-PAGE)

In order to verify the molecular weight of PRDM9 after recombinant expression, the lysates were analyzed via SDS-polyacrylamide gel electrophoresis. Therefore the samples were mixed with Laemmli buffer (10% glycerol, 2% SDS, 80mM Tris-HCl pH 6.8, 5.3% beta-mercaptoethanol, 0.06% Bromophenol blue), incubated at RT for 5min (in order not to destroy the eYFP chromophore we did NOT incubate at 95°C) and loaded onto 8% SDS-polyacrylamide gels which were run at 180V until the Bromophenol blue front has run out completely. In case of eYPF-fusion proteins, the fluorescence at 510nm was detected directly in the gel using the ChemiDoc™ MP imager (Bio-Rad) with the blot settings "Cy2" (see Supplementary_Fig_S1).

In order to estimate the PRDM9 concentration in our crude cell lysates, a quantitative Western was performed at the ProTech division of VBCF (Vienna Biocenter Core Facilities GmbH), Vienna. Therefore 7.5µl of appropriately diluted sample were mixed with 2.5µl loading buffer (20mM Bicine pH 7.6/0.6% CHAPS/1%SDS/40mM DTT + fluorescent standard proteins) and the samples were heated to 95°C for 5min and spun down for 1min at 5000rpm. Then, the samples, as well as a biotinylated protein marker, were transferred to a 384-well plate and the Capillary Western was run according to the manufacturer's instructions (Device: Peggy, Company: Protein Simple). The samples were separated for 40min at 250V. Subsequently, the samples were immobilized to the capillary wall for 200sec, then the matrix was removed and washed 3x for 150sec. Blocking was performed for 23min, followed by 2 wash steps for 150sec. Then, the capillary was incubated with primary antibody (Penta-His antibody, Qiagen in a 1:100 dilution) for 120min and washed twice for 150sec, followed by an incubation with secondary antibody (anti-mouse-HRP; horse raddish peroxidase) for 60min and 2 washes for 150sec. In case of the biotinylated marker, a Streptavidin-HRP antibody was used. Finally, the proteins were detected by a chemiluminescent reaction (luminol/peroxide) at 6 different exposure times.

We analyzed several different PRDM9 lysates, which all contained His-tagged PRDM9 proteins, in parallel with His-GFP standards of known concentration (125µg/ml – 25µg/ml). The approximate PRDM9 concentration was estimated by Coomassie staining and eYFP fluorescence prior to the Capillary Western and the lysates were diluted accordingly. The chemiluminescence, as a quantitative measure of His-tagged molecules present in the samples, was analyzed using the Compass software from Protein Simple and the PRDM9 concentration was inferred from the standard curve. The measurement was performed in duplicates.

The concentration of the His-MBP-PRDM9^{Cst}-ZnF lysate was estimated to be 22.84µM (lane 2) and the concentration of His-MBP-eYFP-PRDM9^{Cst}-ZnF was estimated to be 49.31µM.

EMSA

a.) EMSA – protein titrations (K_D determination)

Amplification of labeled target DNA. In these assays the complex formation of PRDM9^{Cst}-ZnF to the mouse hotspot *Hlx1*^{B6}, *Hlx1*^{Cst} and usDNA was assessed. The target DNAs were produced via 2 rounds of PCR using the biotinylated primers Bio-Hlx1-75bp_F and Bio-Hlx1-75bp_R for *Hlx1*^{B6}and *Hlx1*^{Cst} and the primers Bio-negctrl_F and Bio-negctrl_R (primer sequences are shown in Supplementary_Table_S1) In the first PCR round 1ng/µl genomic DNA of the mouse strain C57BL/6J (B6) or CAST/EiJ was used as template (kindly provided by the Pektov Lab, Center for Genome Dynamics, The Jackson Laboratory, Bar Harbor, ME 04609, USA) and 1ng/µl human genomic DNA was used to produce the negative control DNA (usDNA). As polymerase 0.004 units of the OneTaq Hot Start DNA polymerase (NEB) were used in a 50µl reaction in 1x OneTaq Standard Reaction Buffer supplemented with 0.2mM dNTPs (Biozym). The PCR cycle started with an initial heating step of 94°C for 30sec, followed by 30 cycles at 94°C for 15sec, 60°C for 15sec, and 68°C for 10sec, and a final elongation step of 5min at 68°C. The correct length of the amplicon was assessed via gel electrophoresis.

Exonuclease I digest and purification of the PCR products. In order to get rid of single stranded DNA molecules and primers, an Exonuclease I digest was performed. 8 units Exonuclease I (NEB) were used to digest 40µl of PCR product supplemented with an appropriate buffer and incubated at 37°C for 30min, followed by a heat inactivation of the enzyme at 80°C for 20min. Subsequently, the PCR product was purified using the Wizard SV Gel and PCR Clean-Up System (Promega) according to manufacturer's instructions. The amplicon was eluted in 40µl ddH_2O and the DNA concentration was determined using a Nanodrop 2000 instrument (Thermo Scientific).

Second round of PCR. The second round of PCR was performed to increase reproducibility between PCR reactions, as the genomic template can differ in quality between experiments. The reagents and conditions of the second PCR were equal to the first PCR, except that 10^{10} molecules of first-round-PCR amplicon were used as template. The PCR cycle started with an initial heating step of 94°C for 30sec, followed by 25 cycles at 94°C for 15sec, 60°C for 15sec, and 68°C for 10sec, and a final elongation step of 5min at 68°C. The correct length of the amplicon was assessed via gel electrophoresis. The Exonuclease I digest and the column purification were performed as described above.

EMSA reactions. The EMSA reactions for the protein titrations were performed in 1xTKZN binding buffer (10mM Tris, 50mM KCl, 50µM $ZnCl_2$, 0.05% NP40, pH 7.5). First of all, a master mix containing 3nM of labeled DNA was prepared and 18µl were distributed equally to the reaction tubes. Secondly, the PRDM9^{Cst}-ZnF lysate SN*2 (SN=supernatant) (see section "Recombinant expression of PRDM9" for details) was diluted to 2.15µM and supplemented with 46,96ng/µl of the non-specific competitor PolydIdC (Sigma-Aldrich) containing a final concentration of 0.3% Sarcosyl. Next, a dilution series with a dilution factor 1:1.5 was prepared using the protein buffer 1xTKZN+Sarcosyl (10mM Tris, 50mM KCl, 50µM $ZnCl_2$, 0.05% NP40, 0.3%Sarcosyl, pH 7.5). Finally, 2µl of the protein dilutions were added to the master mix and mixed well by pipetting up-and-down. The reactions were incubated for ~90hrs at 4°C.

General EMSA protocol: as described in the main paper.

Image analysis. Images with exposure times of 5sec and gamma values of 0.5 were used for analysis with the Image Lab software (Bio-Rad). At first, the lanes and bands were defined manually then the pixel intensities and values for fraction bound (%) were quantified and analyzed further using OriginPro8.5 (Origin Lab). The equation to calculate the fraction bound (%) = shift/(shift+unbound)*100. This average fraction bound was determined using the data of two-three replicate measurements and was plotted against the PRDM9 concentration at a semi-logarithmic scale. The sigmoidal curve was fitted using the following equation that describes receptor-ligand binding in solution in consideration of the total (fixed) receptor concentration added to the assay (reviewed in (Hulme and Trevethick, 2010)): y=A/2*(R+L+Kd-((R+L+K_D)^2-4*R*L)^(0.5))+B, where A is an amplitude fit parameter, R is the total DNA concentration, L is the PRDM9 concentration, K_D is the dissociation constant and B is an additive constant.

b.) EMSA – competition assay

Experimental setup of EMSA competition assay. To assess the PRDM9-DNA complex stability of the murine PRDM9^{Cst} zinc finger domain to the *Hlx1*^{B6} hotspot, a competition

assay was performed using Electrophoretic Mobility Shift Assays. Therefore, different concentrations (2284 or 150nM) of the PRDM9^{Cst}-ZnF domain were incubated with 10nM of a biotinylated 75bp DNA fragment of the *Hlx1^{B6}* hotspot (hot). The binding was competed by adding 100 fold excess of an unlabeled 39bp DNA fragment of the *Hlx1^{B6}* hotspot (cold) at different time points followed by distinct incubation times. Each experiment was performed in at least two replicates.

Production of the DNA fragments. The hot 75bp *Hlx1^{B6}* DNA fragment was produced in 2 successive PCR reactions, to get a pure product, using the biotin-labeled primers Bio-Hlx1-75bp_F and Bio-Hlx1-75bp_R (see Supplementary_Table_S1), as described in the section above.

The cold or unlabeled 39bp *Hlx1^{B6}* DNA fragment was ordered as lyophilized, HPSF purified single-stranded synthetic complementary oligonucleotides at the company Eurofins (see Supplementary_Table_S1). The oligos were resuspended in hybridization buffer (10mM Tris, 50mM KCl, 1mM DTT, pH 7.5) and equal amounts of forward and reverse strands were mixed and hybridized, starting with 3min at 98°C following with a temperature decrease of 1°C/minute to form double-stranded DNA fragments.

Exonuclease I digest and purification of the PCR and hybridized products. In order to get rid of single stranded DNA molecules and primers, 40µl PCR reaction and 100µl hybridized DNA reaction were digested using the Exonuclease I as it was previously described. Wizard SV Gel and PCR Clean-Up System (Promega) was used to purify the digested products according to manufacturer's instructions. The DNA fragments were eluted in 30-50µl ddH_2O and the DNA concentration was determined using a Nanodrop 2000 instrument (Thermo Scientific).

EMSA reaction. The EMSA binding reaction was performed using the following buffer conditions: 10mM Tris-HCl (pH 7.5), 50mM KCl, 50ng/µl polydIdC, 0.05% NP-40, 50µM $ZnCl_2$. The binding components of 10nM hot DNA, 1µM cold DNA and 2284nM or 150nM of PRDM9^{Cst}-ZnF lysate SN*2 (SN=supernatant; see section "Recombinant expression of PRDM9" for details) were added either simultaneously to the binding reaction and incubated 1hr at RT, or hot DNA and protein were incubated for 1hr, whereas cold DNA was added afterwards for an additional incubation time of 1 or 14hrs. In each experiment one reaction with only the biotin-labeled DNA (lane 1) was performed and incubated for 1h at RT. The EMSA protocol was continued as described previously.

Image analysis. Images with exposure times of 1sec and gamma values of 0.5 were used for analysis with the Image Lab software (Bio-Rad). The intensities of the shifted and unbound bands of each reaction were measured and the fraction bound [%] was calculated by [shift]/[shift+unbound]*100.

c.) EMSA – Experiments with Chimera fragments (Figure 4)

Generation of chimeric DNA-target sequences (*Hlx1^{B6}* truncations) by PCR. To assess which nucleotide-ZnF contacts confer binding specificity in the PRDM9^{Cst}-*Hlx1*^{B6} complex, we shortened the 31bp target binding site of *Hlx1*^{B6} in 5-nucleotide steps based on a 75bp fragment consisting of the *Hlx1*^{B6} target binding site and unspecific flanking sites (referred to as *Chimera nt 1-31*) (see Figure 4). The fragments are named after the nucleotide range of the 31bp binding site that is present in the chimeric fragment (e.g. *Chim. nt 1-16* contains nucleotides 1-16 of the 31bp binding site, thus lacks nucleotides 17-31). When shortening the target binding site of *Chim. nt 1-31* in 5nt steps, the specific bases were exchanged by a negative control DNA (referred to as usDNA), that does not interact with PRDM9^{Cst}, to maintain the total length of 75bp for all chimeric fragments. At least 2 successive PCR reactions were performed to produce the chimeric DNA fragments. To ensure purity of the products, 3-4 consecutive reactions were necessary. PCR conditions are shown in Supplementary_Table_S2. For most of the Chimera fragments a unique set of overlapping primers was used for the first PCR round, resulting in an extended fragment. For the rest of the fragments genomic DNA was used as template. To extend the flanking sites, biotinylated primers were used which also partly overlapped with the previously produced short fragments. For all reactions 0.75units/50µl *OneTaq* Hot Start DNA Polymerase, 1x *OneTaq* Standard Reaction Buffer (20mM Tris-HCl, 22mM NH_4Cl, 22mM KCl, 1.8mM $MgCl_2$, 0.06% IGEPAL CA-630, 0.05% Tween 20, pH 8.9 at 25°C), 200µM dNTPs were used in a 50µl reaction volume. The **Exonuclease I digest and purification of the PCR products** was performed as described above.

EMSA reaction. The EMSA binding reaction was performed using the following buffer conditions: 10mM Tris-HCl pH 7.5, 50mM KCl, 1mM DTT, 50ng/µl polydIdC, 0.05% NP-40, 50µM $ZnCl_2$. The binding components of 15nM hot DNA and 2.5µM of His-MBP-eYFP-PRDM9^{Cst}-ZnF protein whole-cell lysate (referred to as WC*; see section "Recombinant expression of PRDM9" for details) in 1xTBS+0.3% Sarcosyl were added to the binding reaction and incubated for 20min at RT. In each experiment one reaction with only the biotin-labeled DNA was performed. The EMSA protocol was continued as previously described. Each binding reaction was at least performed in triplicates.

Image analysis. Images with exposure times of 1sec and gamma values of 0.5 were used for analysis with the Image Lab software (Bio-Rad). The intensities of the unbound and shifted bands were measured in each lane and the fraction bound [%] was calculated by [shift]/[shift+unbound]*100.

d.) EMSA – Simultaneous hot and cold DNA competition assays (Figure 5)

Experimental setup of EMSA competition assays. In order to determine the binding specificity of different fragments at the murine *Hlx1*^{B6} hotspot to the murine PRDM9^{Cst} zinc finger domain, competition assays were performed using EMSA (Electrophoretic Mobility Shift Assay). Therefore the binding of the PRDM9^{Cst} zinc finger domain to a biotin-labeled 75bp DNA fragment (referred to as hot DNA) of the *Hlx1*^{B6} hotspot was recorded in a series of 9 binding reactions by additionally adding an increasing amount of a certain unlabeled

DNA fragment (referred to as cold DNA). A band shifted to the DNA band without protein indicates PRDM9 binding. The cold fragment differs in each experiment and was titrated from 0 to 100-fold excess according to the concentration of the hot fragment and therefore competes for PRDM9 ZnF binding. With increasing amount of the cold DNA, the shifted band decreases at different rates depending on the sequence specificity.

Production of the DNA fragments. The hot DNA fragment was produced in 2 successive PCR reactions, to get a pure product, using the biotin-labeled primers Bio-Hlx1-75bp_F and Bio-Hlx1-75bp_R (see Supplementary_Table_S1) as it was described in a section above. For all reactions 0.75units/50µl *OneTaq* Hot Start DNA Polymerase, 1X *OneTaq* Standard Reaction Buffer (20mM Tris-HCl, 22mM NH_4Cl, 22mM KCl, 1.8mM $MgCl_2$, 0.06% IGEPAL CA-630, 0.05% Tween 20, pH 8.9 at 25°C), 200µM dNTPs were used in a 50µl reaction volume.

The cold or unlabeled DNA fragments were ordered as lyophilized, HPSF purified single-stranded synthetic complementary oligonucleotides at the company Eurofins which were then hybridized, as described above.

Exonuclease I digest and purification of the PCR and hybridized products was performed as described above.

EMSA reaction. The EMSA binding reaction was performed using the following buffer conditions: 10mM Tris-HCl pH 7.5, 50mM KCl, 1mM DTT, 50ng/µl polydIdC, 0.05% NP-40, 50µM $ZnCl_2$. The binding components of 15nM hot DNA, 0-1500nM cold DNA and 250nM of His-MBP-eYFP-PRDM9^{Cst}-ZnF protein whole-cell lysate (referred to as WC*; see section “Recombinant expression of PRDM9” for details) in 1xTBS+0.3% Sarcosyl were added simultaneously to the binding reaction and incubated for 1hr at RT. In each experiment one reaction with only the biotin-labeled DNA (lane 1) and one reaction without the cold DNA (lane 2 - referred to as reference band) was performed. The EMSA protocol was continued as previously described. All experiments using different cold DNA fragments were performed at least in triplicates.

Image analysis. Images with exposure times of 1sec and gamma values of 0.5 were used for analysis with the Image Lab software (Bio-Rad). The intensities of the shifted bands were measured and the relation of each band to the reference band without the addition of cold DNA (see lane 2 of Figure 5) was calculated (referred to as relative intensity). Using OriginPro8.5, the relative intensities were plotted against the increasing concentration of the cold competitor in a semi-logarithmic graph and fitted with an exponential function (ExpDec1).

e.) EMSA – Time course (Supplementary_Fig_S4)

Experimental setup of EMSA time course. In order to test at which time point the binding of the murine PRDM9^{Cst} zinc finger domain to a 75bp DNA fragment of the *Hlx1*^{B6} hotspot

reaches equilibrium, a series of binding reactions with increasing incubation times have been performed using Electrophoretic Mobility Shift Assays. The fraction bound was calculated and the equilibrium was determined. To additionally assess the dependence of complex formation on protein concentration, two different experiments have been performed using 2500nM and 250nM PRDM9 (Supplementary_Fig_S4, panel A and B, respectively).

Production of the DNA fragments. The 75bp *Hlx1*^{B6} DNA fragment was produced in a PCR reaction, using biotin-labeled primers Bio-Hlx1-75bp_F and Bio-Hlx1-75bp_R (see Supplementary_Table_S1). For all reactions 0.75units/50µl *OneTaq* Hot Start DNA Polymerase, 1X *OneTaq* Standard Reaction Buffer (20mM Tris-HCl, 22mM NH_4Cl, 22mM KCl, 1.8mM $MgCl_2$, 0.06% IGEPAL CA-630, 0.05% Tween 20, pH 8.9 at 25°C), 200µM dNTPs were used in a 50µl reaction volume. As template (A) 10ng/µl genomic DNA and 1µM forward and reverse primer (B) or 10^{10} copies of a synthetic 75bp *Hlx1*^{B6} fragment and 0.05µM forward and reverse primer were used for the amplification.

Exonuclease I digest and purification of the PCR product. In order to get rid of single stranded DNA molecules and primers, an Exonuclease I digest was performed followed by column purification as described previously.

EMSA reaction. The EMSA binding reaction was performed using the following buffer conditions: 10mM Tris-HCl pH 7.5, 50mM KCl, 1mM DTT, 50ng/µl polydIdC, 0.05% NP-40, 50µM $ZnCl_2$. The binding components of (A) 5nM biotinylated DNA and 2500nM of His-MBP-eYFP-PRDM9^{Cst}-ZnF protein whole-cell lysate in 1xTBS + 0.3% Sarcosyl or (B) 15nM biotinylated DNA and 250nM of His-MBP-eYFP-PRDM9^{Cst}-ZnF protein whole-cell lysate in 1xTBS + 0.3% Sarcosyl were added simultaneously to the binding reaction and incubated for 5min, 10min, 20min, 30min, 45min, 1hr, 2hrs, and overnight at RT, as well as overnight at 4°C. In each experiment one reaction with only the biotin-labeled DNA (lane 1), incubated overnight at 4°C, was performed. The EMSA protocol was continued as described previously.

Image analysis. Images with exposure times of (A) 2sec or (B) 0.5sec and gamma values of 0.5 were used for analysis with the (A) MetaMorph software (Molecular Devices) or (B) Image Lab software (Bio-Rad). The intensities of the shifted and unbound bands of each reaction were measured and the fraction bound [%] was calculated by [shift]/[shift+unbound]*100.

Supplementary_Statistical_Analysis

Statistical Modeling of Binding Footprint Data

Here we present the results of our statistical analysis concerning the binding trends of competitors of different lengths. Our data consist of relative binding intensities in dependence on the concentration of the respective cold competitor, and are shown as six curves in Figure 5B.

We will consider in particular the following hypotheses:

1. Competitors "28bp-d" and "28bp-u" behave differently from the other curves, since the footprint is smaller than predicted from the number of ZnF in the array.

2. Competitor "34bp" behaves differently from "75bp"

3. "31bp" behaves differently to "75bp".

As these hypotheses have been proposed after looking at the data, we need to correct for multiple testing. We do this by noting that there are 25 – 1 = 31 possible partitions of the considered set of 6 curves into two nonempty subsets that could be compared. Furthermore there are $\binom{6}{2}$ = 15 choices for comparing pairs of curves. Although probably not all these comparisons make sense, considering all sets of potential candidate models is on the conservative side with respect to controlling for type I errors. A simple multiple test procedure that takes care of the potential tests enumerated above is the Bonferroni correction that controls for the familywise error, i.e. the probability of one or more false rejections. We thus applied a Bonferroni correction to the p-values obtained for our chosen hypotheses, and multiplied the subsequently computed p-values by the respective correction factor.

Our statistical tests also required an appropriate statistical model for the data. Our initial modeling attempts revealed that the measurements showed non-homogeneous variances with smaller variances for higher cold competitor concentrations. There were also signs of auto-correlation between subsequent concentration levels. We therefore used generalized least squares models. The null model only had the cold competitor concentration (as factor variable) as a predictor; whereas, the alternative model also included an indicator coding the two compared curves (or groups of curves). Both null and alternative model included an AR1 auto-correlation structure, and variances that were fitted individually for each concentration level. A standard likelihood ratio test was used to compare between the competing models. The analysis was carried out with the R statistical software package.

In our first comparison we partitioned the competitors into two groups, with group1 consisting of "28bp-d" and "28bp-u", and group 2 consisting of the pooled data of the other curves (75bp, 39bp, 34bp, and 31bp). We then tested for significant differences between the two groups. The table below provides the test statistic and the p-value for the likelihood ratio test (L-Ratio, P-Value) between null (m0b, no differences) and alternative (m2) model, after estimating the log likelihood of each model (LogLH). The degrees of freedom (df) were computed as the observation minus the number of model parameters. Since the cold competitor concentration has 8 levels, the compared models differ by 8 df. Although not

providing a formal hypothesis test, both model selection criteria, the (Akaikes Information Criterion (AIC) and Bayesian Information Criterion (BIC) also give lower values for m2, suggesting that this is the more appropriate model. Notice that the L-Ratio test still indicates a significant deviation from the null model after applying a Bonferroni correction (p<0.0031 after multiplying the p-value by 31).

Table 1. Comparison of 28bp-d and 28bp-u with 75bp, 39bp, 34bp, 31bp

Model	df	AIC	BIC	LogLH	L-Ratio	P-Value
m0b	17	-8.10	45.79	21.05	301.2	<0.0001
m2	25	-293.31	-214.05	171.65		

In our second comparison we tested if the competitor "34bp" behaves differently to "75bp". For this purpose, we used the data relating only to these two competitor types. Using the same modeling strategy as before, a likelihood ratio test indicates a strongly significant difference after multiple testing correction (p<0.0015 after multiplication by 15).

Table 2. Comparison of 34bp vs 75 bp

Model	df	AIC	BIC	LogLH	L-Ratio	P-Value
m0b	25	-255.33	-201.40	152.67	35.66	<0.0001
m2	33	-274.99	-203.74	170.49		

We finally tested our third hypothesis if competitor "31bp" behaves differently to "75bp". The likelihood ratio test again indicates a strongly significant difference after multiple testing correction (p<0.0015 after multiplication by 15).

Table 3. Comparison of 31bp vs 75 bp

Model	df	AIC	BIC	LogLH	L-Ratio	P-Value
m0b	17	-158.77	-122.07	96.38	106.57	<0.0001
m2	25	-249.34	-195.37	149.67		

To summarize, all three hypotheses tests provided statistically significant differences between the three considered cold competitor types. All results remained significant also after correcting for multiple testing.

References

BAUDAT, F., IMAI, Y. & DE MASSY, B. 2013. Meiotic recombination in mammals: localization and regulation. *Nature reviews. Genetics,* 14**,** 794-806.

BILLINGS, T., PARVANOV, E. D., BAKER, C. L., WALKER, M., PAIGEN, K. & PETKOV, P. M. 2013. DNA binding specificities of the long zinc-finger recombination protein PRDM9. *Genome biology,* 14**,** R35.

HULME, E. C. & TREVETHICK, M. A. 2010. Ligand binding assays at equilibrium: validation and interpretation. *British journal of pharmacology,* 161**,** 1219-37.
